# Distribution‐Agnostic Deep Learning Enables Accurate Single‐Cell Data Recovery and Transcriptional Regulation Interpretation

**DOI:** 10.1002/advs.202307280

**Published:** 2024-02-21

**Authors:** Yanchi Su, Zhuohan Yu, Yuning Yang, Ka‐Chun Wong, Xiangtao Li

**Affiliations:** ^1^ School of Artificial Intelligence Jilin University Changchun 130012 China; ^2^ Donnelly Centre for Cellular and Biomolecular Research University of Toronto Toronto ON M5S 3E1 Canada; ^3^ Department of Computer Science City University of Hong Kong Hong Kong SAR 999077 China

**Keywords:** imputation, optimal transport, single‐cell RNA sequencing

## Abstract

Single‐cell RNA sequencing (scRNA‐seq) is a robust method for studying gene expression at the single‐cell level, but accurately quantifying genetic material is often hindered by limited mRNA capture, resulting in many missing expression values. Existing imputation methods rely on strict data assumptions, limiting their broader application, and lack reliable supervision, leading to biased signal recovery. To address these challenges, authors developed Bis, a distribution‐agnostic deep learning model for accurately recovering missing sing‐cell gene expression from multiple platforms. Bis is an optimal transport‐based autoencoder model that can capture the intricate distribution of scRNA‐seq data while addressing the characteristic sparsity by regularizing the cellular embedding space. Additionally, they propose a module using bulk RNA‐seq data to guide reconstruction and ensure expression consistency. Experimental results show Bis outperforms other models across simulated and real datasets, showcasing superiority in various downstream analyses including batch effect removal, clustering, differential expression analysis, and trajectory inference. Moreover, Bis successfully restores gene expression levels in rare cell subsets in a tumor‐matched peripheral blood dataset, revealing developmental characteristics of cytokine‐induced natural killer cells within a head and neck squamous cell carcinoma microenvironment.

## Graphical abstract

The accurate measurement of genetic material encounters challenges due to limited intracellular mRNA capture, leading to many missing expression values. A distribution‐agnostic deep learning model, informed by external cues from bulk RNA‐seq data, is developed to address this issue. This model precisely reconstructs gene expression patterns, offering valuable insights into the developmental maturation mechanisms of cytokine‐induced NK cells.

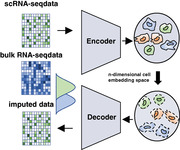

## 1 Introduction

Recent advances in single‐cell RNA sequencing (scRNA‐seq) technology have unveiled new avenues for comprehensive investigation into distinct cell types within heterogeneous tissues. This cutting‐edge technology has enabled researchers to delve into the complexities of cellular diversity, facilitating the identification of rare cellular subpopulations^[^ ^1^, ^2^, ^3^ ^]^ and uncovering developmental programs.^[^ ^4^, ^5^ ^]^ By capturing the transcriptomic profiles of individual cells at diverse developmental stages, the capacity to map cellular trajectories exposes intricate processes of cell transition and differentiation, offering valuable insights into the coordination of sophisticated biological systems. However, despite these promising applications, the current scRNA‐seq techniques are not devoid of challenges.^[^ ^6^, ^7^ ^]^ In particular, limited transcript capture and sequencing efficiency often lead to amplification noise and significant dropout events; for example, for a considerable number of genes, read counts register as zero or extremely low, complicating the differentiation between genuine biological variability and technical noise.^[^ ^8^, ^9^ ^]^ Deriving reliable information from such data requires sophisticated computational methods that can handle its complexity and inherent noise. Additionally, these methods must counteract dropout events to prevent misleading interpretations and conclusions. As such, the advancement of robust computational methodologies to address these challenges is of paramount significance.

In the past years, the field of single‐cell RNA sequencing (scRNA‐seq) has witnessed the emergence of various imputation methods focusing on the challenge of handling noisy and sparse scRNA‐seq data. These methods can be broadly categorized into two groups: model‐based and deep learning‐based approaches. Model‐based approaches can be further categorized; for instance, some methods rely on statistical models and leverage probability distribution assumptions to incorporate prior knowledge across genes and cells, allowing the estimation of true expression values.^[^ ^10^, ^11^, ^12^ ^]^ Among these methods, SAVER and scImpute^[^ ^10^, ^11^ ^]^ are representative algorithms. SAVER employs a negative binomial random variable to model gene distribution, while scImpute incorporates a combination of Gamma and Normal distributions to account for dropouts and actual gene expression levels, respectively. The other alternatives adopt a physical model to enforce fidelity between the imputed scRNA‐seq data and the observed data, often incorporating structured priors, such as the low‐rank property of gene expression matrices to regularize the solution. ALRA,^[^ ^13^ ^]^ for example, performs imputation while preserving biologically non‐expressed genes at zero expression level, applying the assumption that negative values after low‐rank approximation correspond to true biological zeros. WEDGE^[^ ^14^ ^]^ imputes gene expression values for sparse single‐cell data using low‐rank matrix decomposition. On the contrary, SCRABBLE^[^ ^15^ ^]^ recovers dropout events by employing a matrix regularization framework that takes advantage of bulk RNA‐seq data as a constraint to mitigate undesired biases. Another notable method, MAGIC^[^ ^16^ ^]^ employs data diffusion to construct a Markov transition matrix to capture valuable information from similar cells, which is then utilized to recover missing data. These diverse imputation methods offer helpful tools to address the challenges posed by amplification noise and dropout events in scRNA‐seq data. Although model‐based methods for the imputation of scRNA‐seq data are highly regarded for their interpretability, they frequently encounter substantial computational obstacles that result in prolonged processing times. As the size of scRNA‐seq datasets expands, these methods often struggle to deliver results promptly. Furthermore, the parameter adjustment process for model‐based methods might not be straightforward, contributing to the overall complexity and potentially increasing the computational burden.^[^ ^13^, ^14^, ^15^, ^16^ ^]^

Recently, deep learning methods have gained significant attention and gained popularity within the domain of single‐cell data analysis due to their remarkable learning capabilities and scalability. These methods use the prowess of deep neural networks to effectively impute missing values and elevate the quality of scRNA‐seq datasets. DeepImpute^[^ ^17^ ^]^ proposes a divide‐and‐conquer strategy that constructs multiple sub‐neural networks with dense and dropout layers to impute genes in the scRNA‐seq data. SAUCIE^[^ ^18^ ^]^ introduces regularization techniques within the auto‐encoder architecture to constrain the learned representations. DCA^[^ ^19^ ^]^ incorporates the count distribution, overdispersion, and sparsity characteristics of scRNA‐seq data by utilizing a negative binomial noise model with zero inflation. Similarly, scVI^[^ ^20^ ^]^ employs the zero‐inflated negative binomial distribution to model single‐cell data; however, what sets it apart is its incorporation of Bayesian principles and variational autoencoders to model the generation of single‐cell data. scScope^[^ ^21^ ^]^ adopts an iterative approach for imputing zero‐valued entries by utilizing a recurrent network layer, allowing the capture of temporal dependencies and improving the imputation performance. In contrast to model‐based methods, deep learning‐based approaches present several advantages. They significantly alleviate the time complexity associated with imputation tasks, resulting in faster and more resource‐efficient processing of scRNA‐seq data. However, one of the key concerns is that deep learning models are often perceived as black boxes, as they are trained using an end‐to‐end strategy, rendering them less interpretable in comparison to model‐based methods. This lack of interpretability restricts the ability to gain in‐depth insights and understanding of the biological mechanisms underlying the imputation process. Furthermore, the aforementioned deep learning‐based imputation methods rely primarily on the internal information present within the imputed expression matrix for the imputation task. This sole dependency on internal information may inadvertently exaggerate correlations between cells and genes, potentially introducing erroneous signals in subsequent analyses. It is essential that imputation methods strike a delicate balance between imputing missing values and preserving the inherent biological signals embedded within the data.

To address the previously discussed challenge, we present Bis, a distribution‐agnostic, accurate, and adaptable optimal transport‐based autoencoder model for recovering scRNA‐seq data. In contrast to conventional methodologies that lean heavily on specific probability distributions, Bis offers a universal solution. This is achieved through the fusion of an optimal transport‐based autoencoder with regularization of the cellular embedding space, which does not depend on distribution assumptions. Rather than relying on predefined distributions, the proposed approach focuses on minimizing the disparity between the unknown true data distribution and the reconstructed data distribution using an optimal transport cost. Simultaneously, this model effectively employs cellular embedding space regularization to capture the geometric and topological relationships between cells. This strategic approach ensures the extraction of biologically relevant variations while minimizing the inclusion of irrelevant noise. In addition, the intercellular correlations captured during this process are adeptly harnessed by the decoder to perform accurate missing data recovery. After that, Bis incorporates a transcriptional expression consistency module, acknowledging the consensus that average gene expression between matched bulk RNA‐seq and scRNA‐seq data exhibits distributional consistency. Our comprehensive evaluation demonstrated that Bis performs better than existing methods in several real and simulated data sets. Bis excelled in the reconstruction of scRNA‐seq data against existing state‐of‐the‐art methods and greatly improved downstream analyses, including batch effect correction, clustering analysis, differential expression assessment, and trajectory inference. Furthermore, our results highlight the capability of Bis to accurately reconstruct gene expression patterns and identify rare cell types within a head and neck tumor‐matched peripheral blood dataset. Remarkably, Bis also revealed valuable insights into developmental maturation mechanisms of cytokine‐induced NK cells in intricate head and neck squamous cell carcinoma (HNSCC) microenvironment.

## 2 Results

### 2.1 Overview of the Method

**Figure 1:**
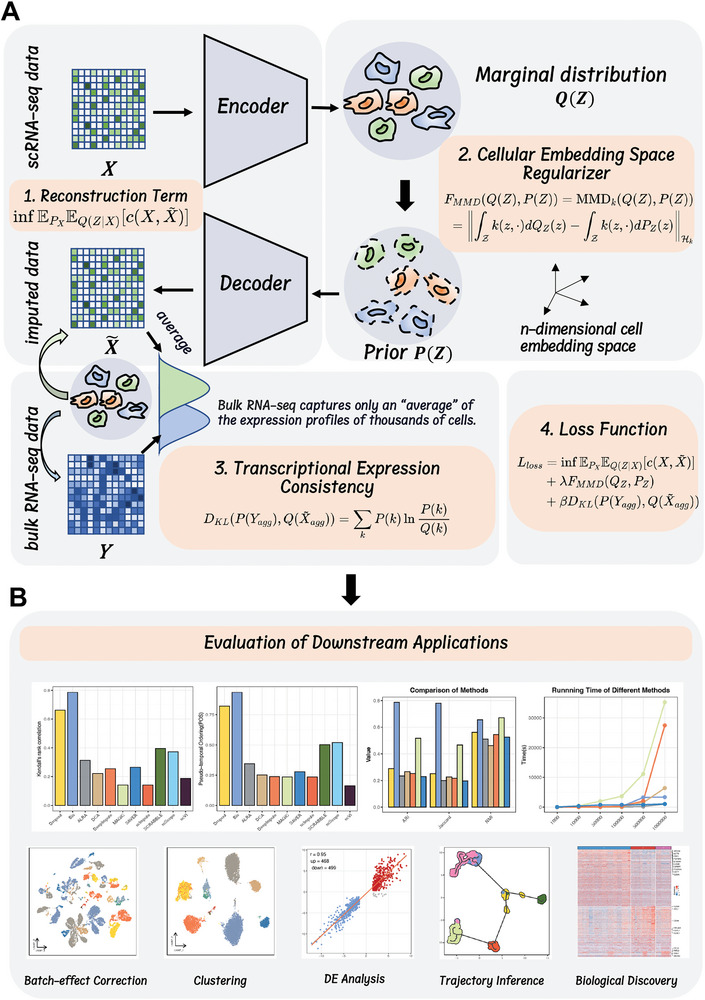
Bis is an optimal transport‐based autoencoder that regularizes the cellular embedding space, independent of specific distribution assumptions. A) Bis projects input scRNA‐seq data onto a cellular embedding latent space through an encoder with a three‐layer network structure. Then Bis minimizes the maximum mean discrepancy within the cellular embedding space. In addition, Bis reconstructs the input scRNA‐seq data employing a decoder that relies on the Wasserstein distance. Finally, Bis leverages the external prior knowledge of gene expression patterns from bulk RNA‐seq data to enhance imputation performance. B) Multiple validation experiments were conducted to evaluate the imputation performance of Bis, such as batch effect correction, clustering analysis, differential expression assessment, and trajectory inference.

The distinctiveness of Bis lies in its departure from making any distributional assumptions about single‐cell data, coupled with the capacity to incorporate external priors to guide recovery for more precise prediction (**Figure** **1**). Previous methods often predefine the noise or probability distribution of single‐cell data, frequently relying on the zero‐inflated negative binomial distribution. However, due to factors such as batch effect or experimental conditions, the noise or missing data might not conform neatly to a single probability distribution. Imposing inappropriate distributional assumptions might introduce unwarranted noise, thereby compromising data fidelity. In our study, Bis melds optimal transport theory into autoencoder architecture, dispensing with distributional assumptions. Specifically, Bis employs the Wasserstein distance to measure the divergence between the observed noisy scRNA‐seq data and the data reconstructed by the decoder. This unconstrained approach enables Bis to correct against noise and dropout events. In essence, Bis focuses on aligning foundational distributions in latent space without presuming a specific distribution for the scRNA‐seq data.

As sequencing technology continues to advance, it is progressively becoming a prevailing practice to concurrently acquire bulk RNA‐seq data alongside scRNA‐seq experiments. Bulk RNA‐seq, a representation of the averaged global gene expression, captures inter‐gene trends congruent with the corresponding scRNA‐seq data. Accordingly, we introduced a transcriptional expression consistency module that aligns decoder reconstruction, effectively obviating dropout and preserving signals by integrating matched bulk data with scRNA‐seq data. Subsequently, we conducted extensive experiments to evaluate the performance of the single‐cell data imputation. In simulations and down‐sampling experiments, we utilized both cell‐cell and gene‐gene Pearson correlation coefficients for simultaneous assessment of the quality of the imputed scRNA‐seq data. Moreover, we showcase that Bis consistently achieved precision and efficacy in several downstream analyses encompassing differential expression analysis, clustering analysis, and batch effect removal measured using specific performance metrics.

In addition, our trajectory inference evaluations revealed that leveraging prior knowledge from bulk RNA‐seq significantly refined the precision of stem cell differentiation trajectory. This was substantiated by the visualization of outcomes and quantitative metrics, including POS and Kendall's rank correlation score. Finally, in the tumor‐matched peripheral blood dataset, we explored the potential of Bis to facilitate the elucidation of biological patterns. Post‐imputation analysis using Bis revealed previously undetected cytokine‐induced NK cell states and their developmental traits.

According to references,^[^ ^22^, ^23^ ^]^ a comparison of existing imputation algorithms reveals that most of them often fail to provide stable performance, especially when it comes to enhancing downstream analysis tasks such as cell clustering and differential expression analysis. Thanks to its reliance on less strict distribution assumptions and extensive experimental validation, Bis demonstrates significant improvements in various downstream analysis tasks. In comparison to the competing algorithms, its superiority is evident in the following four aspects: 1) Bis exhibits greater robustness in handling complex and sparse noise compared to the competing algorithms due to the absence of specific data assumptions for scRNA‐seq data. This has been validated in both simulation and subsampling experiments; 2) Bis demonstrates enhanced stability when integrated into various downstream analyses. This is evidenced by its ability to improve cell subpopulation clustering, enhance differential gene expression analysis, and facilitate cell trajectory inference; 3) Bis exhibits excellent scalability, capable of efficiently processing millions of cells within a reasonable timeframe while maintaining excellent performance. 4) Bis can significantly recover lost gene expression patterns, making it beneficial for exploring new biological discoveries.

### 2.2 Bis is the Most Accurate Imputation Method on scRNA‐seq Data

We first evaluated the performance of Bis using simulated scRNA‐seq data with known ground truth. We generated simulated scRNA‐seq datasets using Splatter^[^ ^24^ ^]^ comprising 2000 cells and 500 genes across five cell types, with varying dropout rates achieved through the dropout_mid parameter (1.5, 2, 3, 4, 5, 6, 7, 8). This resulted in dropout rates of 26%, 32%, 46%, 53%, 61%, 76%, 86%, and 93%, offering a more comprehensive assessment of the method's performance across these different scenarios. Further details on these settings are available in “Section 8. (Bis is the Most Accurate Imputation Method on scRNA)' Simulated scRNA‐seq datasets Generation”. To verify the robustness of Bis, we produced ten test samples for each distinct dropout rate. The Pearson correlation coefficient (PCC) was computed to evaluate the quality of the imputed results, in relation to the gene and cell correlations.

**Figure 2:**
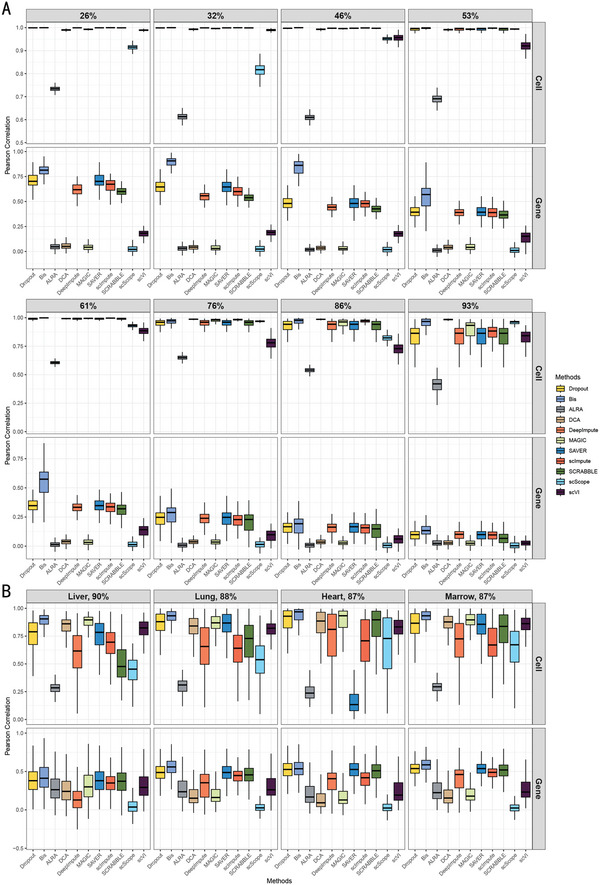
Bis is the most accurate among imputation methods on scRNA‐seq data. A) Performance evaluation of different methods using eight simulated datasets with specific dropout rates. Each boxplot represents the statistical results on ten simulated datasets. B) Performance evaluation of the methods on four real scRNA‐seq datasets.

In the first two rows of **Figure** **2**A, we observed varying degrees of improvement in cell–cell and gene–gene correlations among different methods. Notably, among these methods, Bis outperforms others such as DCA, MAGIC, and SAVER. Specifically, in terms of enhancing cell‐to‐cell correlations, the differences between methods are not substantial, which may be attributed to the relatively low dropout rate. However, in terms of improving gene‐to‐gene correlations, Bis demonstrated superior performance compared to the comparison methods, and some methods did not enhance data quality, leading to a decrease in gene‐to‐gene correlations. As dropout rates increased, we noted a heightened demand for robust and effective data recovery, a challenge Bis adeptly met. To provide a more comprehensive assessment of each method's imputation performance, we calculated the mean and variance of the Pearson correlation coefficients (PCC), with these results depicted in Figure S1 (Supporting Information). In the first two rows of Figure 1 ((Supporting Information), Bis consistently showed superior performance in gene‐to‐gene correlations, as reflected in its significantly higher mean PCC compared to other methods. The substantial improvement in Bis compared to the scRNA‐seq data with dropout is consistent with the results observed in Figure 2A. In addition, the variance of the PCC is comparable to that of comparative methods, indicating that Bis exhibits a relatively high degree of stability in the imputation process.

To comprehensively evaluate the imputation capability of the method, we performed extensive testing and statistical analysis on datasets with increased dropout rates. In the last two rows of Figure 2A, we can observe that, in terms of cell‐to‐cell correlations, as the dropout rate increases, many methods fail to maintain stable imputation, resulting in their cell–cell correlation scores falling below those observed in the original single‐cell data before imputation. Similarly, a similar trend is observed in gene–gene correlations. However, Bis still maintains a certain advantage compared to other algorithms. Remarkably, even in scenarios with high dropout rates, Bis was able to enhance gene‐to‐gene correlations effectively. Similarly, we also calculated the mean and variance of PCC, as illustrated in Figure S1 (Supporting Information). As can be observed, the mean values of most methods either remain constant or are significantly lower than those of the data with dropout. In contrast, Bis demonstrated a notable improvement in performance relative to the dropout data, a consistency that is in line with the patterns observed in Figure 2A. After a comprehensive and detailed evaluation, our results clearly demonstrate that Bis effectively addresses the challenges posed by dropout in scRNA‐seq data, surpassing the performance of current state‐of‐the‐art algorithms.

Due to the challenges in procuring a gold standard for true expression levels of scRNA‐seq data, we conducted down‐sampling experiments to evaluate the effectiveness of the imputation methods. We generated the reference dataset (Table S1, Supporting Information) and the down‐sampling dataset^[^ ^25^ ^]^ with dropout events using the simulated method detailed in ref. [10]. Similarly, we then computed both gene‐wise Pearson correlation across cells and the cell‐wise correlation across genes between the reference and down‐sampling datasets, as well as between the reference dataset and recovered datasets, to assess the effectiveness of each method. Figure 2B shows the results. Across all four datasets, Bis achieved the best performance compared to the other methods. Notably, for the downsampled datasets, Bis enhanced both gene‐wise and cell‐wise correlations on all datasets, whereas DeepImpute, ALRA, and scScope usually performed worse.

From the above results, Bis outperforms algorithms that assume specific probability distributions (DCA, scVI, SAVER, and scImpute). This observation underscores the superior performance and generalizability of Bis, stemming from its distribution‐agnostic modeling approach.

### 2.3 Bis Improves Cell‐Type Identification and Generates Better Visualization on scRNA‐seq Data

**Figure 3:**
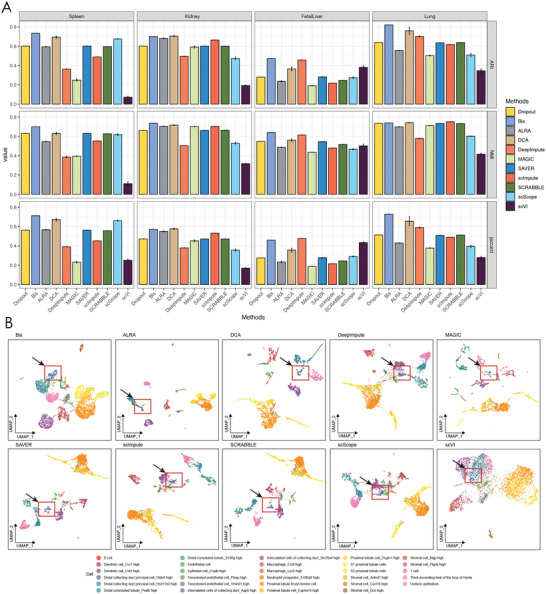
Bis improves both visualization and cell type identification of scRNA‐seq data. A) ARI, NMI, and Jaccard scores of the clustering results of different methods on four real datasets. B) plots of UMAP visualization of Kidney data after imputation with various methods.

Clustering is a crucial step in the analysis of scRNA‐seq data and reveals the complexity of tissue systems (e.g., identifying cell types and studying cellular heterogeneity). In this section, we evaluated the impact of several imputation methods on unsupervised clustering, thus indirectly comparing the effectiveness of each imputation method. All imputation techniques were tested on the four real datasets with clustering carried out using Seurat, as in ref. [26]. As some methods require randomization, we performed each imputation method ten times on each of the four datasets^[^ ^27^, ^28^ ^]^ and then calculated the average value of each metric. As shown in **Figure** **3**A, our method consistently outperformed the competing methods across most of the datasets. Although our method has slightly lower performance on some datasets, its performance is acceptable due to a very small gap. In addition, compared to the other methods, Bis demonstrates consistently superior performance across these four datasets.

To provide a visual view of the clustering improvement achieved by Bis, we show the 2D UMAP results on the Kidney dataset in Figure 3B (results for other datasets can be found in the Figure S2, Supporting Information). Upon applying different imputation methods to the raw data with dropout events, we observe a distinct separation of specific cell types. Remarkably, Bis demonstrates exceptional performance in terms of visualization quality, particularly in scenarios where cell populations are closely intermingled and difficult to differentiate. A notable example is observed in the Kidney dataset, where Bis successfully identified the distal collecting duct principal cell population, characterized by the expression of Hsd11b2 and Cldn4 (Figure 3B). However, other algorithms, including DeepImpute, scScope, and scVI, tend to misclassify these two cell subtypes with other cell types. While ALRA, SAVER, and SCRABBLE can distinguish these two cell subtypes, they introduce other cell types, resulting in less accurate clustering results. The Hsd11b2 gene is intricately linked to glucocorticoid regulation, which is instrumental in prenatal stress‐induced fetal programming. It is implicated in a heightened risk of preterm birth and subsequent increased susceptibility to metabolic and neurodegenerative diseases in adulthood.^[^ ^29^ ^]^ On this basis, Bis contributes to a better understanding of cellular subpopulations and their functional implications. Furthermore, the UMAP visualization results of other methods on the remaining datasets (as shown in Figure S2A, Supporting Information) highlight their limitations in achieving similar clustering improvements. In particular, other algorithms struggled to effectively separate densely subpopulated immune cell subtypes, such as T cells (marked in grey), Monocytes (marked in cyan), and NK cells (marked in green), which are hard to differentiate owing to their overlapping gene expression patterns. However, Bis separated these cell populations well, allowing a better characterization of the unique molecular signatures. Another significant achievement of Bis is that it has the capability to distinctly segregate Dendritic cells from leukocytes, a task that generally poses challenges due to overlapping characteristics. The enhanced clustering effect achieved by Bis becomes evident when comparing its visualization results with those of alternative methods.

### 2.4 Ablation Study and Hyperparameter Selection

**Figure 4:**
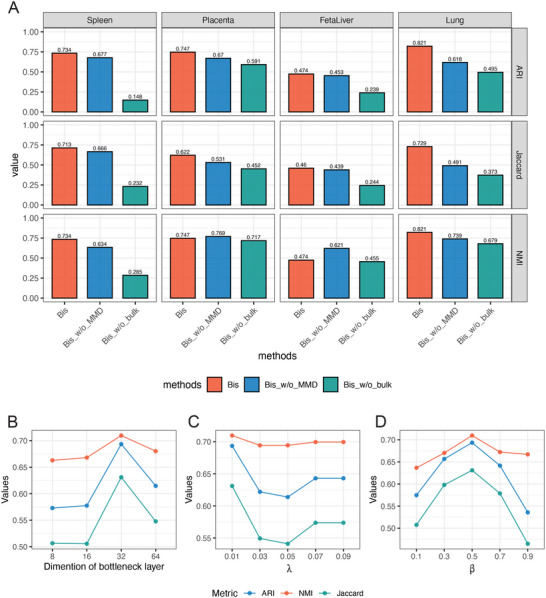
A) Ablation study measured by ARI, NMI, and Jaccard Index on four real datasets. Parameter analyses of (B) dimension of bottleneck layer and loss hyperparameters: C) λ and D) β controlling the behavior of the loss function.

We removed essential components of the proposed model to test their impact on the quality of the model and to investigate how Bis benefited from these components. We specifically ablated each component as follows: 1) removed the loss item of the cellular embedding space regularizer in the loss function, called Bis_w/o_MMD; 2) removed the loss item constraining the consistency between scRNA‐seq and bulk RNA‐seq data, called Bis_w/o_bulk. We evaluated ablated and the whole algorithms and recorded the ARI, NMI, and Jaccard index metrics on the four real datasets (Spleen, Kidney, FetalLiver, and Lung). **Figure** **4**A depicts the results. We observed that both loss items are beneficial in improving clustering performance. Prior knowledge provided by bulk RNA‐seq data, in particular, improved clustering more efficiently than the cell embedding space regularizer. The explanation for this might be that bulk RNA‐seq data is not as sparse as single‐cell RNA‐seq data, and the gene patterns retained in it are precise and abundant resulting in higher performance. In summary, both aspects of Bis are reasonable and valid.

Then, we studied the impact of specific parameter settings in the proposed models. In the model, three parameters are involved: the dimension of the bottleneck layer, λ, and β. The dimension of the bottleneck layer represents the dimensionality of the middle layer in the autoencoder, while λ and β are hyperparameters in the loss function. For a more detailed definition, please refer to subsection Loss Function and Optimization of Methods. First, to investigate the impact of each parameter on the results, we systematically controlled and explored the influence of each parameter individually. Thus, to demonstrate the effect of the dimension of the bottleneck layer, we imputed the four real datasets (Spleen, Kidney, FetaLiver, and Lung) with varying sizes of bottleneck layers, 8, 16, 32, and 64. Then, we adopted Seurat to cluster the imputed results, and the average metric results (ARI, NMI, and Jaccard) of the four datasets are shown in Figure 4B. We observe that the values of the three metrics increased rapidly from size 8 to 32, reaching an optimal value at 32, then gradually decreased from sizes 32 to 64. Selecting a too‐low or high‐dimensional bottleneck layer size decreases the ability of Bis to separate the different cell types. After that, we explore the impact of λ since λ controls the contribution of the cellular embedding space regularizer to recovery. We performed Bis on the four real datasets with values λ of 0.01, 0.03, 0.05, 0.07, and 0.09 and then clustered the imputed results using Seurat. Figure 4C shows the average ARI, NMI, and Jaccard index with different λ. We observe that when λ increased from 0.01 to 0.05, the three metrics decreased sharply. Although the metrics improved as the value of the λ increased from 0.05 to 0.09, the performance of λ = 0.01 remained the best. Subsequently, we conducted the same experiment as with λ, with the value of β from 0.1 to 0.9 to show the significance of the external prior of the bulk RNA‐seq data. Figure 4D depicts the average results. From the results, we observe that the ARI, NMI, and Jaccard index showed maximum values at β = 0.5, respectively. Finally, we systematically combined values for the three parameters to gain a more comprehensive understanding of the potential impact of interactions among parameters on the final results. We conducted tests on four datasets, examining variations in three clustering metrics across 100 different parameter combinations (Table S2, Supporting Information). The results are presented in Table S3, (Supporting Information). Upon careful examination of the results presented in Table S3 (Supporting Information), we acknowledge that increasing the “layer” and “β” parameters within the tested range contributes to the improvement of clustering metrics while enlarging parameter “λ” has the opposite effect.

### 2.5 Bis Can Facilitate the Removal of Batch Effects in Human Pancreatic Data from Different scRNA‐seq Protocols

Batch effects are unwanted technical variations in data due to processing cells from different batches. They can originate from multiple sources, including using different sequencing protocols and laboratory practices and biological factors like different tissue types, species, or inter‐individual variations. Given the diverse and complex factors that contribute to the emergence of batch effects, the presence of dropout events in single‐cell data further complicates accurate downstream analysis. Fortunately, several studies^[^ ^30^, ^31^ ^]^ have indicated that improving data quality enhances the performance of batch removal methods and adequately mitigates batch effects. On this basis, we tested the impact of several imputation algorithms on batch effect removal using a widely used pancreas dataset comprising 16,382 cells and 19,093 genes. The dataset was obtained from multiple sequencing platforms, including CEL‐seq,^[^ ^32^ ^]^ CEL‐seq2,^[^ ^33^ ^]^ Fluidigm C1,^[^ ^34^ ^]^ SMARTer^[^ ^35^, ^36^ ^]^ inDrop,^[^ ^37^ ^]^ and Smart‐seq.^[^ ^38^ ^]^ A comprehensive overview of the dataset is provided in Tables S4 and S5 (Supporting Information). We applied the imputation algorithms to impute this dataset and then employed Harmony^[^ ^39^ ^]^ incorporated within the scanpy package^[^ ^40^ ^]^ to perform batch effect removal. It is worth noting that the SAVER and scImpute algorithms are excluded from the comparison group because the processing time exceeds 24 h.

**Figure 5:**
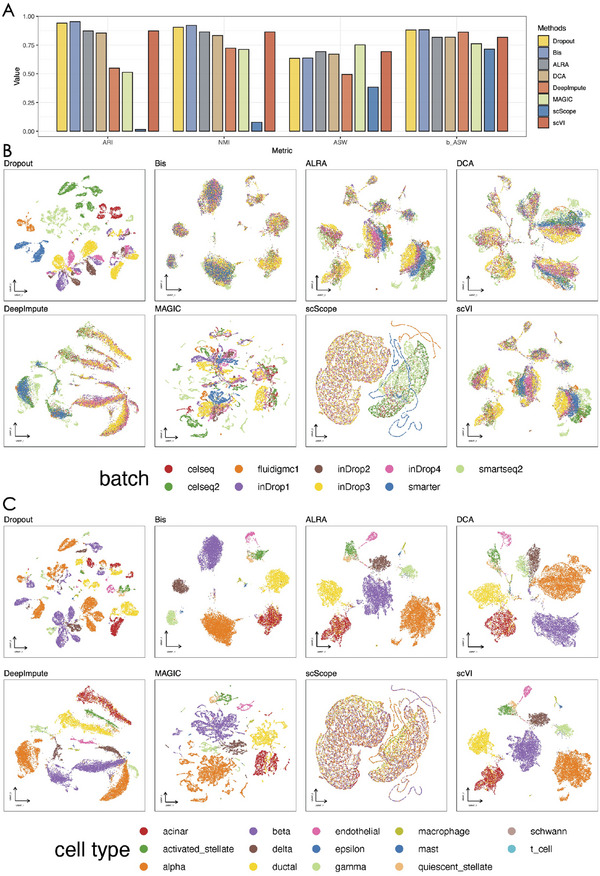
Bis can facilitate the removal of batch effects in human pancreatic data generated from different scRNA‐seq protocols. A) shows the Adjusted Rand Index (ARI), normalized mutual information (NMI), relative distances (cell‐type ASW), and the average silhouette width (ASW) across batches scores after batch effect removal of the different methods on the pancreas dataset. B) illustrates uniform manifold approximation (UMAP) and projection layouts. The data points are color‐coded according to batch annotation. C) illustrates uniform manifold approximation (UMAP) and projection layouts. The data points are color‐coded according to cell identity.

**Figure** **5** presents the comparison of the different imputation algorithms. Figure 5A illustrates that Bis outperforms on both ARI and NMI metrics, indicating its ability to facilitate congruous clustering of analogous cell types across diverse batches. Notably, while Bis did not always have the highest ASW metric, it excelled in the b_ASW metric‐specially tailored to assess batch effects from a batch‐centric perspective, reflecting proficiency in integrating data from diverse scRNA‐seq procedures. In Figure 5B, we observe that, with the exception of MAGIC and scScope, the other algorithms contributed to the removal of batch effects to varying degrees. MAGIC could amalgamate scRNA‐seq data from specific protocols but struggled to consistently cluster identical cell types across batches, a clear example being the alpha cells. Conversely, scScope blurred the distinction between cell types and batches, intensifying rather than mitigating batch effects. Methods ALRA, DCA, DeepImpute, and scVI, could reduce batch effects quite well but fell short in clustering consistency, as seen with the fragmentation of alpha and beta cells into numerous minor clusters. In contrast, the recovery performed by Bis demonstrated favorable outcomes in both batch effect removal and cell clustering. For instance, activated and quiescent stellate cell subtypes exhibit well‐clustered patterns while preserving subtype heterogeneity. The visualizations align with the performance metrics and we can conclude that Bis promotes batch effect removal, indirectly indicating its superiority in terms of reconstruction results compared to the other algorithms.

### 2.6 Bis Can Identify Rare Cell Types and Small Cell Types that are not Identified by Other Methods

**Figure 6:**
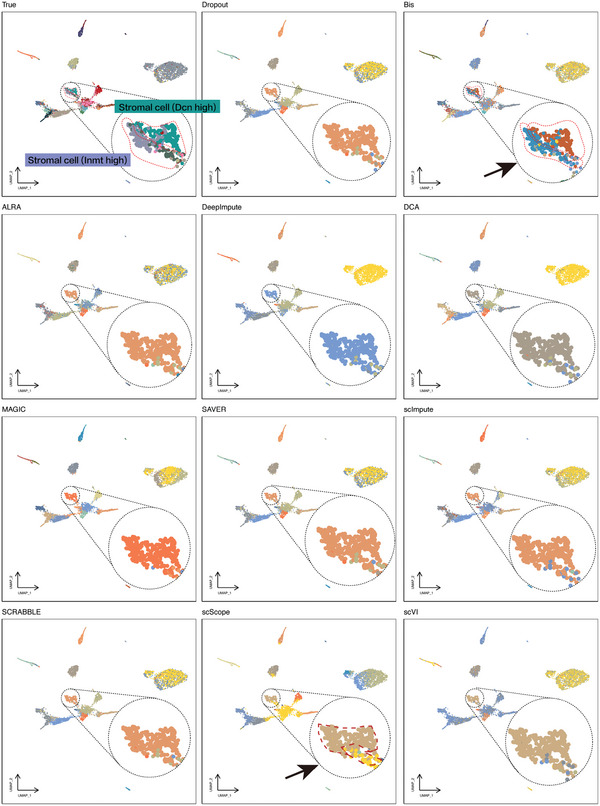
Bis improves the identification of cell subpopulations. The figure illustrates the annotation of clustered labels obtained from imputed data on the raw data with dropout events.

To investigate if Bis can detect rare cell types and small clusters that other methods may not be able to detect, we compared visualization of the clustering results from the recovered data using each completed method. **Figure** **6** presents the visual view of clustering labels for the lung dataset. In this figure, “True” refers to the actual cell clustering labels from the lung dataset labeled on this UMAP, while “Dropout” represents the clustering labels obtained by clustering the raw, unimputed scRNA‐seq data on the same UMAP. This dataset was sourced from mouse lung tissue and encompasses 9,116 cells spanning 32 distinct cell types. Among these, there are several cell subtypes, such as stromal cells that can be further divided into two distinct populations based on high expression of Dcn or Inmt. The stromal cells hold pivotal roles in a spectrum of physiological and pathological functions, as described in ref. [41]. They provide structural support to tissues, regulate extracellular matrix composition, and modulate immune responses. Stromal cells also participate in tissue repair and regeneration, as well as the formation of new blood vessels (angiogenesis). In addition, they can communicate with neighboring cells, including parenchymal cells, immune cells, and blood vessels through direct cell–cell interactions and the secretion of soluble factors. Naturally, the identification of stromal cells in different states plays a crucial role in understanding these biological processes. From Figure 6, we observe that directly preprocessing the sequencing data with dropout events and then clustering them does not differentiate between the two distinct cell subtypes. This suggests that the critical expression pattern information of these two cell subtypes was lost during the sequencing process. Following recovery with Bis and subsequent clustering, the cluster results become consistent with the true cell‐type annotations, highlighting that Bis effectively recovers the key differential expression information to differentiate these two cell subtypes. It is also worth noting that clustering with the expression data imputed by the scScope algorithm can identify certain heterogeneous cell subtypes (Figure 6). While it categorizes the majority of cells into one group, there is a substantial discrepancy compared to the true cell type annotations, resulting in a significant margin of error. Despite the ability of scScope to assist in the discovery of cellular heterogeneity, its accuracy remains insufficient and introduces some level of error. On the contrary, the other comparative algorithms cannot identify cell subpopulations from differential expression patterns. In summary, Bis can better recover the transcriptional levels of cell subpopulations exhibiting differential expression, thus improving the performance of cell subtype identification.

### 2.7 Bis can Efficiently Handle Large‐Scale scRNA‐seq Data

With the ever‐growing size of single‐cell RNA sequencing (scRNA‐seq) datasets, imputation methods need to demonstrate excellent scalability. To evaluate the scalability of Bis, we analyzed the largest scRNA‐seq dataset available to date, comprising nearly one million kidney cells with matched bulk RNA‐seq data obtained from 10X Genomics.^[^ ^42^ ^]^ Due to note that due to constraints in computational resources and time, some of the existing methods such as MAGIC, SAVER, scImpute, and SCRABBLE, were unable to perform imputation on this large‐scale dataset. Therefore, the comparison focused on the remaining five methods.

**Figure 7:**
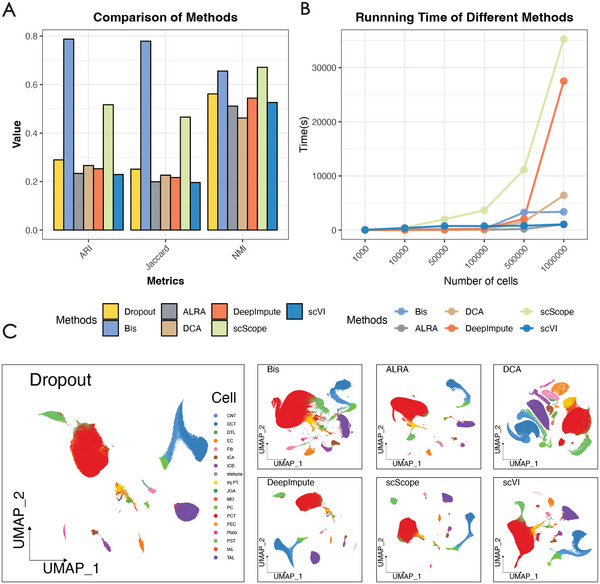
Bis can efficiently handle large‐scale scRNA‐seq data. A) ARI, NMI, and Jaccard scores of the clustering results of different methods on nearly one million kidney cells. B) runtimes for the imputation of several matrices with different numbers of cells down‐sampled from one million kidney cells. C) UMAP visualization of one million kidney cell data after imputation with the methods.

The result, depicted in **Figure** **7**A, clearly demonstrates the superiority of Bis over the four other methods in terms of adjusted Rand index (ARI) and Jaccard score, which ranked consistently in the top two methods. Although scScope slightly outperformed Bis in terms of normalized mutual information (NMI), the difference is not substantial. Conversely, the other methods introduced more noise, thereby impeding clustering performance. To visually highlight the clustering performance, we present UMAP visualization of the raw data and recovered results obtained using Bis in Figure 7C. Bis effectively separates the many cell types, including successfully clustering rare cells such as macular densa (MD) cells. Additionally, we evaluated the scalability of the methods by measuring their respective running time as depicted in Figure 7B. Bis exhibits a moderate runtime and is significantly faster than DeepImpute, and scScope is the fastest‐running method. With the satisfactory clustering performance achieved by Bis, processing a million‐cell dataset in less than an hour is both acceptable and competitive. In conclusion, our proposed method Bis significantly enhances cell clustering performance through the recovery of missing single‐cell gene expression and demonstrates remarkable computational efficiency.

### 2.8 Bis Improves Differential Gene Expression Analysis

Differential expression analysis aims to identify genes whose expression levels are significantly altered by specific variables, such as cell type or state. The identification of these differentially expressed genes (DEGs) offers invaluable insights into the underlying molecular mechanisms contributing to phenotypic variations. However, the performance of differential analysis methods is frequently compromised by dropout events observed in scRNA‐seq data. We conducted a performance comparison of imputation methods by evaluating their performance in identifying differentially expressed genes. Particularly, we focused on SCRABBLE, a method that leverages prior knowledge derived from bulk RNA‐seq data to ensure a fair and robust basis for comparative evaluations. The dataset consists of six samples of bulk RNA‐seq, with four samples representative of human embryonic stem cells (H1 ESC) and two samples representing definitive endoderm cells (DEC). Additionally, the dataset comprised 350 samples of scRNA‐seq, with 138 DEC cells and 212 H1 ESC cells. To identify genes that are differentially expressed between DEC and H1 cells after performing imputation, we utilized DESeq2^[^ ^43^ ^]^ as the analysis tool. Subsequently, we curated a list of biologically upregulated and downregulated genes from the results, with the criteria of “padj⩽0.05”, “log2FoldChange⩾1” and “baseMean⩾10”.

**Figure 8:**
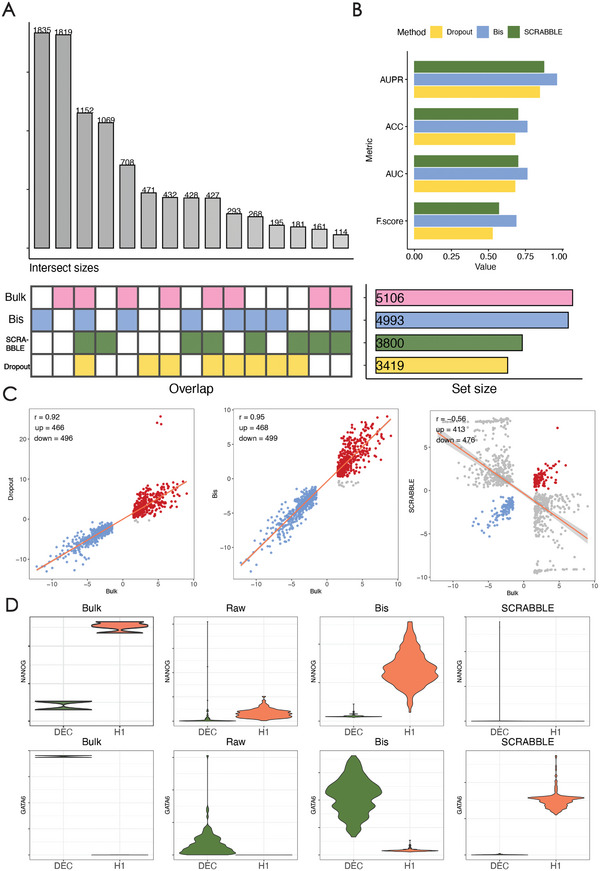
Bis improve differential expression analysis. A) shows the correspondence of differentially expressed genes (DEGs) between bulk and single‐cell RNA‐seq with different imputation approaches. B) The bar plots depict DEG detection performances from raw and imputed scRNA‐seq datasets, evaluated against the gold standard defined by the bulk RNA‐seq dataset. C) The correlations between the log fold‐changes of DEGs identified from bulk RNA‐seq and single‐cell RNA‐seq datasets. D) The violin plot depicting the expression distribution of the marker genes NANOG and GATA6 in DEC and H1 cells.

The raw scRNA‐seq data exhibits a considerably higher rate of zero expression compared to bulk RNA‐seq data (49.1% vs 14.8%) and shared a smaller number of differentially expressed genes (432 DEGs) with bulk samples (**Figure** **8**A). However, after performing imputation, the number of DEGs increased, approaching the number of DEGs identified in bulk samples. Among the imputation methods, Bis demonstrated the highest number of dataset‐specific DEGs (1835) and shared a significant proportion of DEGs (708) with bulk RNA‐seq (Figure 8A). To provide a more comprehensive evaluation of DEG detection using scRNA‐seq data, we employed multiple metrics including area under Precision‐Recall curve (AUPR), accuracy (ACC), F‐score (also recognized as F1‐score or F‐measure), and the area under the receiver operating characteristic curve (AUC), to evaluate the performance of DEG detection in the imputed scRNA‐seq datasets using DEGs and identified the bulk RNA‐seq as the reference standard. In Figure 8B, we can observe that Bis achieves the highest scores for all four metrics. In addition, to evaluate the consistency of the identified DEGs between imputed scRNA‐seq and bulk RNA data, we selected the top 1000 highly‐variable genes, comprising 500 up‐regulated genes and 500 down‐regulated genes from the bulk RNA‐seq data as the reference. The Pearson correlation coefficient was used to assess the correlation between these two sets of data. The results are presented in Figure 8C. The Bis‐recovered scRNA‐seq data exhibited the highest correspondence with the bulk RNA‐seq data, evidenced by the largest number of shared top‐1000 DEGs and the highest correlation of fold‐changes compared to bulk RNA‐seq data. This suggests that the reconstruction of missing single‐cell gene expression performed by Bis effectively captured the expression patterns present in the scRNA‐seq data. In contrast, the results from the SCRABBLE algorithm exhibited a considerable number of misidentified differentially expressed genes (gray points). This suggests that the reconstruction of missing single‐cell gene expression performed by Bis effectively captured the expression patterns present in the scRNA‐seq data.

Moreover, we selected ten marker genes (GATA6, LEFTY1, EOMES, SOX17, and CXCR4 for DEC cells, NANOG, ZFP42, DNMT3B, SO2, and POU5F1 for H1 cells) to facilitate a comprehensive comparison of expression patterns between bulk RNA data and imputed scRNA‐seq data. Figure 8D depicts the results for the maker genes GATA6 and NANOG and results for other marker genes can be found in Figures S5 and S6A (Supporting Information). From Figure 8D, we observe that when bulk RNA data was the reference, the expression level of the marker gene NANOG in H1 cells was not effectively recovered using SCRABBLE. However, Bis successfully recovered the expression of the NANOG gene in the imputed scRNA‐seq data. In H1 cells, the GATA6 gene is expressed at a low level. Still, the normalized counts imputed by SCRABBLE were slightly higher than the bulk and raw data, potentially introducing undesirable information into H1 cells. In contrast, Bis avoids the occurrence of over‐imputation. Similar results can be observed in Figures S5 and S6A (Supporting Information). Further, we mapped cells onto the UMAP space, overlaying the expression of signature genes (Figures S3 and S4, Supporting Information), and found that Bis effectively recovers the expression patterns of H1 and DEC cells, but also avoids introducing additional noise variables. This further demonstrates the effectiveness of Bis in recovering differentially expressed genes.

Lastly, we investigated whether the recovery of differentially expressed genes (DEGs) after imputation retained biological relevance within the functional genome. We focused on 1835 genes specifically associated with Bis that were absent from the bulk RNA‐seq data, the dropout data, and the imputed data from other algorithms. We performed gene ontology enrichment analysis and KEGG pathway analysis using clusterProfiler^[^ ^44^ ^]^ to explore the biological significance of those specific genes. Figures S6B, and S7A,B (Supporting Information) summarize the 20 GO terms in the categories biological processes (BP), molecular functions (MF), and cellular components (CC)), sorted by p‐value. We observe that the top enriched GO terms are highly correlated with metabolism, stem cell development, and differentiation. Key terms include regulation of RNA metabolic process (GO:0051252), stem cell differentiation (GO:0048863), and embryo development (GO:0009790). Additionally, significant enrichment was observed for terms like embryo development ending in birth or egg hatching (GO:0009792), RNA localization (GO:0006403), condensed nuclear chromosome (GO:0000794), and nucleotide binding (GO:0000166) (Figures S7A,B, Supporting Information), which are pertinent to the proliferation and mitosis of human pluripotent stem cells. Previous studies have demonstrated the importance of these terms in stem cell development and differentiation. For example, stemness and differentiation of human embryonic stem cells are influenced by environmental factors, such as hypoxic stress (GO:0006950, response to stress).^[^ ^45^ ^]^ In addition, our KEGG pathway analysis, depicted in Figure S6C (Supporting Information), revealed that many genes are enriched in pathways related to environmental information processing, metabolism, and organismal systems, crucial for the cellular activities of hESCs.^[^ ^46^, ^47^, ^48^ ^]^ Subsequently, we visualized the distribution of identified differentially expressed genes in various biological process pathways using heatmap visualization, as illustrated in Figure S7C (Supporting Information). Our findings demonstrate a significant enrichment of numerous DEGs in pathways of biological processes associated with hESCs metabolism, development, and differentiation, such as pathways regulation of nucleobase‐containing compound metabolic process (regulation of nucleobase‐containing compound metabolic process), and response to stress (GO:0006950),^[^ ^46^, ^47^, ^48^ ^]^ among others. This outcome signifies the consistency of the biological significance in the gene expression data post‐imputation with Bis recovery.

To further explore the biological relationship between these DEGs and transcriptional regulation, we constructed a chord diagram to display the associations of DEGs with pathways involving embryonic appendage morphogenesis and stem cell differentiation, as depicted in Figure S7D (Supporting Information). We have observed a substantial enrichment of genes in both the ‘stem cell differentiation’ and ‘embryonic appendage morphogenesis’ pathways, reflecting the regulatory mechanisms in the differentiation of H1 cells into definitive endoderm (DE) cells. Specifically, a marked upregulation of the TBX5 gene within the ‘stem cell differentiation’ pathway was evident. This finding is consistent with existing literature, highlighting TBX5's role in embryonic tissue and organ formation. TBX5, responsible for producing the T‐box 5 protein, is essential for the development of upper limbs and the heart, particularly in forming the heart's septum and its electrical conduction system, as shown in Figure S7E (Supporting Information).^[^ ^49^, ^50^ ^]^ In addition, previous research^[^ ^51^ ^]^ has suggested that in vertebrate embryonic development, CDX2 becomes active in endodermal cells that are posterior to the developing stomach.^[^ ^52^, ^53^ ^]^ Subsequently, we visualized the expression distribution of the CDX2 gene (Figure S7F, Supporting Information). Our data, recovered using Bis, reveal an upregulation trend of CDX2 during the transition from H1 to DE cells, a pattern not discernible in scRNA‐seq data with dropout events. These findings highlight the superior capability of Bis in recovering critical regulatory genes and uncovering potential regulatory patterns.

We then conducted additional analysis to elucidate the relationship between the identified differentially expressed genes (DEGs) and transcription factors (TFs). First, we took the 1835 DEGs and utilized the NetworkAnalyst platform^[^ ^54^ ^]^ to search for TFs from the JASPAR^[^ ^55^ ^]^ database. The resulting TF‐gene network was filtered to remove nodes with a ‘degree’ value of less than 30, ensuring high‐quality TFs in the network. Figure S8 (Supporting Information) illustrates the network, which consists of 62 nodes. In particular, we identified FOXC1 among TFs, which has been implicated in the regulation of embryonic and ocular development.^[^ ^56^ ^]^ Additionally, SRF was found, which encodes a ubiquitous nuclear protein that stimulates both cell proliferation and differentiation.^[^ ^57^ ^]^ We also found GATA2/3, which are lineage‐specific markers in trophoblast differentiation.^[^ ^58^, ^59^ ^]^ These results suggest the potential involvement of these TFs in orchestrating the gene expression patterns associated with the identified DEGs.

### 2.9 Bis Enhances the Inference of Cellular Trajectories

The dynamic and continuous processes of cells, including cell differentiation, cell cycle progression, and response to external stimuli, often give rise to inherent variations in gene expression. To identify and analyze these differences in gene expression over time, trajectory inference offers a suitable method. Typically, trajectory inference involves three main steps. First, dimensionality reduction techniques are applied to reduce the complexity of the scRNA‐seq data and to select the informative features that capture the underlying dynamics. Second, a trajectory structure is constructed to represent the developmental or temporal progression experienced by the cells. Third, each cell is projected onto the trajectory based on its evolutionary trajectory along the process. However, the presence of dropout events, which are common in the scRNA‐seq data, is often ignored by most existing trajectory inference algorithms. Given that dropout events can result in missing or underestimated expression values, which can have an impact on the precision of trajectory inference, imputing scRNA‐seq data prior to inferring the cellular trajectory might be a viable approach to enhance the precision of pseudotime placement.

To evaluate the impact of different imputation methods on the reliability of pseudotime ordering, we utilized a time‐course scRNA‐seq dataset^[^ ^48^ ^]^ that captures the differentiation process of H1 ESC to definitive endoderm cells (DECs). The dataset consists of 758 cells, profiled at specific time points: 0, 12, 24, 36, 72, and 96 h after initiating the differentiation of H1 ESCs. We applied Bis and ten other imputation methods to the raw scRNA‐seq data including the known time points. After imputing the data, we reconstructed the trajectories using the Mococle3 algorithm,^[^ ^60^ ^]^ a widely used method for inferring trajectories in single‐cell RNA sequence data. We utilized recovered data and reconstructed the trajectories to assess the reliability of different imputation methods for pseudo‐temporal ordering.

**Figure 9:**
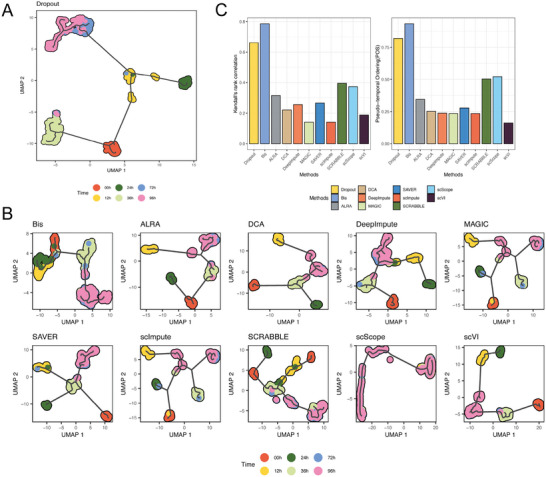
Bis enhances the inference of cellular trajectory. A) The trajectories reconstructed by monocle3 from the raw scRNA‐seq data with dropoutb events. B) The trajectories reconstructed by Monocle3 from the imputed scRNA‐seq data using all methods. C) Kendall's rank correlation scores and Pseudo‐temporal Ordering(POS) to assess the precision between true time labels and inferred orderings derived from the imputed scRNA‐seq data.

**Figure** **9**A presents the trajectories reconstructed by monocle3 from the raw scRNA‐seq data with dropout events. We observed that the differentiation time points in the reconstructed trajectories appeared disordered and did not align with the known differentiation trajectory of ESCs. Figure 9B demonstrates that recovery using Bis yielded the highest correspondence between the inferred pseudotime and the real‐time course of differentiation, suggesting that Bis can effectively capture the underlying patterns and dynamics of gene expression changes during cellular differentiation. An intriguing observation is an overlap between the differentiated cells at 72 and 96 h, which is consistent with previous reports,^[^ ^48^ ^]^ and likely due to these cells sharing similar transcriptomes, suggesting similar gene expression patterns and potential functional similarities during the differentiation process. To provide a more precise and quantifiable comparison, we utilized Pseudo‐temporal Ordering (POS) and Kendall's rank correlation scores^[^ ^61^, ^62^, ^63^, ^64^ ^]^ for evaluating the accuracy between the true time labels and the inferred orderings derived from the imputed data, as depicted in Figure 9C. We observed that Bis achieved the highest POS and Kendall's rank correlation scores among all evaluated imputation methods, indicating its superior accuracy in capturing the temporal progression of differentiation.

**Figure 10:**
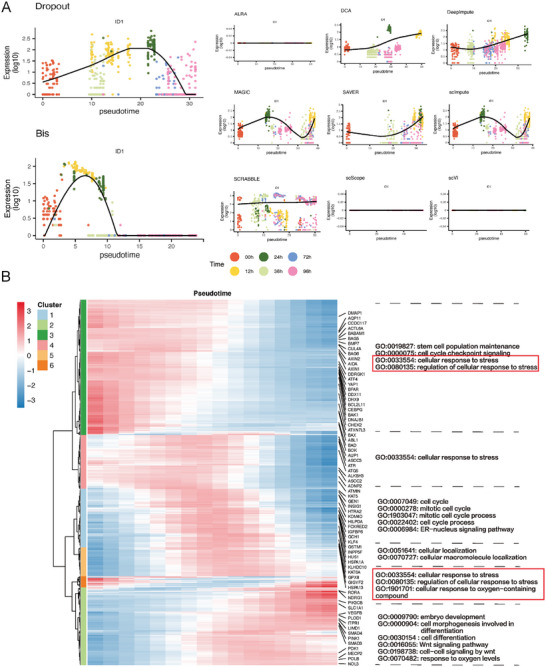
Bis enhances the inference of cellular trajectory. A) The expression dynamics of a key DE marker, ID1, is shown in pseudotime order. The key DE markers of differentiation at 24h, 36h, 72h, and 96h imputed by all methods are provided in Figures S15–S22 (Supporting Information). B) Gene expression heat map and identified Gene Ontology (GO) terms of each cluster.

We investigated the expression dynamics of signature genes related to pluripotency and definitive endoderm cells. Interestingly, we found that Bis improved the understanding of the dynamics of gene expression after reconstruction, as depicted in **Figure** **10**A and Figures S15–S22 (Supporting Information). Specifically, at the 12‐h time point of differentiation, the results showed that a significant proportion of cells exhibited strong expression of ID1 in response to treatment with BMP4, Activin A, and CHIR 99021 (a WNT signaling agonist).^[^ ^48^ ^]^ At the 24‐h time point of differentiation, a second wave of genes, including MSX2 (Figure S15, Supporting Information), displayed significantly increased expression levels. This upregulation of gene expression indicates a transition of cells toward a primitive streak state. At the 36‐h time point of differentiation, there was a rapid decrease in the level of T transcripts, characterized by upregulation of early DE‐specific genes, such as CER1 and GATA4 (Figures S16 and S17, Supporting Information). At the 72‐h time point of differentiation, a majority of the cells exhibited high expression levels of endogenous DKK4 and MYCT1 (Figures S18 and S19, Supporting Information). This indicates the activation of specific signaling pathways and the induction of gene expression programs associated with cell fate determination and further differentiation processes. The expression patterns of these genes suggest the progression of cells toward specific lineage commitment and the acquisition of cell type‐specific characteristics. Both CXCR4 and SOX17 genes (Figures S20 and S21, Supporting Information) exhibited subtle but significant upregulation as early as 36 h of differentiation, which continued to increase at later time points. Based on these results, Bis demonstrated better performance compared to other imputation methods for accurately capturing the expression patterns and dynamics of these signature genes. This highlights the effectiveness of Bis in preserving and recovering important gene expression information during the imputation process. Moreover, the restored gene expression patterns unveil the regulatory mechanisms inherent in the authentic process of embryonic developmental differentiation. Bone morphogenetic protein (BMP) signaling is known to protect the pluripotency of embryonic stem cells and is essential for in vivo mesoderm formation.^[^ ^65^, ^66^, ^67^ ^]^ The regulatory mechanism underlying this involves an unexpected capability of BMP, through ID1, to hinder differentiation by sustaining the expression of the cell adhesion molecule E‐Cadherin (Cdh1).^[^ ^65^, ^68^ ^]^ Additionally, BMP acts through ID1 to impart a proximal posterior identity on epiblast cells, priming them for mesodermal fates while temporarily restraining them from overt mesoderm differentiation.^[^ ^69^, ^70^ ^]^ This entire process aligns with the upregulation trend of ID1 during the differentiation of stem cells into the mesoderm (Figure 10A (Bis)). Subsequently, the downregulation of the ID1 gene at 72 and 96h is attributed to the tightly regulated process of cardiogenic mesoderm specification in bipotent mesendoderm progenitors, involving an antagonistic interplay between ID proteins and the Acvr1b signaling pathway.^[^ ^71^ ^]^ The regulatory mechanism of high Acvr1b signaling to suppress the expression of ID genes is externally manifested as the differentiation of mesendoderm progenitors toward endoderm. Conversely, attenuation of Acvr1b signaling in these cells derepresses ID gene transcription, promoting cardiogenic mesoderm specification.

Lastly, we performed gene ontology (GO) enrichment analysis on the recovered scRNA‐seq data to delve deeper into the regulatory mechanisms present at each differentiation stage. This analysis enabled us to explore the enriched biological pathways and functional annotations associated with genes at each stage, providing a more comprehensive understanding of the regulatory patterns after imputation. We first utilized the “graph_test” function in monocle3 to identify differentially expressed genes before and after imputation. We then selected 6,929 genes that were differentially expressed only after imputation with Bis, not detected in the raw scRNA‐seq data. Subsequently, we performed gene clustering on the selected genes using the R package “pheatmap”, which uses a cluster number of six and the ward.D2 clustering method. Next, we conducted gene ontology (GO) enrichment analysis on the genes within each cluster using the R package clusterProfiler,^[^ ^44^ ^]^ allowing us to identify the enriched biological processes, molecular functions, and cellular components associated with the genes in each cluster. The relevant results are depicted in Figure 10B. We discovered that pathways related to oxygen and stress (GO:0070482: response to oxygen levels and GO:0033554: cellular response to stress)^[^ ^72^, ^73^ ^]^ were multiple clusters, which were not detected in the raw scRNA‐seq data. Existing literature^[^ ^74^, ^75^ ^]^ reports that environmental stress such as hypoxia, oxidative stress, heat stress, and mechanical stress can activate specific transcription factors, leading to gene‐specific expression changes. For instance, hypoxic stress promotes the differentiation of embryonic stem cells (ESCs) toward mesoderm by activating HIF‐1α. Regarding mechanical stress, high stiffness tends to promote mesoderm differentiation, while low stiffness promotes ectodermal differentiation through the regulation of YAP1^[^ ^76^, ^77^ ^]^ (Figure S22, Supporting Information).

### 2.10 Bis can Reveal the Developmental Characteristic of Cytokine‐Induced Memory‐Like Natural Killer Cells in the Circulating Immune Microenvironment

After conducting a comprehensive assessment of Bis in comparison to other competing methods, we embarked on investigating whether the reconstruction capabilities of Bis could enhance the potential for biological discovery. This exploration aimed to uncover insights that might otherwise remain inaccessible or considerably more challenging to attain without the utilization of imputation techniques.

**Figure 11:**
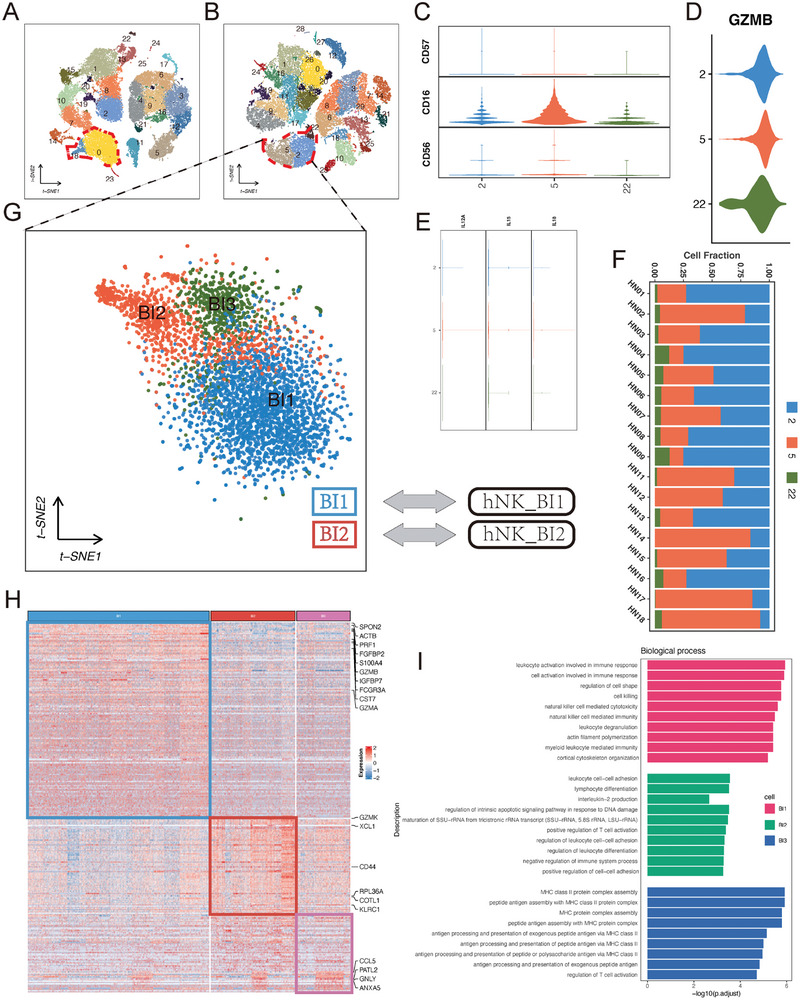
Bis can reveal the developmental characteristics of cytokine‐induced memory‐like natural killer cells in the circulating immune microenvironment. A) t‐SNE plots visualizing the peripheral blood dataset before imputation colored by cell clusters. B) t‐SNE plots visualizing the peripheral blood dataset after reconstruction by Bis. C) violin plots depicting the expression distribution of the surface markers CD56, CD16, and CD57 in human natural killer (NK) cells. D) violin plots depicting the expression distribution of the marker gene GZMB. E) violin plots depicting the expression distribution of the interleukin‐12 (IL‐12), IL‐15, and IL‐18. F) proportions of the three cell types 2, 5, and 22 in the peripheral blood of 17 patients. G) t‐SNE plots visualizing 4794 cytokine‐induced memory‐like (CIML) NK cells colored by cell clusters. H) Heatmaps of selected differentially expressed genes in the three cell subsets. I) Top enriched Gene Ontology (GO) terms related to the distinct cell subsets in terms of biological processes.

We applied Bis to the peripheral blood dataset comprising 36030 cells obtained from freshly resected head and neck squamous cell carcinoma (HNSCC) tumors. The cohort included 17 treatment‐naive patients (six human papillomaviruses (HPV)^+^ and 11 HPV^−^) (Table S9, Supporting Information). In the original study,^[^ ^78^ ^]^ clustering using Seurat identified two natural killer (NK) cell clusters labeled as clusters 0 and 18 (**Figure** **11**A). However, after applying Bis imputation to this dataset and performing clustering, we observed that there were three distinct subpopulations of NK cells (Figure 23, Supporting Information), labeled as clusters 2, 5, and 22 in Figure 11B. Analysis of the expression profiles of these NK cell subsets revealed that they fell within the *CD*56^*dim*^ *CD*16^+^ *CD*57^−^‐ phenotype (Figure 11C). Further investigation into the specific cell subsets revealed a cytokine‐induced memory‐like phenotype, characterized by elevated expression of granzyme B mRNA (Figure 11D) and preactivation through exposure to IL‐12, IL‐15, and IL‐18 (Figure 11E). Figure 11F demonstrates the distribution of these three cell types across all samples.

To further demonstrate the characteristics of the three NK subtypes, we analyzed their extracted expression profiles from 4,794 NK cells. Similarly, we used Seurat to perform dimensionality reduction and clustering of selected cytokine‐induced NK cells and visualized these cells for a comprehensive overview as shown in Figure 11G using t‐distributed stochastic neighbor embedding (t‐SNE), labeled as clusters BI1, BI2, and BI3, respectively. Then, we utilized the FindAllMarkers() function in Seurat to identify differentially expressed genes within these clusters. Three hundred and twenty‐seven genes displayed significant differential expression between the three subsets (169 more strongly expressed in BI1, 133 in BI2, and 25 in BI3) and served as markers for all the human blood cytokine‐induced memory‐like (CIML) NK cells (Figure S25, Supporting Information). A heatmap representation (Figure 11H) of the 4794 CIML NK cells and the 327 differentially expressed genes in the NK subsets highlighted the heterogeneity among BI1, BI2, and BI3 clusters.

Comparing the top differentially expressed genes in the three CIML NK cell subsets, we observed distinct expression patterns (Figure S24, Supporting Information). We observed that BI1 cells exhibited higher expression of genes such as FGFBP2, GZMB, SPON2, CST7, IGFBP7, PRF1, and FCGR3A, which is consistent with previous research findings.^[^ ^79^ ^]^ Similarly, BI2 cells had elevated expression of genes including GZMK, XCL1, CD44, and COTL1, in accord with results of previous studies.^[^ ^79^ ^]^ Hence, we can confirm that BI1 and BI2 correspond to the previously identified hNK_Bl1 and hNK_Bl2 cell subsets,^[^ ^79^, ^80^ ^]^ respectively, while BI3 represents a novel CIML NK subset. It can be observed that the differentially expressed genes in the BI3 cell subtype include CCL5, GZMH, HLA‐DPA1, and HLA‐DPB1, among others (Figure 11H; Figure S25, Supporting Information). Through an extensive review of existing studies,^[^ ^81^, ^82^, ^83^, ^84^, ^85^, ^86^ ^]^ we found that this cell subtype represents an intermediate state in the development of CIML NK cells from a naive to a mature state, and it has been previously named hNK_Bm4 in the existing literature.^[^ ^81^ ^]^ The analysis of the biological processes using the R package clusterProfiler^[^ ^44^ ^]^ revealed enrichment in cytolysis in BI2 cells, significant enrichment in response to cytokines and regulation of cytokine production functions in the BI1 subset, and the BI3 subset showed an intermediate cell state and is responsible for the regulation of NK cytolysis (Figure 11I and Figures S28 and S29, Supporting Information).^[^ ^87^, ^88^, ^89^ ^]^ To ensure a fair and accurate comparison of the results and to validate the identification of the three NK cell subtypes, we conducted repeat experiments with adjusted ‘resolution’ parameters of the FindClusters function using the original data, as detailed in Figure 26 (Supporting Information). The results revealed that, although increasing the resolution allowed the original data to cluster NK cells into three subtypes (Figures S26 and 27, Supporting Information), we were able to definitively identify only two subtypes, hNK_BI1 and hNK_BI2, through biomarker analysis (Figure 27C,D, Supporting Information), and the existence of the BI3 subtype could not be confirmed (Table S10–S17, Supporting Information). Simultaneously, we conducted a more thorough investigation of the impact of the “resolution” parameter on the Bis‐imputed data. The results (Supplementary Figure 30) indicate an improvement in the quality of the Bis‐imputed data, significantly aiding in the identification of rare subtype cells. Additionally, the results of the differential analysis in Tables S18–S27 (Supporting Information) demonstrate the detection of corresponding biomarkers for the three NK cell subtypes at resolutions ranging from 0.1 to 1.2 (highlighted in red in the tables), even at lower resolutions. These findings further highlight the excellent capability of Bis in capturing cell heterogeneity and accurately restoring crucial missing expression patterns.

**Figure 12:**
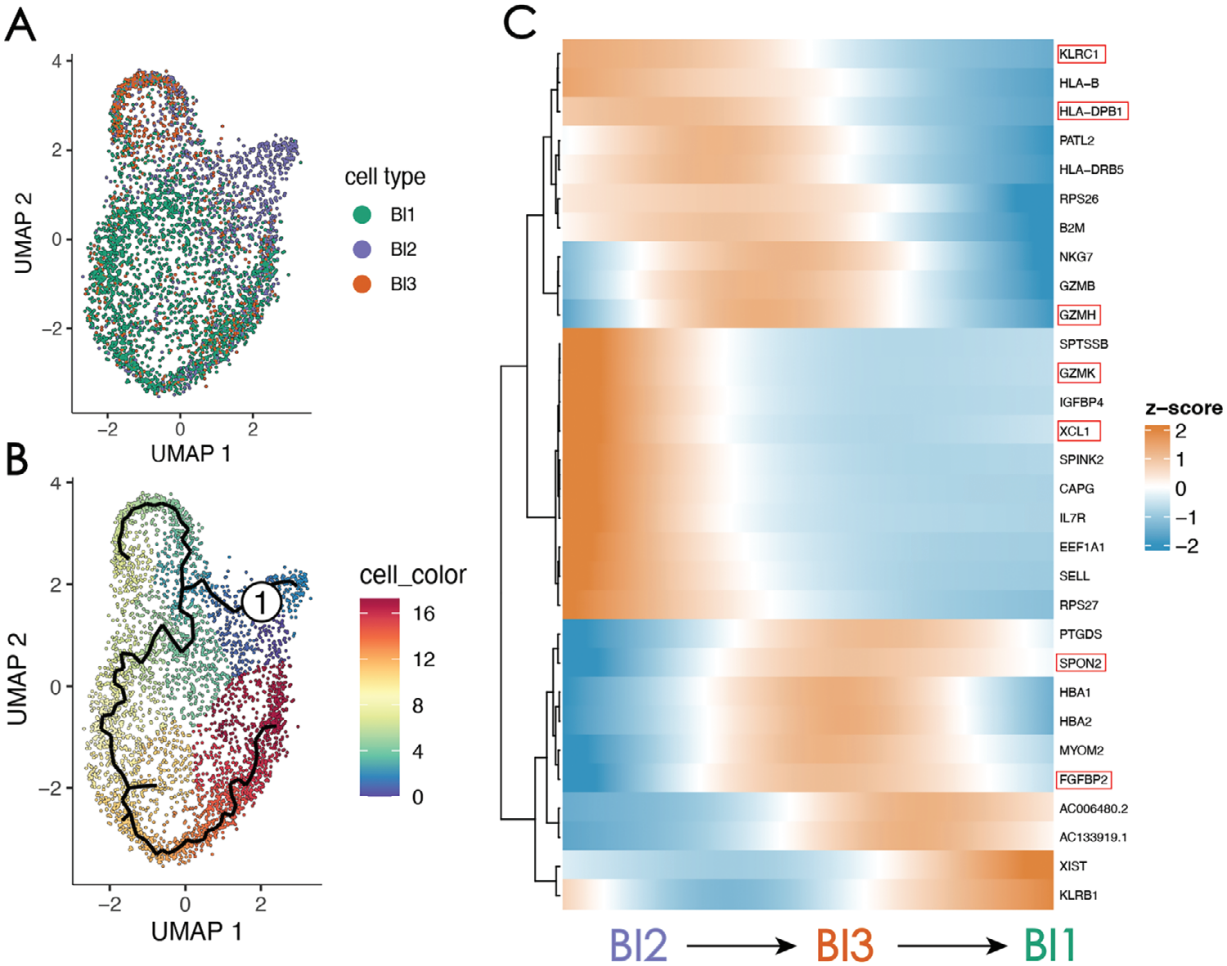
Bis can reveal the developmental characteristics of cytokine‐induced memory‐like natural killer cells in the circulating immune microenvironment. A) the trajectory inferred using the recovered cytokine‐induced memory‐like single‐cell gene‐expression data. The number of cells is 4,794 and the cells on the trajectory are assigned to colored clusters (cell subsets BI1, BI2, and BI3). B) a trajectory tree where cells are colored by pseudotime. C) Heatmap showing the gene expression dynamics during CIML NK cell development. Genes (rows) are clustered and cells (columns) are ordered according to pseudotime development.

To further investigate the role of newly identified BI3 cells in cytokine‐induced NK cell activation, we performed a pseudo‐time analysis using the Monocle3 package in this subset. The results are depicted in **Figure** **12**. In Figure 12A,B, the three cell subsets exhibited sequential developmental trajectories over time, corresponding to BI2 (in the cytolytic state), BI3 (intermediate cell states), and BI1 (supports cytotoxic activity). In (Figure 12C) the heatmap demonstrated different expression patterns of the three subsets. Moreover, differentially expressed genes identified by Monocle 3 in each subset align with those identified in the previous Seurat analysis (Figure 11H).

Together, our analyses suggest that the expression profiles recovered by the Bis method are amenable to biological interpretation and capture well the information related to the developmental patterns of tissue‐specific gene expression.

## 3 Conclusion

We propose a distribution‐agnostic deep learning model, called Bis, to address the dropout events prevalent in scRNA‐seq data. Bis enables the recovery of missing expression values associated with dropout events, accommodating various data distributions, and utilizes external priors from bulk RNA‐seq data to refine the reconstruction process. One of its core mechanisms involves minimizing the disparity between the unknown data distribution and the reconstructed data distribution by employing an optimal transport cost. Simultaneously, to achieve more efficient feature encoding in the cellular embedding space to better characterize cellular heterogeneity, we introduce a regularization method based on Maximum Mean Discrepancy (MMD). Comprehensive investigations involving both simulated and real datasets indicated that in comparison to raw scRNA‐seq data, the recovered data generated by Bis exhibited enhanced preservation of cell type identification and consequently yielded superior results in downstream analyses. When analyzing tumor‐matched peripheral blood datasets, our model provided a more detailed portrayal of CIML NK cell status.

One of the standout features of Bis is its user‐friendliness, which manifests in three crucial ways. First, unlike previous methods, Bis makes no assumptions about dataset distribution. The increasing variation in sequencing technologies combined with unpredictable experimental errors, has often limited the efficacy of prior algorithms. In contrast, Bis is built on a foundation of adaptability, allowing it to function regardless of the actual dataset distribution. Second, Bis easily integrates with a range of existing pipelines, including Seurat, Scanpy, and Monocle, as well as standard downstream analyses used in scRNA‐seq data. These encompass tasks like batch effect removal, clustering, differential expression analysis, and trajectory inference. Notably, tools specifically designed to analyze the recovered scRNA‐seq data by Bis demonstrated enhanced performance compared to methods created for raw scRNA‐seq data (as detailed in the ‘Results’ Section). For instance, Bis adeptly identified cell‐related expression patterns, recovered pivotal genes differentially expressed in specific cell types, and greatly improved the precision of trajectory inference. Furthermore, by the recovery of these genes, Bis is able to explore the functions and roles of different cell types during cellular differentiation, which provides insights into key pathways and their associated regulatory mechanisms.

Moreover, Bis demonstrated the capacity to incorporate external priors. As the proportion of missing values increases, the imputation task becomes increasingly challenging. Compared to sparse single‐cell data, matched bulk RNA‐seq data exhibit a lower dropout rate, providing a more reliable source of prior information about gene correlations. This preexisting knowledge significantly contributes to the retrieval of absent gene expression values. In addition, Bis is poised to revolutionize the paradigm of analyzing both single‐cell and bulk RNA‐seq data simultaneously. Traditionally, these two distinct data types were frequently examined in isolation, or single‐cell datasets were employed to aid in deconvoluting bulk data. However, Bis bridges the gap by seamlessly integrating them. This integration not only offers a more comprehensive understanding of biological systems at the single‐cell resolution but also opens new avenues for research and discovery. Researchers can now delve into the intricate relationships between single‐cell and bulk data, illuminating complex biological phenomena that were previously challenging to unravel.

An additional crucial feature of Bis is its scalability, particularly when dealing with a large number of cells. Unlike traditional model‐based algorithms, Bis exhibited significant scalability and speed benefits within a reasonable timeframe. Furthermore, Bis offers a speed advantage over deep learning‐based methods, in addition to a significant performance improvement.

In summary, Bis stands apart from traditional methods by steering clear of assumptions regarding data structure or distribution. It introduces a novel approach that combines optimal transport cost and external priors from bulk RNA‐seq data for joint recovery of scRNA‐seq data. As a distribution‐agnostic tool in the realm of single‐cell biology, Bis offers versatile functionality, facilitating a wide range of tasks in single‐cell data analysis, including batch effect correction, clustering extensive datasets comprising millions of cells, differential expression analysis, trajectory inference, and the interpretation of joint clinical data. Collectively, these attributes position Bis as a state‐of‐the‐art method in the field of single‐cell RNA sequencing data analysis. We are committed to ongoing updates and enhancements to the framework, with the expectation that Bis will continue to uncover valuable applications in single‐cell multi‐omics data analysis.

## 4 Experimental Section

### scRNA‐seq Data Processing and Normalizing

In the work, the matrix $X_{raw}\in R^{m\times n}$ represented the raw single‐cell gene expression matrix, where the rows represent *m* cells and the columns denoted *n* genes. The matrix $Y_{raw}\in R^{m\times n}$ represented the raw bulk gene expression matrix, in which rows represent *m* samples and columns denoted *n* genes. To keep the feature dimension consistent, the intersection of genes was selected between the scRNA‐seq and the bulk RNA‐seq datasets. After that, both datasets were preprocessed, and the processed gene expression matrix was represented as $X^{m\times n}$ and $Y^{m\times n}$. The preprocessing process was as follows: 1) Cells with fewer than 200 genes were filtered out, and genes observed in fewer than three cells were also filtered for scRNA‐seq data. 2) The total counts of each cell were normalized using the *scanpy.pp.normalize_total* function in the scanpy^[^ ^40^ ^]^ package in Python. 3) Log‐normalization of all datasets were performed with an offset of one using the *scanpy.pp.log1p* function.

### A Distribution‐Agnostic Encoder/Decoder Framework

Previous deep learning methods based on auto‐encoders (AEs) or variational auto‐encoders (VAEs) for imputation typically presume that single‐cell transcriptome data correspond to a specific probability distribution, such as ZINB. Naturally, the loss function was then created to assess certain discrepancy measured between the reconstructed probability distribution $P(\tilde{X})$ and the presumed true distribution P(X), where X was the processed gene expression matrix and $\tilde{X}$ was reconstructed scRNA‐seq data by the decoder. However, given the variety of single‐cell transcriptome data sequencing platforms and technologies, data distribution assumptions would undoubtedly limit the model's scalability and applicability in practical scenarios. As a result, the assumption that no particular probability distribution is fitted to the single‐cell transcriptome data was relaxed and then ask: *was it still possible to measure the difference between the two probability distributions to construct a more generalized deep imputation model while the true data distribution was unknown?*

Optimal transport theory provided a solution to this question.

First, the distance loss of the network was redefined between the two distributions using optimal transport divergence, formulated as:

(1) $$L_{\mathrm{OT}}\left(P\left(X\right),P\left(\tilde{X}\right)\right))=\underset{\pi }{\inf}\;\int {\int \;}_{(X,\tilde{X})\in \Gamma }\pi \left(x,\tilde{x}\right)c\left(x,\tilde{x}\right)dxd\tilde{x}$$

where $c\left(x,\tilde{x}\right):X\times X\to R_{+}$ is any measurable cost function, $\Gamma \in P(X\approx \;P\left(X\right),\tilde{X}\approx \;P\left(\tilde{X}\right))$ is a set of all joint distributions of $\left(X,\tilde{X}\right)$ with marginals P(X) and $P(\tilde{X})$, respectively and $\pi \left(x,\tilde{x}\right)=\pi \left(x|\tilde{x}\right)p\left(\tilde{x}\right)=\pi \left(\tilde{x}|x\right)p\left(x\right)$ is the joint probability density.

The key to calculating this loss was to find the set of joint distributions Γ, which was very difficult. Inspired by the idea of feature space transformation for transfer learning, an indirect method was employed to compare the difference between the two probability distributions, that was, to measure it in the feature space of samples. On the other hand, latent variable models like VAEs share similar principles. They aim to capture the underlying structure of the data by introducing latent variables, which were unobserved variables that were assumed to generate the observed data. Generally, these models were defined by a two‐step procedure, where first a code Z was sampled from a fixed distribution P(Z) on a latent space Z and then Z was mapped to the scRNA‐seq data $X\in R^{d}$ with a (possibly random) transformation. The latent space Z was assumed to capture the underlying structure of the scRNA‐seq data, while the transformation from Z to X captured the variability in the data. The goal of the model was to learn the distribution P(Z) and the transformation, such that the reconstructed data $\tilde{X}$ approximates the true scRNA‐seq data X.

Based on the above two points, it could be concluded that the optimal transport loss could be reduced to a simpler form instead of finding a coupling Γ in (1) between two random variables living in the X space, one distributed according to P(X) and the other according to $P(\tilde{X})$, allowing the optimal transport loss to be computed without the assumption of a probability distribution for the scRNA‐seq data X. The following theorem provides theoretical support for this inference process. Please refer to ref. [90] for the detailed proof of Theory 1.

Theorem 1 ([90]) For $P_{G}\left(\tilde{X}|Z\right)$ for all z∈Z, where $G:Z\to \tilde{X}$, we have

(2) $$\begin{array}{ccc} L_{\mathrm{OT}}\left(P\left(X\right),P\left(\tilde{X}\right)\right) & = & \underset{P\in P\left(X\approx P\left(X\right),\tilde{X}\approx \;P_{G}\right)}{\inf}E_{(X,\tilde{X})\approx \;P}\left[c\left(X,\tilde{X}\right)\right]\\ & = & \underset{Q:Q(Z)=P(Z)}{\inf}E_{P_{X}}E_{Q(Z|X)}\left[c\left(X,\tilde{X}\right)\right] \end{array}$$

where Q(Z) is the marginal distribution of Z when X∼P(X) and Z∼Q(Z|X) .

This result allowed to optimize over random encoders Q(Z|X) instead of optimizing all couplings between X and $\tilde{X}$. This was useful because it reduces the complexity of the optimization problem and makes it more tractable. By optimizing over random encoders Q(Z|X) instead of all couplings, we can learn a more compact and expressive representation of the scRNA‐seq data. Additionally, this approach allowed to leverage the flexibility of neural networks to model the mapping from X to Z, which could capture non‐linear relationships and complex patterns in the data. Furthermore, this allowed for a more general solution, and it did not rely on the specific form of the distribution of the data. As a result, it made the model more robust for imputing scRNA‐seq datasets produced from different types of sequencing platforms.

Although problem (3) differs from problem (1) in that it was not necessary to assume a specific probability distribution for scRNA‐seq data to make the model universal, the presence of constraints on *Q* made model optimization difficult. Fortunately, such constrained optimization problems had many well‐developed solutions. A common approach in convex optimization theory to solving such problems was to relax them to an unconstrained optimization problem.

Namely, used any convex penalty $F:Q\to R^{+}$: such that F(Q(Z),P(Z))=0 if and only if Q(Z)=P(Z), and for any λ > 0, the relaxed unconstrained version of the problem (3) is formulated as:

(3) $$L_{OT}\left(P\left(X\right),P\left(\tilde{X}\right)\right)=\inf E_{P_{X}}E_{Q(Z|X)}\left[c\left(X,\tilde{X}\right)\right]+\lambda F\left(Q\left(Z\right),P\left(Z\right)\right)$$

So far, the main part of the loss function of the probability distribution‐agnostic reconstruction network of single‐cell transcriptome data was presented using optimal transmission theory at this point. The first term was a reconstruction loss based on a given optimal transmission cost to ensure that the recovered scRNA‐seq gene matrix $\tilde{X}$ was as consistent as possible with the raw X. The second part was a regularizer that balances the prior distribution with the probability distribution of the cell features projected to the hidden space. In the following subsection, implementation of this approach will be discussed.

### Cellular Embedding Space Regularizer

The regularization term $F(Q_{z},P_{z})$, as previously stated, aims to measure the difference between the cell embedding feature distribution $Q_{z}$ and the prior probability distribution $P_{z}$. For single‐cell imputation tasks, maximizing the utilization of intercellular correlations could significantly aid in zero‐value recovery. Traditional algorithms often directly utilize raw, noisy cell features to borrow correlation information, which undoubtedly introduced more bias to recovery results. AE and VAE‐based methods map the original data into a latent space for cell feature encoding, recharacterizing, and capturing cellular correlations. Naturally, the efficiency of encoding affected the utilization of correlation information. For VAE‐based models, approximating the posterior probability Q(Z|X) to the prior $P_{z}$ may result in different cell types having latent encodings that were close to each other, undoubtedly introducing bias. Conversely, using a continuous mixture distribution 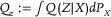 to match $P_{z}$ avoided this issue, enabling different cell types to obtain more heterogeneous latent encodings, thereby allowing for a more accurate reconstruction of the gene expression matrix based on correlations.

There were many different ways to measure the distance between two probability distributions, each with its advantages and disadvantages. Some of the most common ways to measure the distance between two probability distributions were Kullback‐Leibler divergence (KL divergence), Jensen‐Shannon divergence, Bhattacharyya distance, and so on.

In single‐cell omics, the maximum mean discrepancy (MMD) had been adopted as a distance metric in batch correction methods to align the distribution of the data across different experiments or platforms.^[^ ^18^, ^91^ ^]^ For example, it could be used in combination with dimensionality reduction methods such as t‐SNE or UMAP to visualize the distribution of cells in a lower‐dimensional space and to identify patterns and subpopulations of cells that were not obvious in the high‐dimensional data. Researchers could use this information to understand the molecular mechanisms underlying cell differentiation and disease.

Given its prior successful applications, the decision was made to use MMD to measure distribution differences. Specifically, MMD compares two probability distributions Q(Z) and P(Z) by measuring the distance between the mean embeddings of the distributions. The basic idea was to compute the distance between the means of the two distributions in a reproducing kernel Hilbert space (RKHS), which is formulated as:

(4) $$\begin{array}{ccc} F_{MMD}\left(Q\left(Z\right),P\left(Z\right)\right) & = & {\text{MMD}}_{k}\left(Q(Z),P(Z)\right)\\ & = & {\left\|\int_{Z}k\left(z,\cdot \right)dQ_{Z}\left(z\right)-\int_{Z}k\left(z,\cdot \right)dP_{Z}\left(z\right)\right\|}_{H_{k}} \end{array}$$

where $H_{k}$ is the RKHS of real‐valued functions mapping Z→R, and *k* is a positive‐definite reproducing kernel Z×Z→R. Here, the inverse multi quadratics kernel $k\left(x,y\right)=C/(C+∥x-y{∥}_{2}^{2})$ with $C=2d_{z}\sigma_{z}^{2}$ was used, which was the expected squared distance between two multi‐variate Gaussian vectors drawn from $P_{Z}$.

Overall, the advantages of using a cellular embedding space regularizer are as follows:

- cell embedding space regularizer could provided more efficient feature encoding and capture fine‐grained differences in the underlying distribution of cell types, even when the number of cells was small. These cell‐cell correlation priors, lying in the cellular embedding space, were useful for scRNA‐seq data imputation.
- MMD was a non‐parametric method for measuring the distance between two probability distributions, which made it a robust and flexible module for incorporating a general autoencoder framework.
- Single‐cell genomics data was typically high‐dimensional and could included hundreds of thousands of genes for each cell. MMD can effectively handle this high‐dimensional data and can be used to identify patterns and similarities in the data that would be difficult to detect using traditional methods.

### Transcriptional Expression Consistency Module

As the sparsity of single‐cell transcriptome data increases, the available cellular or genetic correlation information within the data decreases, making it more difficult to recover. To overcome this challenge, incorporating external a priori information, such as gene co‐expression networks or cellular annotation information, can be an effective solution. However, such prior knowledge was not always readily available.

Bulk RNA sequencing was the method of choice for transcriptomic analysis of pooled cell populations, tissue sections, or biopsies. It measured the average expression level of individual genes across hundreds to millions of input cells and allowed for the identification of differentially expressed genes between different conditions and treatments. It had several advantages.

- Bulk RNA sequencing was relatively inexpensive and easy to perform, making it a cost‐effective option for large‐scale transcriptomic studies.
- Bulk RNA sequencing allowed for the generation of large amounts of data that could be used to study the global gene expression patterns of a complex tissue or organism to identify new biological pathways and mechanisms.
- Bulk RNA‐seq typically requires milligrams of total RNA, the data generated were an average of the entire population of cells present in the sample. This means that any noise or variability present in the data was likely to be averaged out, resulting in a more robust signal.

For many scRNA‐seq data sets, there were usually existing bulk RNA‐seq data available for the same cell or tissue type. As the technology for scRNA‐seq had advanced and had become more widely used, it was growing increasingly common to collect matched bulk RNA‐seq data when a new scRNA‐seq experiment was performed. Since sequencing was from the same tissue, there was a consistency between the average gene expression of the true scRNA‐seq data and the average gene expression of the bulk RNA‐seq data.

Bulk RNA‐seq data provided a global view of the transcriptomic profile of a mixed population of cells and could be used to provide a comprehensive understanding of the gene expression patterns of pooled cell populations. This information could be used as prior information to help guide the recovery of missing values in scRNA‐seq data, which provided a more detailed view of the gene expression patterns.

Based on these considerations, a transcriptional expression consistency module was introduced to guide the recovery for further enhancements. Specifically, the probability distributions should be similar due to the average consistency of gene expression between bulk RNA‐seq and scRNA‐seq data. Following this assumption, Kullback‐Leibler divergence was adopted to measure the difference between the probability distribution $P({\tilde{X}}_{agg})$ and $P(Y_{\mathrm{agg}})$ formulated as:

(5) $$D_{KL}\left(P\left(Y_{agg}\right),Q\left({\tilde{X}}_{agg}\right)\right)=\sum_{k}P\left(k\right)\ln\frac{P(k)}{Q(k)}$$

where $P({\tilde{X}}_{agg})$ denotes the average expression of each gene across all cells for scRNA‐seq data, $P(Y_{\mathrm{agg}})$ denotes the average expression of each gene across all samples for matched bulk RNA‐seq data, and probability distribution of each gene k is represented as P(k).

### Loss Function and Optimization

The three components of the proposed model have already been discussed. As a result, the final loss function can be written as:

(6) $$\begin{array}{ccc} L_{\mathrm{loss}} & = & \inf E_{P_{X}}E_{Q(Z|X)}\left[c\left(X,\tilde{X}\right)\right]+\lambda F_{\mathrm{MMD}}\left(Q_{Z},P_{Z}\right)\\ & & +\;\beta D_{\mathrm{KL}}\left(P\left(Y_{\mathrm{agg}}\right),Q\left({\tilde{X}}_{\mathrm{agg}}\right)\right) \end{array}$$

where λ and β are all hyper‐parameters. The first term was a reconstruction loss based on a given optimal transmission cost. Here, squared cost function $c\left(x,\tilde{x}\right)=∥x-\tilde{x}{∥}_{2}^{2}$ was used. The second term was a regularizer based on MMD that compares the prior distribution to the probability distribution of cell features projected to the hidden space. The third term was a constraint for distributional consistency between bulk RNA‐seq and scRNA‐seq data.

### Parameter Settings

The Bis framework consisted of three important components: an encoder, a bottleneck layer, and a decoder, configured with dimensions of [512, 128], 32, and [128, 512], respectively. The optimization was achieved through the implementation of the Adam algorithm, which adopts a learning rate of 1e‐3. The parameters λ and β were meticulously assigned values of 0.01 and 0.5, respectively. In particular, the batch size was set at 4096 for the large dataset with millions of cells and at 512 for other datasets. In addition, the experiments were conducted on an Ubuntu server with an NVIDIA Quadro RTX 6000 GPU and 24GB of memory.

### Comparison with Baseline Methods and Evaluation

The performance of Bis was compared with several state‐of‐the‐art scRNA‐seq data imputation methods, including four single‐cell deep learning‐based imputation methods and five single‐cell model‐based imputation methods. The parameters used for the baseline methods were directly adopted from their original research papers.

- Deep count autoencoder network (DCA, https://github.com/theislab/dca).^[^ ^19^ ^]^ DCA was an encoder‐decoder structure that considered the count distribution, overdispersion, and sparsity of the data using a negative binomial noise model with or without zero inflation.
- Deep neural network‐based imputation algorithm(DeepImpute, https://github.com/lanagarmire/deepimpute).^[^ ^17^ ^]^ DeepImpute was a deep neural network‐based imputation algorithm that leverages dropout layers and loss functions to learn patterns in the data.
- Deep‐learning‐based approach (scScope, https://github.com/AltschulerWu‐Lab/scScope).^[^ ^21^ ^]^ scScope emerges as a scalable deep learning methodology, characterized by its utilization of a recurrent network layer, facilitating iterative imputations.
- Single‐cell variational inference (scVI, https://github.com/YosefLab/scVI).^[^ ^20^ ^]^ scVI serves as a scalable framework for probabilistically representing and analyzing gene expression at the single‐cell level. Utilizing stochastic optimization combined with deep neural networks, scVI efficiently aggregates information from analogous cells and genes and then approximates the distributions inherent to observed expression values while accounting for batch effects and limited sensitivity.
- Adaptively thresholded Low‐Rank Approximation (ALRA, https://github.com/KlugerLab/ALRA).^[^ ^13^ ^]^ ALRA took advantage of the non‐negativity and low‐rank structure of an expression matrix to enable the selective imputation of technical zeros without affecting biologically non‐expressed genes, ensuring that true biological zeros maintain zero expression levels.
- Markov affinity‐based graph imputation of cells (MAGIC, https://github.com/KrishnaswamyLab/MAGIC).^[^ ^16^ ^]^ MAGIC imputed likely gene expression in each cell, revealing the underlying biological structure. MAGIC used signal‐processing principles like those used to clarify blurry and grainy images.
- Negative binomial model (SAVER, https://github.com/mohuangx/SAVER).^[^ ^10^ ^]^ SAVER was a method that took advantage of gene‐to‐gene relationships to recover the true expression level of each gene in each cell. SAVER assumes that the count of each gene in each cell follows a Poisson‐gamma mixture, also known as a negative binomial model.
- Statistical method based on Gamma and Normal distribution (scImpute, https://github.com/Vivianstats/scImpute).^[^ ^11^ ^]^ scImpute was a statistical approach that discerns the dropout probability of each gene within individual cells by fitting a mixture model for each cell type and imputes the (highly probable) dropout values within a cell by borrowing information from the same gene in other similar cells.
- Matrix regularization‐based model (SCRABBLE, https://github.com/tanlabcode/SCRABBLE).^[^ ^15^ ^]^ SCRABBLE was built upon the matrix regularization framework, which incorporated an assumption of low rank for the imputed gene expression matrix.

### Simulated scRNA‐seq Datasets Generation

Splatter^[^ ^24^ ^]^ R package (version v3.16, https://github.com/Oshlack/splatter) was employed for simulating scRNA‐seq datasets. Splatter could capture various features commonly observed in scRNA‐seq data, such as zero inflation, gene‐wise dispersion, and variations in sequencing depths among cells. It allowed the simulation of single populations of cells, populations with multiple cell types, and differentiation paths by offering several parameter controls, including batchCells, nGenes, group.prob, and dropout mid.

In the simulated experiments, batchCells were set to 2000 and nGenes to 500. The group.prob parameter signifies the percentage of each cell type and was configured as (0.2, 0.15, 0.25, 0.1, 0.3) for five cell types. The parameter dropout_mid was used to control the dropout rate in the dataset and was set to (1.5, 2, 3, 4, 5, 6, 7, 8) for eight dropout rates: 26%, 32%, 46%, 53%, 61%, 76%, 86%, and 93%. By configuring these parameters, true data and dropout data were simultaneously using the ‘splatSimulateGroups()’ function in Splatter. Naturally, missing values in the simulated datasets could be identified. For each rate of the simulated dataset, ten replicates were generted.

### Clustering Analysis and Identification of Marker Genes in Cell Clusters

Seurat^[^ ^26^ ^]^ (version 4.3.0, https://github.com/satijalab/seurat) was adopted to perform clustering analysis and identification of marker genes in cell clusters. The function ‘FindClusters()’ in the R package Seurat was used with default parameters to identify clusters of cells. Then, the function ‘FindAllMarkers()’ was used to identify marker genes of each cluster, where the parameters were set to “only.pos=True, min.pct=0.1”. Then, biological marker genes of each cell cluster were selected from the obtained genes with “p_val_adj⩽0.05” and “avg_logFC⩾0.5”. Finally, the expression levels of biological marker genes in different cell clusters were visualized by the function ‘Heatmap()’ of the R package ComplexHeatmap.^[^ ^92^ ^]^

### Batch Effect Analysis

The function scanpy.external.pp.harmony_integrate() in Scanpy^[^ ^40^ ^]^ (version 1.9.1, https://github.com/scverse/scanpy) was used to integrate imputed single‐cell data from multiple experiments with the default parameters. To evaluate the performance of the integrated data, several essential metrics were computed. The Adjusted Rand Index (ARI), Normalized Mutual Information (NMI), Average Silhouette Width (ASW), and modified average silhouette width (b_ASW) of batch (b_ASW) were calculated. These metrics were computed using the dedicated functions ‘scib.metrics.ari()’, ‘scib.metrics.nmi()’, ‘scib.metrics.‐silhouette()’, and ‘scib.metrics.silhouette_batch()’, all of which were available in the Python module scIB.^[^ ^30^ ^]^

### Differential Expression Analysis

In this analysis, DESeq2^[^ ^43^ ^]^ (version 1.40.2, https://bioconductor.org/packages/release/bioc/html/DESeq2.html) was harnessed, specifically utilizing the ‘DESeq()’ function to facilitate the identification of genes displaying differential expression. Subsequently, a list of biologically upregulated and downregulated genes were curated from the results, with the criteria of “padj⩽0.05”, “log2FoldChange⩾1” and “baseMean⩾10”.

### Functional Enrichment

The R package ClusterProfiler^[^ ^44^ ^]^ (version 4.6.2, https://bioconductor.org/packages/release/bioc/html/clusterProfiler.html) was employed to conduct Gene Ontology (GO) and KEGG enrichment analyses on the selected differential expression or marker genes. For GO analysis, the ‘enrichGO()’ function was utilized with specific parameters, including “ont=ALL”, “pAdjustMethod=fdr”, “pvalueCutoff=0.05”, and “qvalueCutoff=0.05”. Similarly, for KEGG analysis, the ‘enrichKEGG()’ function was utilized with parameters set to “pAdjustMethod=fdr”, “pvalueCutoff=0.05”, and “qvalueCutoff=0.05”.

### Trajectory Inference

In Section 2.9 and 2.10, trajectory inference was conducted using Monocle3 (version 1.3.1, https://github.com/cole‐trapnell‐lab/monocle3).^[^ ^60^, ^93^, ^94^ ^]^ Imputed scRNA‐seq data and true labels as input was utilized, loaded through the ‘new_cell_data_set()’ function, and preprocessed the data with the ‘preprocess_cds()’ function. Subsequently, dimensionality reduction was performed using the ‘reduce_dimension()’ function, with the parameter set to “preprocess_method=PCA”. Cell clustering was achieved via the ‘cluster_cells()’ function, with parameters set to “reduction_method=UMAP”, while the principal graph was learned using the ‘learn_graph()’ function, with parameters set to “use_partition=F, close_loop=F, learn_graph_control=NULL, verbose=FALSE”. Finally, the ‘plot_cells()’ function was employed for visualizing the trajectory inference. Simultaneously, Kendall's rank correlation score was calculated using the ‘cor()’ function from the R package stat, with parameters set to “method=kendall, use=complete.obs”. Additionally, the Pseudo‐temporal Ordering(POS) was computed using the ‘orderscore()’ function from the R package TSCAN.^[^ ^64^ ^]^

### TF‐Gene Network

The construction of a TF‐gene network was initiated with the selected 354 differentially expressed genes using the NetworkAnalyst platform,^[^ ^54^ ^]^ leveraging data from the JASPAR^[^ ^55^ ^]^ database. Following this, a filtering process was applied to the TF‐gene network, removing nodes with a ‘degree’ value of less than 20 to improve the overall quality of TFs within the network. Finally, Cytoscape was used to visualize the resulting network.

### Evaluation Metrics

The evaluation metric Pearson correlation coefficient (PCC) was adopted to measure the performance of all methods on simulated and down‐sampling experiments. For the removal of batch effect, adjusted rand index (ARI), normalized mutual information (NMI), and the average silhouette width (ASW) across cell types and batches were introduced for a more comprehensive analysis. For clustering analysis, in addition to the previously mentioned ARI and NMI, the Jaccard index was also utilized to evaluate the clustering accuracy. In the comparative analysis of differential expression, accuracy (ACC) was utilized, the area under the curve (AUC), and the F1 score to measure the accuracy of differential gene identification.

*Pearson Correlation Coefficient*: The PCC quantifies the linear correlation between the imputed expression values and the true values of genes and cells and it is defined as:

(7) $$\text{PCC}=\frac{\sum_{i=1}^{n}\left(x_{i}-\bar{x}\right)\left(y_{i}-\bar{y}\right)}{\sqrt{\sum_{i=1}^{n}{\left(x_{i}-\bar{x}\right)}^{2}\sum_{i=1}^{n}{\left(y_{i}-\bar{y}\right)}^{2}}},$$

where $x_{i}$ and $y_{i}$ are the imputed and true expression values of the same gene or cell, and $\bar{x}$ and $\bar{y}$ are their respective means. The result always has a value between ‐1 and 1.

*NMI*: The Normalized Mutual Information (NMI) was used to assess the overlap between two cluster sets. The degree of overlap was adjusted by scaling using the average of the entropy terms associated with the cell type and cluster labels. As a result, NMI scores of 0 or 1 indicate uncorrelated clustering or a perfect match, respectively. It is defined as:

(8) $$\text{NMI}=\frac{2\sum_{k=1}^{K}\sum_{j=1}^{J}n_{kj}\log\left(\frac{N\cdot n_{kj}}{n_{k}\cdot n_{j}}\right)}{\sum_{k=1}^{K}n_{k}\log\left(\frac{N\cdot n_{k}}{n_{k}}\right)+\sum_{j=1}^{J}n_{j}\log\left(\frac{N\cdot n_{j}}{n_{j}}\right)}$$

where $n_{\mathrm{kj}}$ is the number of cells assigned to both cluster k and cluster j, $n_{k}$ is the number of cells assigned to cluster k, $n_{j}$ is the number of cells assigned to cluster j, and N is is the total number of cells.

*ARI*: The Rand Index was a measure that evaluates the overlap between two cluster sets. It took into account both correct clustering overlaps and correct disagreements between the two clusterings. An Adjusted Rand Index (ARI) score of 0 or 1 corresponds to random labeling or a perfect match, respectively. It is defined as:

(9) ARI=∑i,jnij2−∑iai2∑jbj2/N212∑iai2+∑jbj2−∑iai2∑jbj2/N2

where $n_{\mathrm{ij}}$ is the number of cells assigned to both cluster i and cluster j, $a_{i}$ is the number of cells assigned to cluster i, $b_{j}$ is the number of cells assigned to cluster j, and N is the total number of cells.

*Jaccard Index*: The Jaccard index quantified the similarity of the pairwise overlaps between the predicted clusters using imputed scRNA‐seq data and the true cell types. The Jaccard index ranges from 0 to 1, where a score of 1 indicates a perfect match or complete overlap, while a score of 0 indicates no common elements between the sets being compared. It is defined as:

(10) $$\text{Jaccard Index}=\frac{\sum_{i}\sum_{j}n_{ij}}{\sum_{i}\left(\sum_{j}n_{ij}\right)+\sum_{j}\left(\sum_{i}n_{ij}\right)-\sum_{i}\sum_{j}n_{ij}}$$

where $n_{\mathrm{ij}}$ is the number of cells assigned to both cluster i and cluster j.

*ASW*: The silhouette width quantified the balance between the within‐cluster distances of a cell and the between‐cluster distances of that cell to the nearest cluster.^[^ ^95^ ^]^ By averaging the silhouette widths of a set of cells, the Average Silhouette Width (ASW) was obtained, which falls within the range of ‐1 to 1. The ASW was widely utilized to assess cluster separation, where a value of 1 indicates dense and well‐separated clusters. On the other hand, a value of 0 or ‐1 corresponds to overlapping clusters (caused by similar within‐ and between‐cluster variability) or significant misclassification (due to stronger within‐cluster variability than between‐cluster variability), respectively. To provide a more comprehensive evaluation of the batch effect removal results, the two ASW calculation methods proposed in reference were employed,^[^ ^30^ ^]^ denoted as ASW and b_ASW. The first one was the classical definition of ASW to determine the silhouette of the cell labels (cell‐type ASW) and the other was a modified approach to measure batch mixing. Both metrics were computed on the provided PCA of imputed expression matrices. The ASW was calculated based on the cell identity labels and scaled to a value between 0 and 1 using the following equation:

(11) $$\text{cell type ASW}=({\mathrm{ASW}}_{C}+1)/2$$

where C denotes the set of all cell identity labels.

The batch mixing score b_ASW was a measure used to evaluate the degree of batch mixing within cell clusters. It was derived by computing the absolute silhouette widths on batch labels for each cell and scaling them to a range between 0 and 1 and is defined as:

(12) $$b\_\mathrm{ASW}=\frac{1}{\left|M\right|}\sum_{j\in M}\;\text{batch}\;{\mathrm{ASW}}_{j}$$

where M is the set of unique cell labels, batch ASW_*j*_ is to assess batch mixing within each cell label independently. It is defined as:

(13) $$\text{batch}\;{\text{ASW}}_{j}=\frac{1}{\left|C_{j}\right|}\sum_{i\in C_{j}}1-s_{\text{batch}\;}\left(i\right)$$

where $C_{j}$ is the set of cells with the cell label j and $|C_{j}|$ denotes the number of cells in that set, and $s_{\text{batch}}\left(i\right)$ is the absolute silhouette width based on the batch labels assigned to each cell, denoted as:

(14) $$s_{\text{batch}\;}\left(i\right)=\left|s\left(i\right)\right|$$

*Accuary*: Accuracy in the context of single‐cell RNA sequencing (scRNA‐seq) differential expression analysis refers to the ability to accurately identify genes that were differentially expressed between different conditions or cell types. It measured the proportion of correctly classified genes as either differentially expressed or non‐differentially expressed. The accuracy could be calculated (Equation (17)) by determining the true positive (TP), true negative (TN), false positive (FP), and false negative (FN) gene classifications. These classifications were based on whether a gene was correctly identified as differentially expressed (TP and TN) or incorrectly identified (FP and FN). A higher accuracy score indicated better performance in correctly identifying differentially expressed genes in scRNA‐seq data.

(15) $$\;\text{Accuracy}\;=\frac{\;\text{Number of correct predictions}\;}{\;\text{Total number of predictions}\;}$$

*F1 score*: In differential expression analysis of scRNA‐seq data, precision represented the proportion of correctly identified differentially expressed genes of all the predicted differentially expressed genes. It measured the accuracy of the positive predictions. Recall, on the other hand, measures the proportion of correctly identified differentially expressed genes out of all the truly differentially expressed genes in the dataset. It quantified the ability to capture all the positive instances. The F1 score was the harmonic mean of precision and recall, providing a single value that represents the balance between the two metrics. It ranges from 0 to 1, where a higher F1 score indicates better performance in accurately identifying differentially expressed genes. The F1 score is calculated using the following formula:

(16) $$F1=\frac{2\times \text{precision}\times \text{recall}}{\text{precision}+\text{recall}}=\frac{2\times \text{TP}}{2\times \text{TP}+\text{FP}+\text{FN}}$$

*Area Under the ROC Curve (AUC)*: The ROC curve plots the true positive rate (TPR) against the false positive rate (FPR) across various threshold determinations. The AUC score, capturing the expanse beneath the ROC curve, oscillates between 0 and 1. A higher AUC score indicated better performance in differentiating between differentially expressed genes and non‐differentially expressed genes. The AUC measured the ability of the differential expression analysis method to correctly rank differentially expressed genes higher than non‐differentially expressed genes across various threshold settings. It provided a comprehensive evaluation of the discriminatory power of the method.

## Conflict of Interest

The authors declare no conflict of interest.

## Author Contributions

X.L. conceived and supervised the project. Y.S. developed and implemented the algorithms under the guidance of X.L., and Y.S. wrote the manuscript. Y.S. conducted the experiments. Y.S., Z.Y., and Y.Y. completed the figures and manuscript. Y.S., X.L., and K.C.W. revised the manuscript. All authors approved the manuscript.

## Supporting information

Supporting Information

## Data Availability

The data that support the findings of this study are openly available in Zenodo at https://doi.org/10.5281/zenodo.8395467. These data were derived from the following resources available in the public domain: [GSE109774], https://www.ncbi.nlm.nih.gov/geo/query/acc.cgi?acc=GSE109774; [GSE132040], https://www.ncbi.nlm.nih.gov/geo/query/acc.cgi?acc=GSE132040; [GSE108097], https://www.ncbi.nlm.nih.gov/geo/query/acc.cgi?acc=GSE108097; [GSE29184], https://www.ncbi.nlm.nih.gov/geo/query/acc.cgi?acc=GSE29184; [GSE184652], https://www.ncbi.nlm.nih.gov/geo/query/acc.cgi?acc=GSE184652; [GSE199437], https://www.ncbi.nlm.nih.gov/geo/query/acc.cgi?acc=GSE199437; [GSE81076], https://www.ncbi.nlm.nih.gov/geo/query/acc.cgi?acc=GSE81076; [GSE84133], https://www.ncbi.nlm.nih.gov/geo/query/acc.cgi?acc=GSE84133; [GSE81608], https://www.ncbi.nlm.nih.gov/geo/query/acc.cgi?acc=GSE81608; [GSE85241], https://www.ncbi.nlm.nih.gov/geo/query/acc.cgi?acc=GSE85241.
