# Supplementary material for: Distribution‐Agnostic Deep Learning Enables Accurate Single‐Cell Data Recovery and Transcriptional Regulation Interpretation
Source: Adv Sci (Weinh). 2024 Feb 21;11(16):2307280. doi: 10.1002/advs.202307280 (PMC11040354; doi:10.1002/advs.202307280)
Supplement: Supplementary file 1 — Supporting Information [file ADVS-11-2307280-s001.pdf]

ADVANCED SCIENCE
Open Access

Supporting Information

for *Adv. Sci.*, DOI 10.1002/advs.202307280

Distribution-Agnostic Deep Learning Enables Accurate Single-Cell Data Recovery and Transcriptional Regulation Interpretation

*Yanchi Su, Zhuohan Yu, Yuning Yang, Ka-Chun Wong and Xiangtao Li**

Supplementary Information "Distribution-agnostic Deep Learning Enables Accurate Single-Cell Data Recovery and Transcriptional Regulation Interpretation"

*Yanchi Su Zhuohan Yu Yuning Yang Ka-Chun Wong Xiangtao Li**

Y.C. Su, Z.H. Yu, X.T. Li
School of Artificial Intelligence
Jilin University
Jilin, China
Email Address: lixt314@jlu.edu.cn
Y.N. Yang
Donnelly Centre for Cellular and Biomolecular Research
University of Toronto
Toronto, ON, Canada
K.C. Wong
Department of Computer Science
City University of Hong Kong
Hong Kong SAR

1 Bis is the most accurate imputation method on scRNA-seq data

Table 1: Overview of downsampling and clustering dataset.

	Downsampling experiments				Clustering experiments			
**Datasets**	**Liver**	**Lung**	**Heart**	**Marrow**	**Spleen**	**Kidney**	**FetalLiver**	**Lung**
**Cell**	714	1716	4365	5037	2417	5423	3730	9116
**Gene**	23433	23433	23433	23433	17545	19430	16488	20765
**Cell Type**	5	7	7	22	7	28	20	32

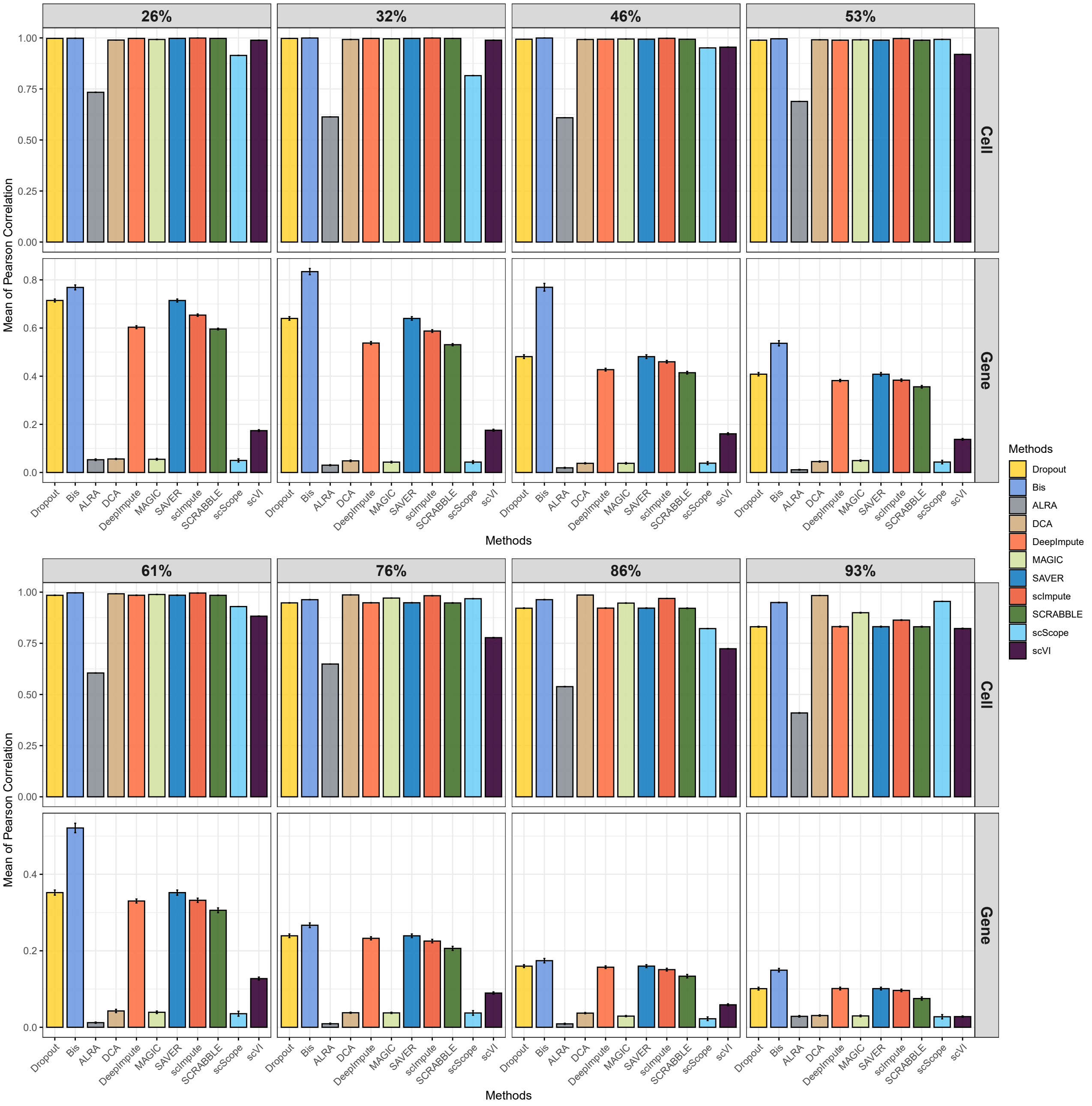

Figure 1: Performance evaluation of different methods using eight simulated datasets with specific dropout rates. Each barplot represents the statistical mean and variance of the Pearson correlation coefficient of cells and genes.

2 Bis improves cell-type identification and generates better visualization on scRNA-seq data

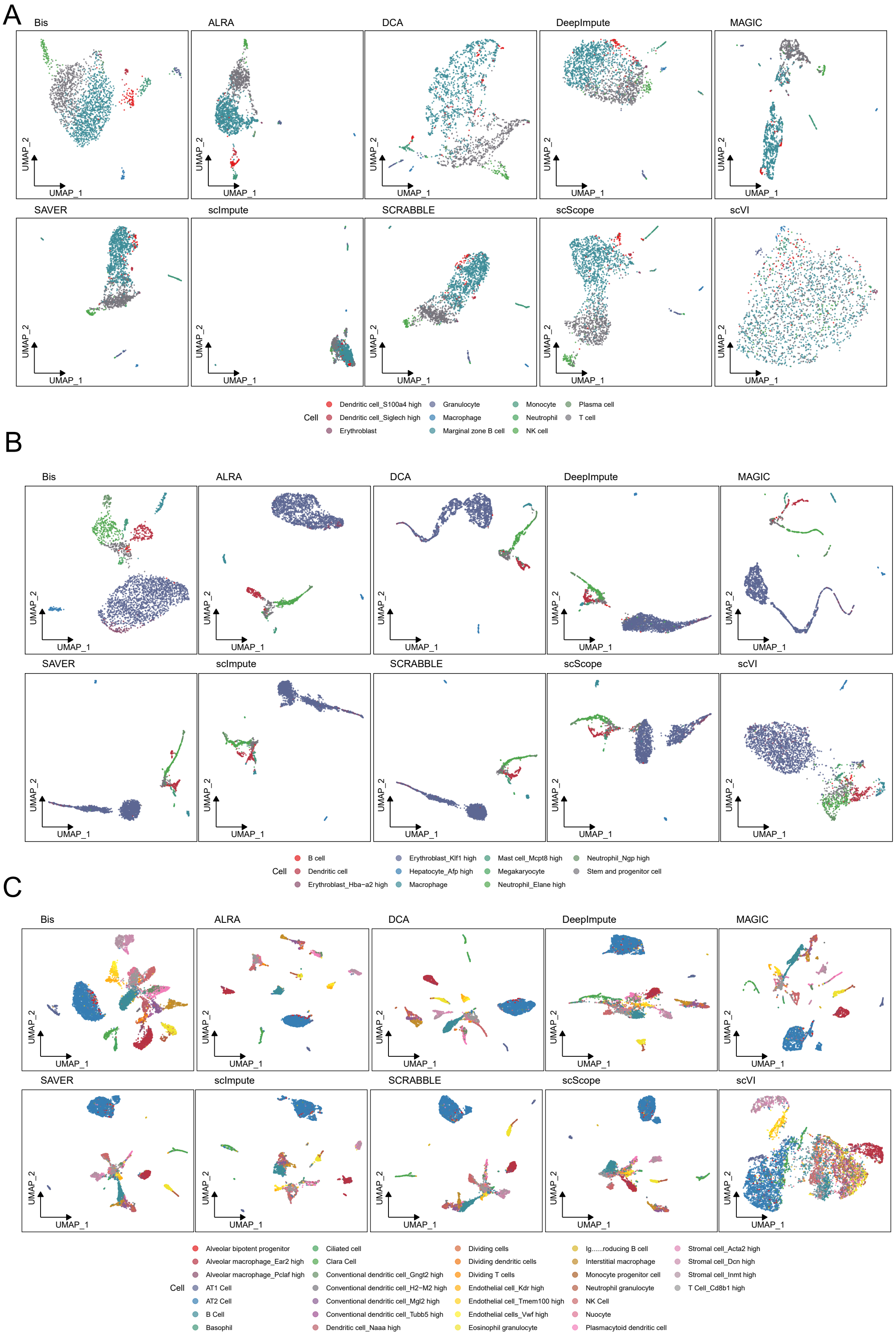

Figure 2: **(A)** depicts plots of UMAP visualization of Spleen data after imputation with various methods. **(B)** depicts plots of UMAP visualization of FetalLiver data after imputation with various methods. **(C)** depicts plots of UMAP visualization of Lung data after imputation with various methods.

3 Ablation study and hyperparameter selection

Table 2: Overview of different parameter cases for four datasets.

	layer	λ	β		layer	λ	β		layer	λ	β		layer	λ	β
**case1**	8	0.01	0.1	**case26**	16	0.01	0.1	**case51**	32	0.01	0.1	**case76**	64	0.01	0.1
**case2**	8	0.01	0.3	**case27**	16	0.01	0.3	**case52**	32	0.01	0.3	**case77**	64	0.01	0.3
**case3**	8	0.01	0.5	**case28**	16	0.01	0.5	**case53**	32	0.01	0.5	**case78**	64	0.01	0.5
**case4**	8	0.01	0.7	**case29**	16	0.01	0.7	**case54**	32	0.01	0.7	**case79**	64	0.01	0.7
**case5**	8	0.01	0.9	**case30**	16	0.01	0.9	**case55**	32	0.01	0.9	**case80**	64	0.01	0.9
**case6**	8	0.03	0.1	**case31**	16	0.03	0.1	**case56**	32	0.03	0.1	**case81**	64	0.03	0.1
**case7**	8	0.03	0.3	**case32**	16	0.03	0.3	**case57**	32	0.03	0.3	**case82**	64	0.03	0.3
**case8**	8	0.03	0.5	**case33**	16	0.03	0.5	**case58**	32	0.03	0.5	**case83**	64	0.03	0.5
**case9**	8	0.03	0.7	**case34**	16	0.03	0.7	**case59**	32	0.03	0.7	**case84**	64	0.03	0.7
**case10**	8	0.03	0.9	**case35**	16	0.03	0.9	**case60**	32	0.03	0.9	**case85**	64	0.03	0.9
**case11**	8	0.05	0.1	**case36**	16	0.05	0.1	**case61**	32	0.05	0.1	**case86**	64	0.05	0.1
**case12**	8	0.05	0.3	**case37**	16	0.05	0.3	**case62**	32	0.05	0.3	**case87**	64	0.05	0.3
**case13**	8	0.05	0.5	**case38**	16	0.05	0.5	**case63**	32	0.05	0.5	**case88**	64	0.05	0.5
**case14**	8	0.05	0.7	**case39**	16	0.05	0.7	**case64**	32	0.05	0.7	**case89**	64	0.05	0.7
**case15**	8	0.05	0.9	**case40**	16	0.05	0.9	**case65**	32	0.05	0.9	**case90**	64	0.05	0.9
**case16**	8	0.07	0.1	**case41**	16	0.07	0.1	**case66**	32	0.07	0.1	**case91**	64	0.07	0.1
**case17**	8	0.07	0.3	**case42**	16	0.07	0.3	**case67**	32	0.07	0.3	**case92**	64	0.07	0.3
**case18**	8	0.07	0.5	**case43**	16	0.07	0.5	**case68**	32	0.07	0.5	**case93**	64	0.07	0.5
**case19**	8	0.07	0.7	**case44**	16	0.07	0.7	**case69**	32	0.07	0.7	**case94**	64	0.07	0.7
**case20**	8	0.07	0.9	**case45**	16	0.07	0.9	**case70**	32	0.07	0.9	**case95**	64	0.07	0.9
**case21**	8	0.09	0.1	**case46**	16	0.09	0.1	**case71**	32	0.09	0.1	**case96**	64	0.09	0.1
**case22**	8	0.09	0.3	**case47**	16	0.09	0.3	**case72**	32	0.09	0.3	**case97**	64	0.09	0.3
**case23**	8	0.09	0.5	**case48**	16	0.09	0.5	**case73**	32	0.09	0.5	**case98**	64	0.09	0.5
**case24**	8	0.09	0.7	**case49**	16	0.09	0.7	**case74**	32	0.09	0.7	**case99**	64	0.09	0.7
**case25**	8	0.09	0.9	**case50**	16	0.09	0.9	**case75**	32	0.09	0.9	**case100**	64	0.09	0.9

Table 3: Results of clustering with different parameters on four datasets.

	**ARI**	**NMI**	**Jaccard**		**ARI**	**NMI**	**Jaccard**
**case1**	0.3732	0.5804	0.3637	**case51**	0.5747	0.6367	0.5075
**case2**	0.4940	0.6735	0.4705	**case52**	0.6568	0.6703	0.5980
**case3**	0.5729	0.6629	0.5065	**case53**	0.6258	0.7157	0.5932
**case4**	0.6671	0.7560	0.6333	**case54**	0.6417	0.6723	0.5788
**case5**	0.5954	0.7364	0.5715	**case55**	0.5358	0.6672	0.4648
**case6**	0.3514	0.5770	0.3360	**case56**	0.4123	0.6266	0.3968
**case7**	0.4673	0.6814	0.4613	**case57**	0.4840	0.6853	0.4779
**case8**	0.5112	0.6538	0.5036	**case58**	0.6220	0.6944	0.5493
**case9**	0.5778	0.6950	0.5419	**case59**	0.6107	0.7054	0.5673
**case10**	0.6715	0.7226	0.6257	**case60**	0.6983	0.7548	0.6633
**case11**	0.3748	0.6351	0.3628	**case61**	0.4528	0.6266	0.4095
**case12**	0.3611	0.6254	0.3557	**case62**	0.3870	0.6397	0.3761
**case13**	0.3383	0.5448	0.3622	**case63**	0.6138	0.6945	0.5410
**case14**	0.5030	0.6466	0.5133	**case64**	0.5344	0.7310	0.5097
**case15**	0.6452	0.7132	0.6175	**case65**	0.7166	0.7710	0.6677
**case16**	0.3568	0.6034	0.3374	**case66**	0.3995	0.5981	0.3729
**case17**	0.4589	0.6471	0.4602	**case67**	0.3479	0.5680	0.3357
**case18**	0.4066	0.6234	0.4130	**case68**	0.6431	0.6996	0.5738
**case19**	0.3884	0.5752	0.3798	**case69**	0.5765	0.7300	0.5704
**case20**	0.4982	0.7011	0.4942	**case70**	0.5728	0.6894	0.5499
**case21**	0.4639	0.6439	0.4197	**case71**	0.3361	0.6009	0.3194
**case22**	0.3923	0.6631	0.3711	**case72**	0.3632	0.6151	0.3524
**case23**	0.4613	0.6733	0.4440	**case73**	0.6431	0.6996	0.5738
**case24**	0.4810	0.7096	0.4709	**case74**	0.5337	0.7044	0.5300
**case25**	0.5848	0.6995	0.5544	**case75**	0.6743	0.7737	0.6357
**case26**	0.3802	0.5957	0.3677	**case76**	0.3303	0.5359	0.3380
**case27**	0.4752	0.6761	0.4626	**case77**	0.6091	0.6945	0.5799
**case28**	0.5775	0.6681	0.5055	**case78**	0.6147	0.6802	0.5477
**case29**	0.6333	0.7235	0.5950	**case79**	0.6102	0.7283	0.5974
**case30**	0.6141	0.7193	0.5774	**case80**	0.4770	0.6564	0.4498
**case31**	0.3423	0.5953	0.3209	**case81**	0.2993	0.5721	0.2963
**case32**	0.5148	0.7004	0.4878	**case82**	0.5171	0.7340	0.4849
**case33**	0.5087	0.6584	0.5223	**case83**	0.6054	0.7419	0.6079
**case34**	0.5722	0.7042	0.5418	**case84**	0.5767	0.7203	0.5606
**case35**	0.6197	0.7189	0.5946	**case85**	0.5901	0.6692	0.5729
**case36**	0.3833	0.6273	0.3601	**case86**	0.4627	0.6260	0.4240
**case37**	0.4133	0.6677	0.4199	**case87**	0.3676	0.6398	0.3676
**case38**	0.5208	0.6557	0.5015	**case88**	0.6250	0.7463	0.5918
**case39**	0.5249	0.6833	0.4824	**case89**	0.4781	0.6733	0.4578
**case40**	0.5403	0.6596	0.5133	**case90**	0.6102	0.7512	0.5819
**case41**	0.4179	0.6332	0.3858	**case91**	0.3111	0.5823	0.3010
**case42**	0.4367	0.6850	0.4352	**case92**	0.3672	0.5727	0.3791
**case43**	0.4623	0.6636	0.4611	**case93**	0.4480	0.6301	0.4534
**case44**	0.5517	0.6705	0.5297	**case94**	0.4880	0.7048	0.4733
**case45**	0.6650	0.7325	0.6352	**case95**	0.4728	0.6838	0.4517
**case46**	0.4010	0.6369	0.3683	**case96**	0.3909	0.6086	0.3580
**case47**	0.3819	0.6348	0.3603	**case97**	0.4495	0.6610	0.4241
**case48**	0.3953	0.5877	0.3845	**case98**	0.5082	0.6621	0.4797
**case49**	0.4207	0.6558	0.4270	**case99**	0.5662	0.7259	0.5348
**case50**	0.6241	0.7336	0.5875	**case100**	0.5217	0.7268	0.5086

4 Bis can facilitate the removal of batch effects in human pancreatic data from different scRNA-seq protocols

Table 4: Overview of cellular identities of pancreas dataset.

celltype	celseq	celseq2	fluidigmc1	inDrop1	inDrop2	inDrop3	inDrop4	smarter	smartseq2
**acinar**	228	274	21	110	3	843	2	0	188
**activated_stellate**	19	90	16	51	81	100	52	0	55
**alpha**	191	843	239	236	676	1130	284	886	1008
**beta**	161	445	258	872	371	787	495	472	308
**delta**	50	203	25	214	125	161	101	49	127
**ductal**	327	258	36	120	301	376	280	0	444
**endothelial**	5	21	14	130	23	92	7	0	21
**epsilon**	1	4	1	13	2	2	1	0	8
**gamma**	18	110	18	70	86	36	63	85	213
**macrophage**	1	15	1	14	17	14	10	0	7
**mast**	1	6	3	8	9	7	1	0	7
**quiescent_stellate**	1	12	1	92	22	54	5	0	6
**schwann**	1	4	5	5	6	1	1	0	2
**t_cell**	0	0	0	2	2	2	1	0	0
**totals**	1004	2285	638	1937	1724	3605	1303	1492	2394

Table 5: Overview of cellular identities (proportions) of pancreas dataset.

cell type	**celseq**	**celseq2**	**fluidigmc1**	**inDrop1**	**inDrop2**	**inDrop3**	**inDrop4**	**smarter**	**smartseq2**
**acinar**	22.71	11.99	3.29	5.68	0.17	23.38	0.15	0.00	7.85
**activated_stellate**	1.89	3.94	2.51	2.63	4.70	2.77	3.99	0.00	2.30
**alpha**	19.02	36.89	37.46	12.18	39.21	31.35	21.80	59.38	42.11
**beta**	16.04	19.47	40.44	45.02	21.52	21.83	37.99	31.64	12.87
**delta**	4.98	8.88	3.92	11.05	7.25	4.47	7.75	3.28	5.30
**ductal**	32.57	11.29	5.64	6.20	17.46	10.43	21.49	0.00	18.55
**endothelial**	0.50	0.92	2.19	6.71	1.33	2.55	0.54	0.00	0.88
**epsilon**	0.10	0.18	0.16	0.67	0.12	0.06	0.08	0.00	0.33
**gamma**	1.79	4.81	2.82	3.61	4.99	1.00	4.83	5.70	8.90
**macrophage**	0.10	0.66	0.16	0.72	0.99	0.39	0.77	0.00	0.29
**mast**	0.10	0.26	0.47	0.41	0.52	0.19	0.08	0.00	0.29
**quiescent_stellate**	0.10	0.53	0.16	4.75	1.28	1.50	0.38	0.00	0.25
**schwann**	0.10	0.18	0.78	0.26	0.35	0.03	0.08	0.00	0.08
**t_cell**	0.00	0.00	0.00	0.10	0.12	0.06	0.08	0.00	0.00
**Total (percent)**	100	100	100	100	100	100	100	100	100

5 Bis improves differential gene expression analysis

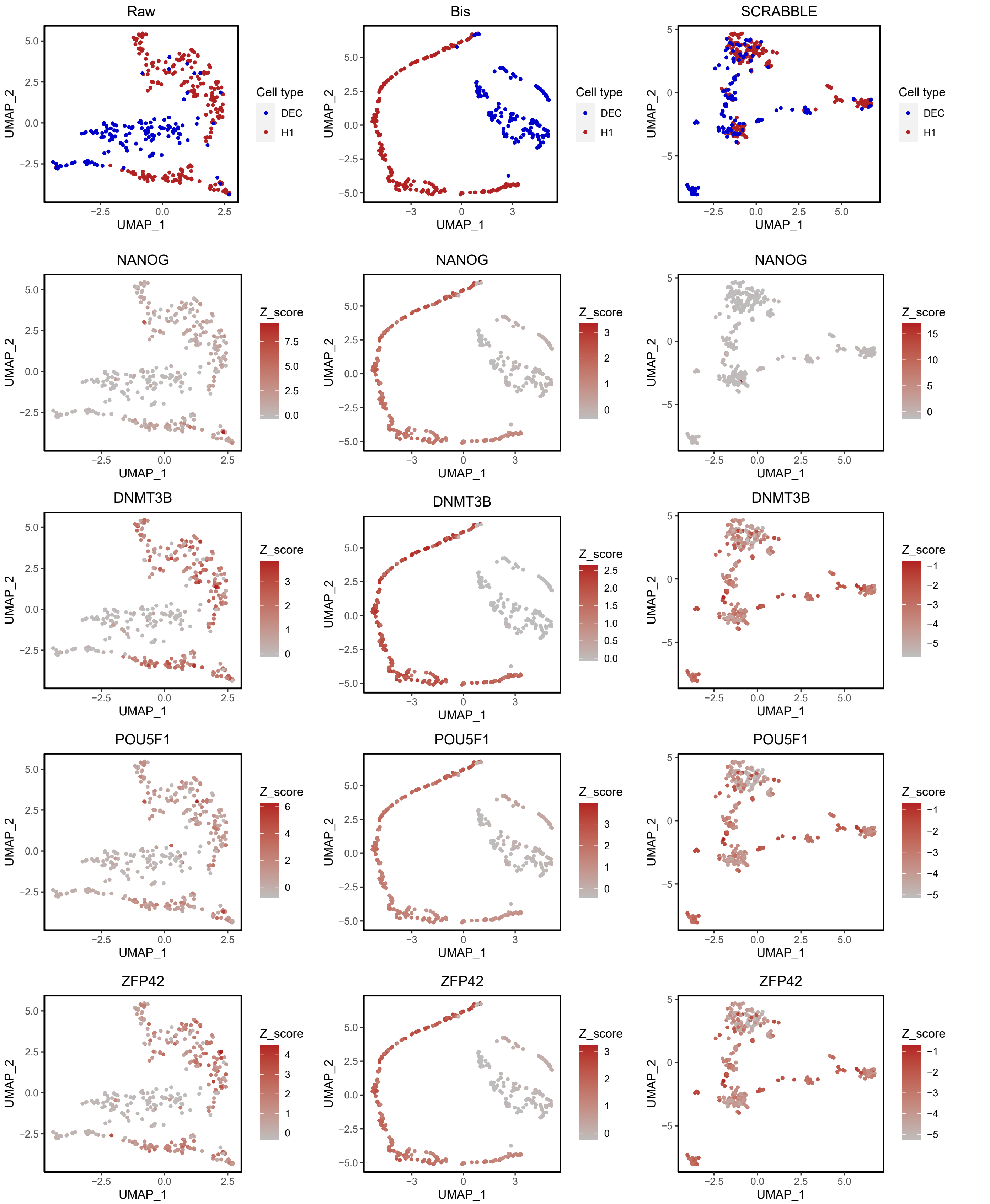

Figure 3: The UMAP plots of single cells are presented, with the expression of NANOG, DNMT3B, POU5F1, and ZFP42 (marker gene for H1 cells).

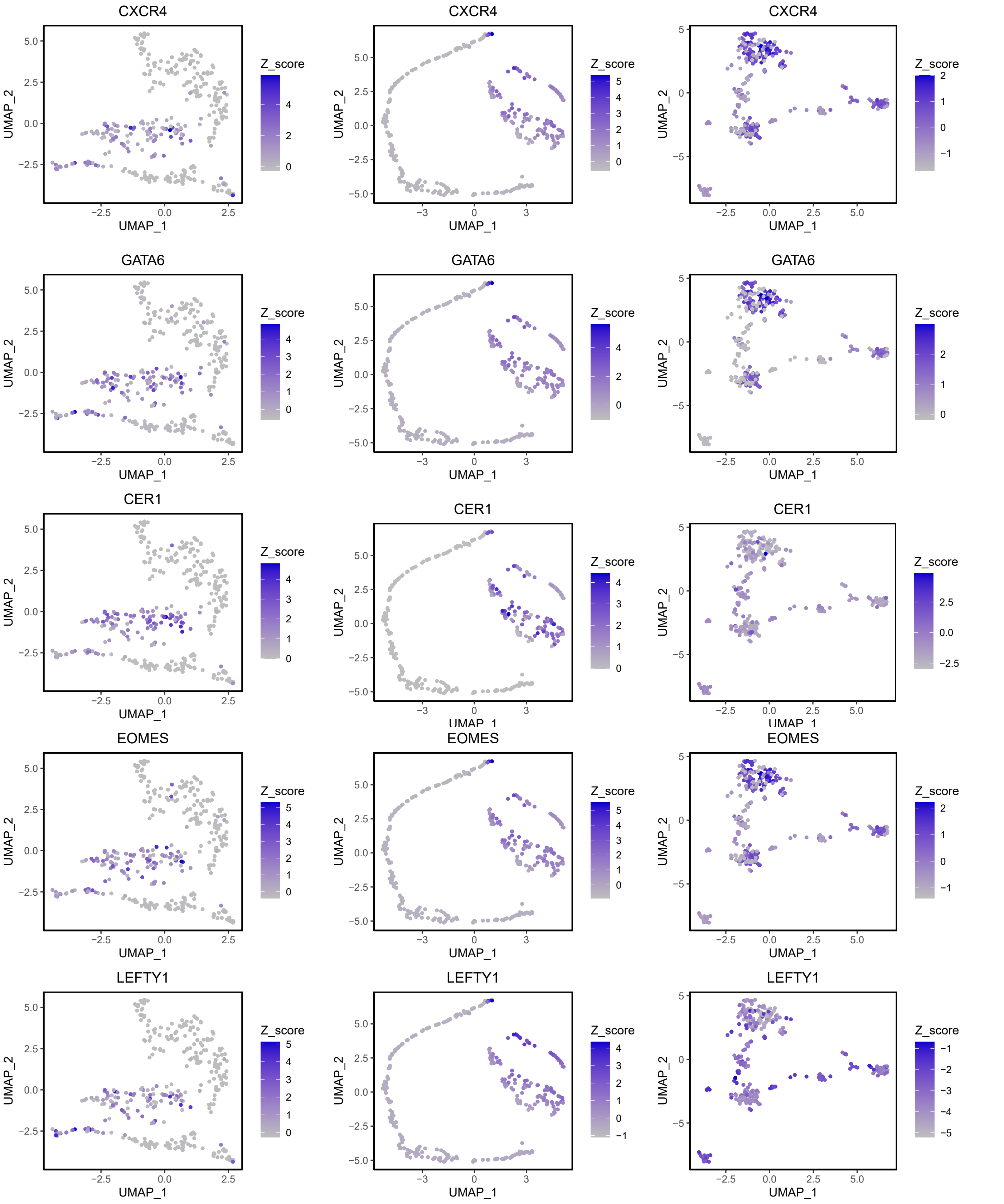

Figure 4: The UMAP plots of single cells are presented, with the expression of CXCR4, GATA6, CER1, EOMES, and LEFTY1 (marker gene for H1 cells).

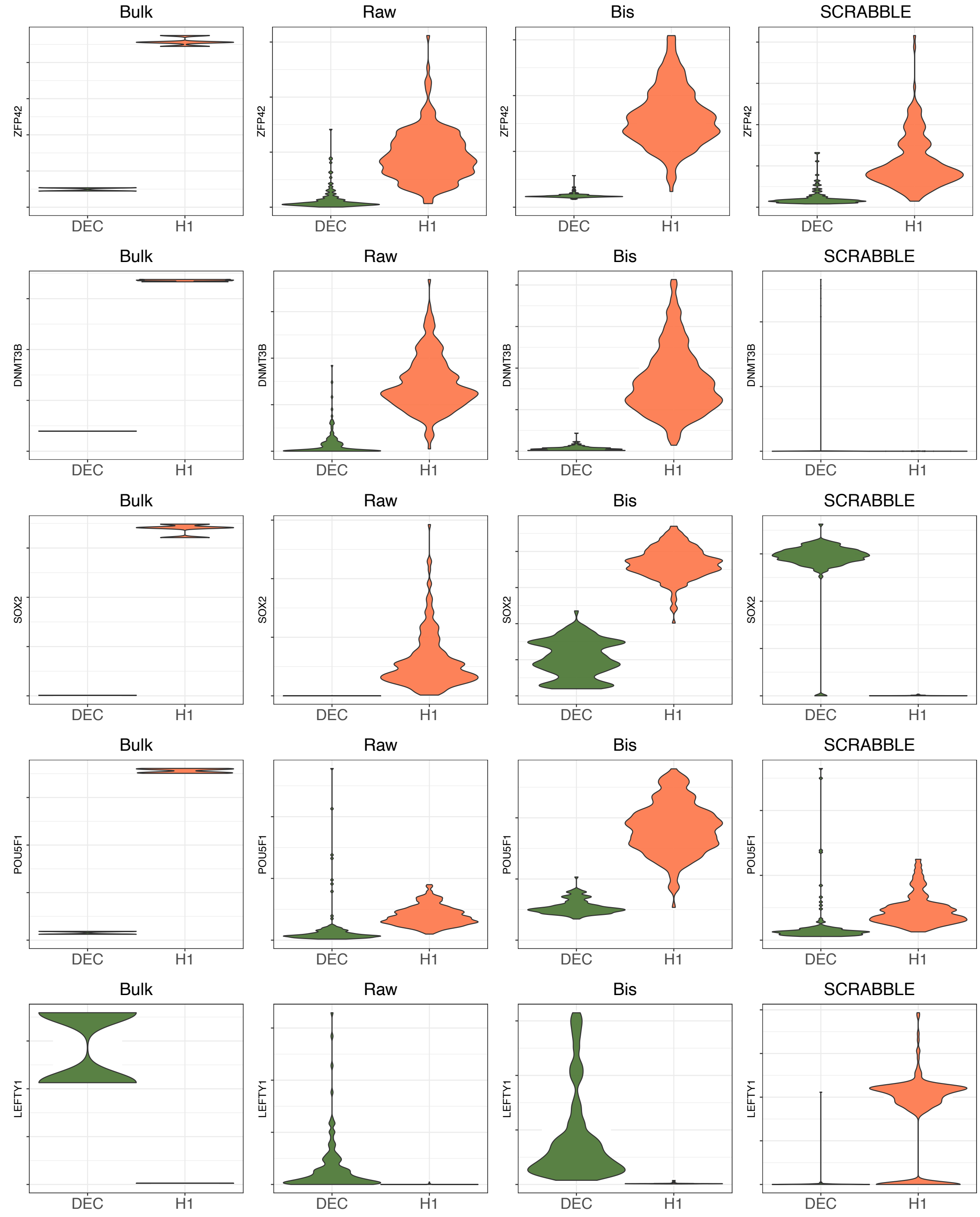

Figure 5: The expression profiles of the five selected signature genes (ZFP42, DNMT3B, SOX2, POU5F1, and LEFTY1) for H1 and DEC cells are shown.

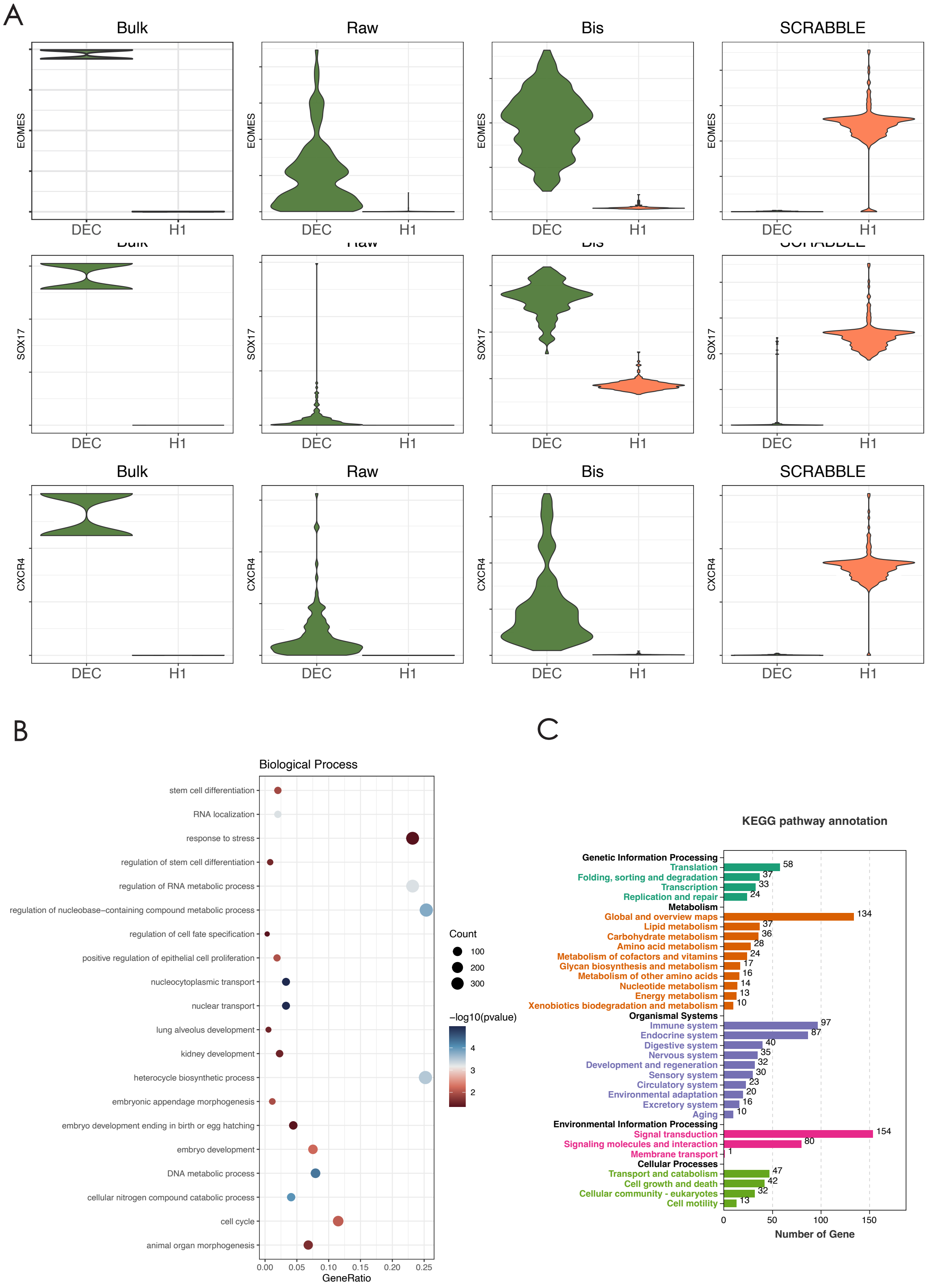

Figure 6: **(A)** The expression profiles of one of the six selected signature genes (EOMES, SOX17, and CXCR4) for DEC cells are shown. **(B)** The top 20 categories of biological process. **(C)** KEGG classification. The Y-axis lists the pathways: Metabolism, Genetic Information Processing, Environmental Information Processing, Cellular Processes, and Organismal Systems.

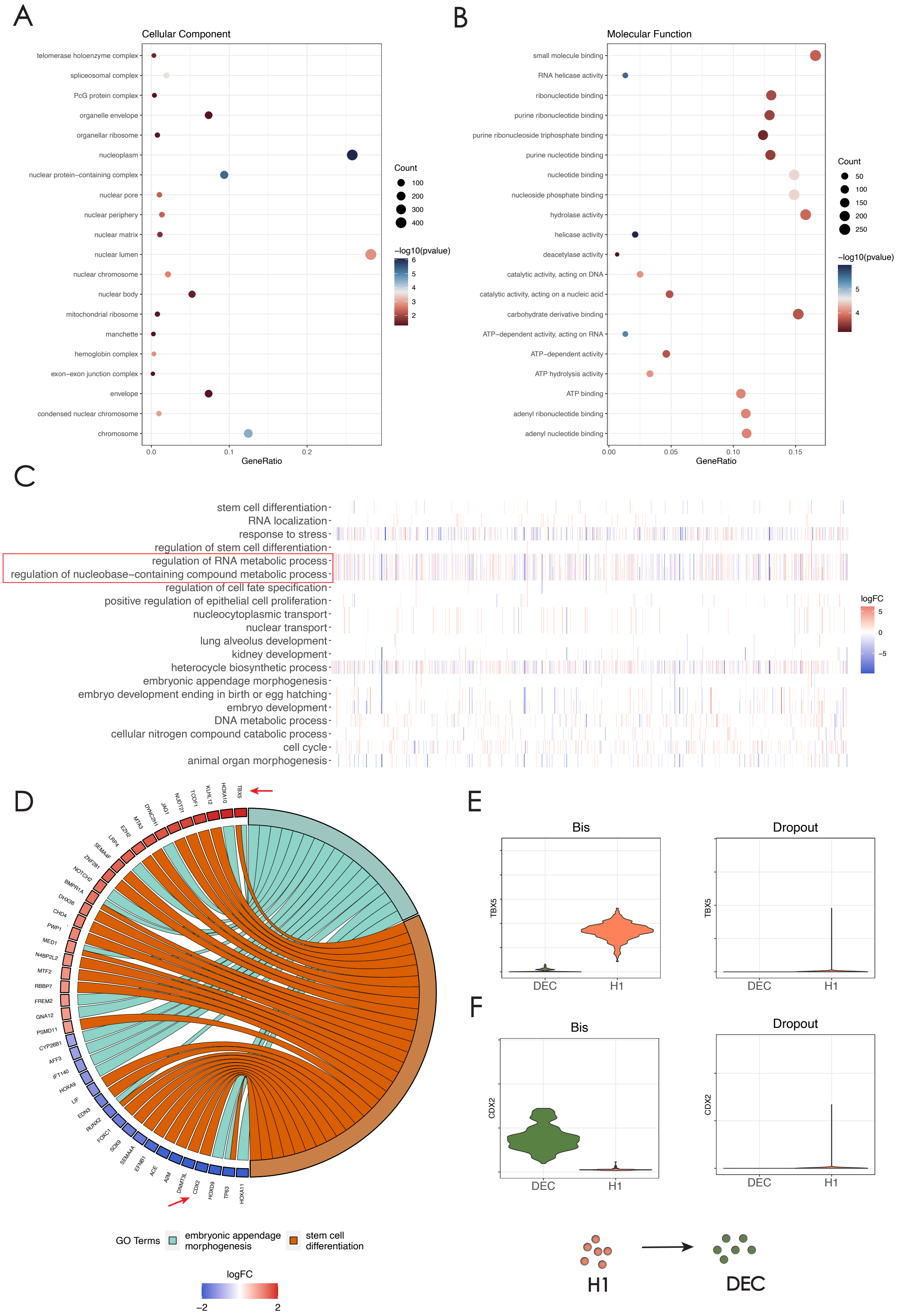

Figure 7: **(A)** Bubble plots displaying the 20 enriched GO terms of cellular component. **(B)** Bubble plots displaying the 20 enriched GO terms of molecular function. **(C)** Heatmap plot shows the relationship between the DEGs and GO terms of biological process. **(D)** Chord plot showed the relationship between the DEGs and GO terms of biological process. **(E)** The expression profiles of TBX5 for DEC and H1 cells are shown. **(F)** The expression profiles of CDX2 for DEC and H1 cells are shown.

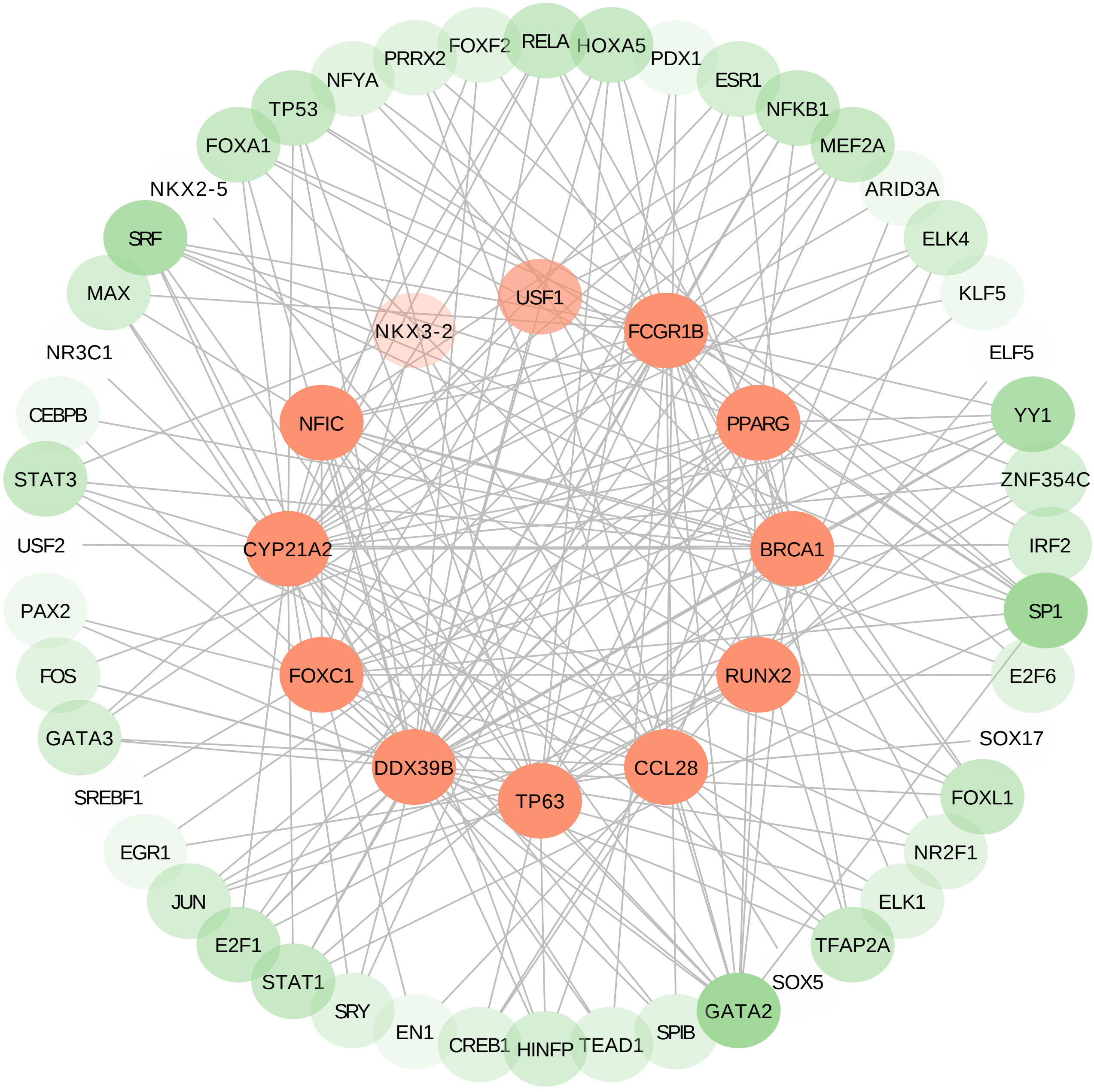

Figure 8: The TFs-DEGs interaction network. The red nodes are TFs, and the blue nodes represent genes. The degree refers to the number of edges that are connected to the specified node in the graph. The greater the degree of the node, the greater the scale.

6 Bis enhances the inference of cellular trajectories

Table 6: Overview of different parameter cases for UMAP.

**UMAP**					
**(n, m, d), where n=n_neighbors, m=metric, d=min_dist**					
**case1**	(10, euclidean, 0.001)	**case13**	(15, cosine, 0.001)	**case25**	(20, correlation, 0.001)
**case2**	(10, euclidean, 0.01)	**case14**	(15, cosine, 0.01)	**case26**	(20, correlation, 0.01)
**case3**	(10, euclidean, 0.1)	**case15**	(15, cosine, 0.1)	**case27**	(20, correlation, 0.1)
**case4**	(10, cosine, 0.001)	**case16**	(15, correlation, 0.001)	**case28**	(25, euclidean, 0.001)
**case5**	(10, cosine, 0.01)	**case17**	(15, correlation, 0.01)	**case29**	(25, euclidean, 0.01)
**case6**	(10, cosine, 0.1)	**case18**	(15, correlation, 0.1)	**case30**	(25, euclidean, 0.01)
**case7**	(10, correlation, 0.001)	**case19**	(20, euclidean, 0.001)	**case31**	(25, cosine, 0.001)
**case8**	(10, correlation, 0.01)	**case20**	(20, euclidean, 0.01)	**case32**	(25, cosine, 0.01)
**case9**	(10, correlation, 0.1)	**case21**	(20, euclidean, 0.1)	**case33**	(25, cosine, 0.1)
**case10**	(15, euclidean, 0.001)	**case22**	(20, cosine, 0.001)	**case34**	(25, correlation, 0.001)
**case11**	(15, euclidean, 0.01)	**case23**	(20, cosine, 0.01)	**case35**	(25, correlation, 0.01)
**case12**	(15, euclidean, 0.1)	**case24**	(20, cosine, 0.1)	**case36**	(25, correlation, 0.1)

Table 7: Results of Kendall's rank correlation scores with different parameters for UMAP.

	Dropout	Bis	ALRA	DCA	DeepImpute	MAGIC	SAVER	scImpute	SCRABBLE	scScope	scVI
case1	0.4704	0.7881	0.3056	0.2937	0.5615	0.3249	0.0989	0.3249	0.3099	0.3757	0.0902
case2	0.4704	0.7881	0.3056	0.2937	0.5615	0.3249	0.0989	0.3249	0.3099	0.3757	0.0902
case3	0.4704	0.7881	0.3056	0.2937	0.5615	0.3249	0.0989	0.3249	0.3099	0.3757	0.0902
case4	0.4704	0.7881	0.3056	0.2937	0.5615	0.3249	0.0989	0.3249	0.3099	0.3757	0.0902
case5	0.4704	0.7881	0.3056	0.2937	0.5615	0.3249	0.0989	0.3249	0.3099	0.3757	0.0902
case6	0.4704	0.7881	0.3056	0.2937	0.5615	0.3249	0.0989	0.3249	0.3099	0.3757	0.0902
case7	0.4704	0.7881	0.3056	0.2937	0.5615	0.3249	0.0989	0.3249	0.3099	0.3757	0.0902
case8	0.4704	0.7881	0.3056	0.2937	0.5615	0.3249	0.0989	0.3249	0.3099	0.3757	0.0902
case9	0.4704	0.7881	0.3056	0.2937	0.5615	0.3249	0.0989	0.3249	0.3099	0.3757	0.0902
case10	0.4704	0.7881	0.3052	0.3476	0.5615	0.4361	0.0989	0.4361	0.3099	0.3757	0.1357
case11	0.4704	0.7881	0.3052	0.3476	0.5615	0.4361	0.0989	0.4361	0.3099	0.3757	0.1357
case12	0.4704	0.7881	0.3052	0.3476	0.5615	0.4361	0.0989	0.4361	0.3099	0.3757	0.1357
case13	0.4704	0.7881	0.3052	0.3476	0.5615	0.4361	0.0989	0.4361	0.3099	0.3757	0.1357
case14	0.4704	0.7881	0.3052	0.3476	0.5615	0.4361	0.0989	0.4361	0.3099	0.3757	0.1357
case15	0.4704	0.7881	0.3052	0.3476	0.5615	0.4361	0.0989	0.4361	0.3099	0.3757	0.1357
case16	0.4704	0.7881	0.3052	0.3476	0.5615	0.4361	0.0989	0.4361	0.3099	0.3757	0.1357
case17	0.4704	0.7881	0.3052	0.3476	0.5615	0.4361	0.0989	0.4361	0.3099	0.3757	0.1357
case18	0.4704	0.7881	0.3052	0.3476	0.5615	0.4361	0.0989	0.4361	0.3099	0.3757	0.1357
case19	0.4704	0.8118	0.5835	0.3476	0.5615	0.4361	0.5751	0.4361	0.4310	0.3757	0.2362
case20	0.4704	0.8118	0.5835	0.3476	0.5615	0.4361	0.5751	0.4361	0.4310	0.3757	0.2362
case21	0.4704	0.8118	0.5835	0.3476	0.5615	0.4361	0.5751	0.4361	0.4310	0.3757	0.2362
case22	0.4704	0.8118	0.5835	0.3476	0.5615	0.4361	0.5751	0.4361	0.4310	0.3757	0.2362
case23	0.4704	0.8118	0.5835	0.3476	0.5615	0.4361	0.5751	0.4361	0.4310	0.3757	0.2362
case24	0.4704	0.8118	0.5835	0.3476	0.5615	0.4361	0.5751	0.4361	0.4310	0.3757	0.2362
case25	0.4704	0.8118	0.5835	0.3476	0.5615	0.4361	0.5751	0.4361	0.4310	0.3757	0.2362
case26	0.4704	0.8118	0.5835	0.3476	0.5615	0.4361	0.5751	0.4361	0.4310	0.3757	0.2362
case27	0.4704	0.8118	0.5835	0.3476	0.5615	0.4361	0.5751	0.4361	0.4310	0.3757	0.2362
case28	0.4704	0.8118	0.5835	0.3476	0.5615	0.4361	0.5751	0.4361	0.4310	0.3757	0.2362
case29	0.4704	0.8118	0.5835	0.3476	0.5615	0.4361	0.5751	0.4361	0.4310	0.3757	0.2362
case30	0.4704	0.8118	0.5835	0.3476	0.5615	0.4361	0.5751	0.4361	0.4310	0.3757	0.2362
case31	0.4704	0.8118	0.5835	0.3476	0.5615	0.4361	0.5751	0.4361	0.4310	0.3757	0.2362
case32	0.4704	0.8118	0.5835	0.3476	0.5615	0.4361	0.5751	0.4361	0.4310	0.3757	0.2362
case33	0.4704	0.8118	0.5835	0.3476	0.5615	0.4361	0.5751	0.4361	0.4310	0.3757	0.2362
case34	0.4704	0.8118	0.5835	0.3476	0.5615	0.4361	0.5751	0.4361	0.4310	0.3757	0.2362
case35	0.4704	0.8118	0.5835	0.3476	0.5615	0.4361	0.5751	0.4361	0.4310	0.3757	0.2362
case36	0.4704	0.8118	0.5835	0.3476	0.5615	0.4361	0.5751	0.4361	0.4310	0.3757	0.2362
average	0.4704	0.7999	0.4444	0.3341	0.5615	0.4083	0.3370	0.4083	0.3705	0.3757	0.1746

Table 8: Results of Pseudo-temporal Ordering (POS) with different parameters for UMAP.

	**Dropout**	**Bis**	**ALRA**	**DCA**	**DeepImpute**	**MAGIC**	**SAVER**	**scImpute**	**SCRABBLE**	**scScope**	**scVI**
**case1**	0.5867	0.9409	0.3523	0.3323	0.7617	0.3545	0.1275	0.3545	0.4039	0.5221	0.1164
**case2**	0.5867	0.9409	0.3523	0.3323	0.7617	0.3545	0.1275	0.3545	0.4039	0.5221	0.1164
**case3**	0.5867	0.9409	0.3523	0.3323	0.7617	0.3545	0.1275	0.3545	0.4039	0.5221	0.1164
**case4**	0.5867	0.9409	0.3523	0.3323	0.7617	0.3545	0.1275	0.3545	0.4039	0.5221	0.1164
**case5**	0.5867	0.9409	0.3523	0.3323	0.7617	0.3545	0.1275	0.3545	0.4039	0.5221	0.1164
**case6**	0.5867	0.9409	0.3523	0.3323	0.7617	0.3545	0.1275	0.3545	0.4039	0.5221	0.1164
**case7**	0.5867	0.9409	0.3523	0.3323	0.7617	0.3545	0.1275	0.3545	0.4039	0.5221	0.1164
**case8**	0.5867	0.9409	0.3523	0.3323	0.7617	0.3545	0.1275	0.3545	0.4039	0.5221	0.1164
**case9**	0.5867	0.9409	0.3523	0.3323	0.7617	0.3545	0.1275	0.3545	0.4039	0.5221	0.1164
**case10**	0.5867	0.9409	0.3558	0.3598	0.7617	0.644	0.1275	0.644	0.4039	0.5221	0.1379
**case11**	0.5867	0.9409	0.3558	0.3598	0.7617	0.644	0.1275	0.644	0.4039	0.5221	0.1379
**case12**	0.5867	0.9409	0.3558	0.3598	0.7617	0.644	0.1275	0.644	0.4039	0.5221	0.1379
**case13**	0.5867	0.9409	0.3558	0.3598	0.7617	0.644	0.1275	0.644	0.4039	0.5221	0.1379
**case14**	0.5867	0.9409	0.3558	0.3598	0.7617	0.644	0.1275	0.644	0.4039	0.5221	0.1379
**case15**	0.5867	0.9409	0.3558	0.3598	0.7617	0.644	0.1275	0.644	0.4039	0.5221	0.1379
**case16**	0.5867	0.9409	0.3558	0.3598	0.7617	0.644	0.1275	0.644	0.4039	0.5221	0.1379
**case17**	0.5867	0.9409	0.3558	0.3598	0.7617	0.644	0.1275	0.644	0.4039	0.5221	0.1379
**case18**	0.5867	0.9409	0.3558	0.3598	0.7617	0.644	0.1275	0.644	0.4039	0.5221	0.1379
**case19**	0.5867	0.9517	0.7537	0.3598	0.7617	0.644	0.764	0.644	0.5417	0.5221	0.1855
**case20**	0.5867	0.9517	0.7537	0.3598	0.7617	0.644	0.764	0.644	0.5417	0.5221	0.1855
**case21**	0.5867	0.9517	0.7537	0.3598	0.7617	0.644	0.764	0.644	0.5417	0.5221	0.1855
**case22**	0.5867	0.9517	0.7537	0.3598	0.7617	0.644	0.764	0.644	0.5417	0.5221	0.1855
**case23**	0.5867	0.9517	0.7537	0.3598	0.7617	0.644	0.764	0.644	0.5417	0.5221	0.1855
**case24**	0.5867	0.9517	0.7537	0.3598	0.7617	0.644	0.764	0.644	0.5417	0.5221	0.1855
**case25**	0.5867	0.9517	0.7537	0.3598	0.7617	0.644	0.764	0.644	0.5417	0.5221	0.1855
**case26**	0.5867	0.9517	0.7537	0.3598	0.7617	0.644	0.764	0.644	0.5417	0.5221	0.1855
**case27**	0.5867	0.9517	0.7537	0.3598	0.7617	0.644	0.764	0.644	0.5417	0.5221	0.1855
**case28**	0.5867	0.9517	0.7537	0.3598	0.7617	0.644	0.764	0.644	0.5417	0.5221	0.1855
**case29**	0.5867	0.9517	0.7537	0.3598	0.7617	0.644	0.764	0.644	0.5417	0.5221	0.1855
**case30**	0.5867	0.9517	0.7537	0.3598	0.7617	0.644	0.764	0.644	0.5417	0.5221	0.1855
**case31**	0.5867	0.9517	0.7537	0.3598	0.7617	0.644	0.764	0.644	0.5417	0.5221	0.1855
**case32**	0.5867	0.9517	0.7537	0.3598	0.7617	0.644	0.764	0.644	0.5417	0.5221	0.1855
**case33**	0.5867	0.9517	0.7537	0.3598	0.7617	0.644	0.764	0.644	0.5417	0.5221	0.1855
**case34**	0.5867	0.9517	0.7537	0.3598	0.7617	0.644	0.764	0.644	0.5417	0.5221	0.1855
**case35**	0.5867	0.9517	0.7537	0.3598	0.7617	0.644	0.764	0.644	0.5417	0.5221	0.1855
**case36**	0.5867	0.9517	0.7537	0.3598	0.7617	0.644	0.764	0.644	0.5417	0.5221	0.1855
**average**	0.5867	0.9463	0.5538	0.3529	0.7617	0.5716	0.4458	0.5716	0.4728	0.5221	0.1563

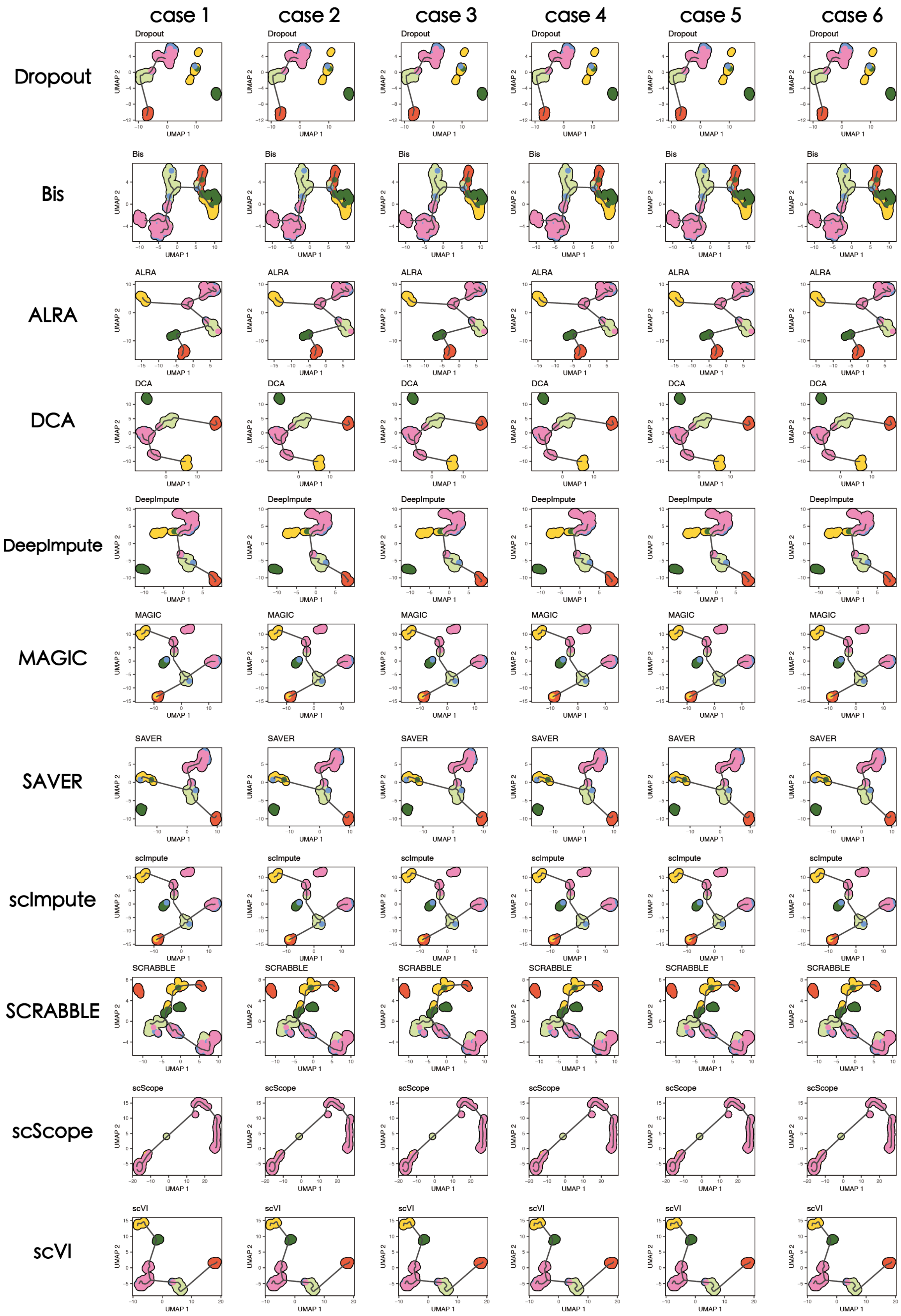

Figure 9: The cases 1-6 of trajectories reconstructed by Monocle3 from the imputed scRNA-seq data using all methods.

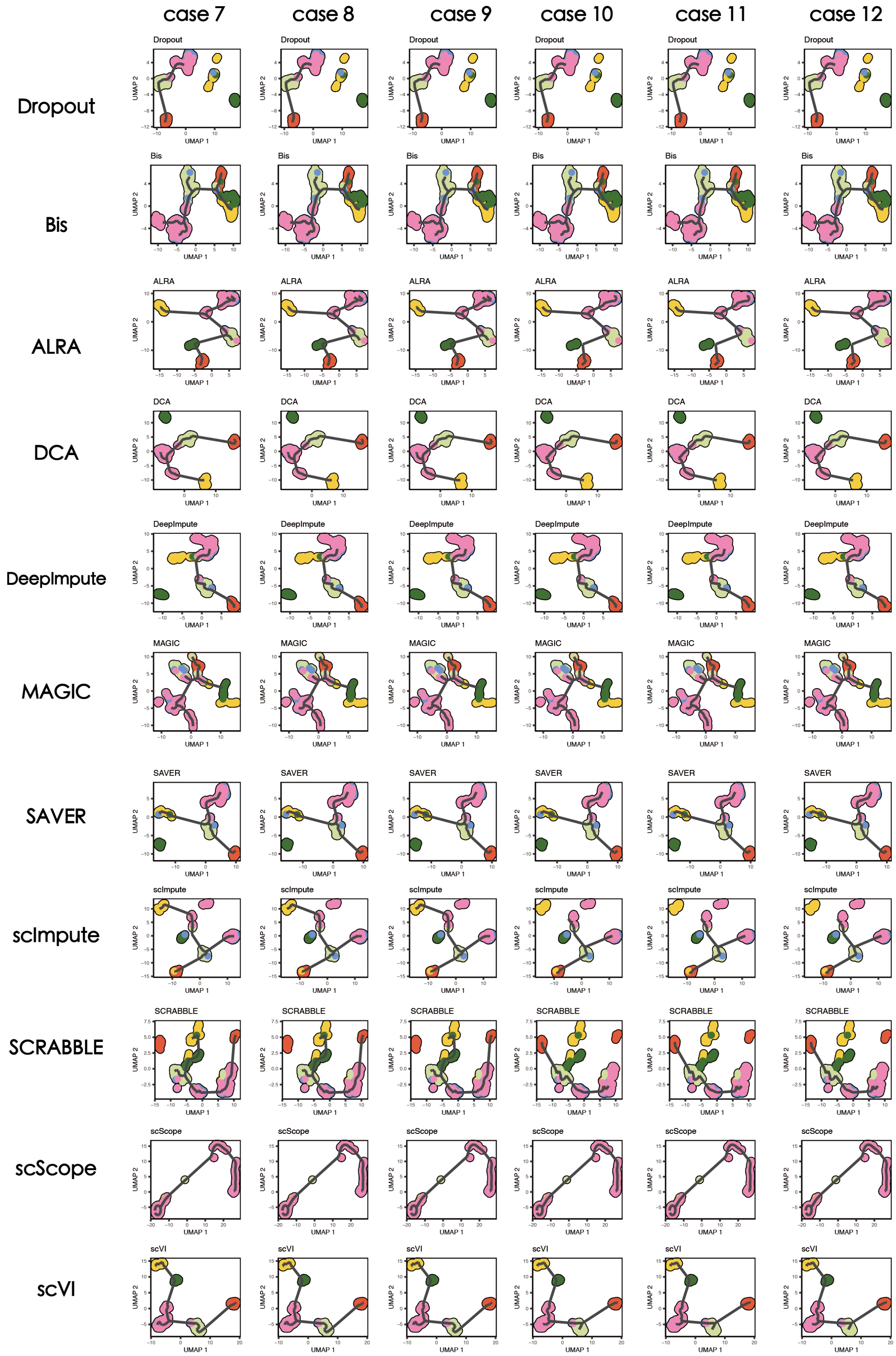

Figure 10: The cases 7-12 of trajectories reconstructed by Monocle3 from the imputed scRNA-seq data using all methods.

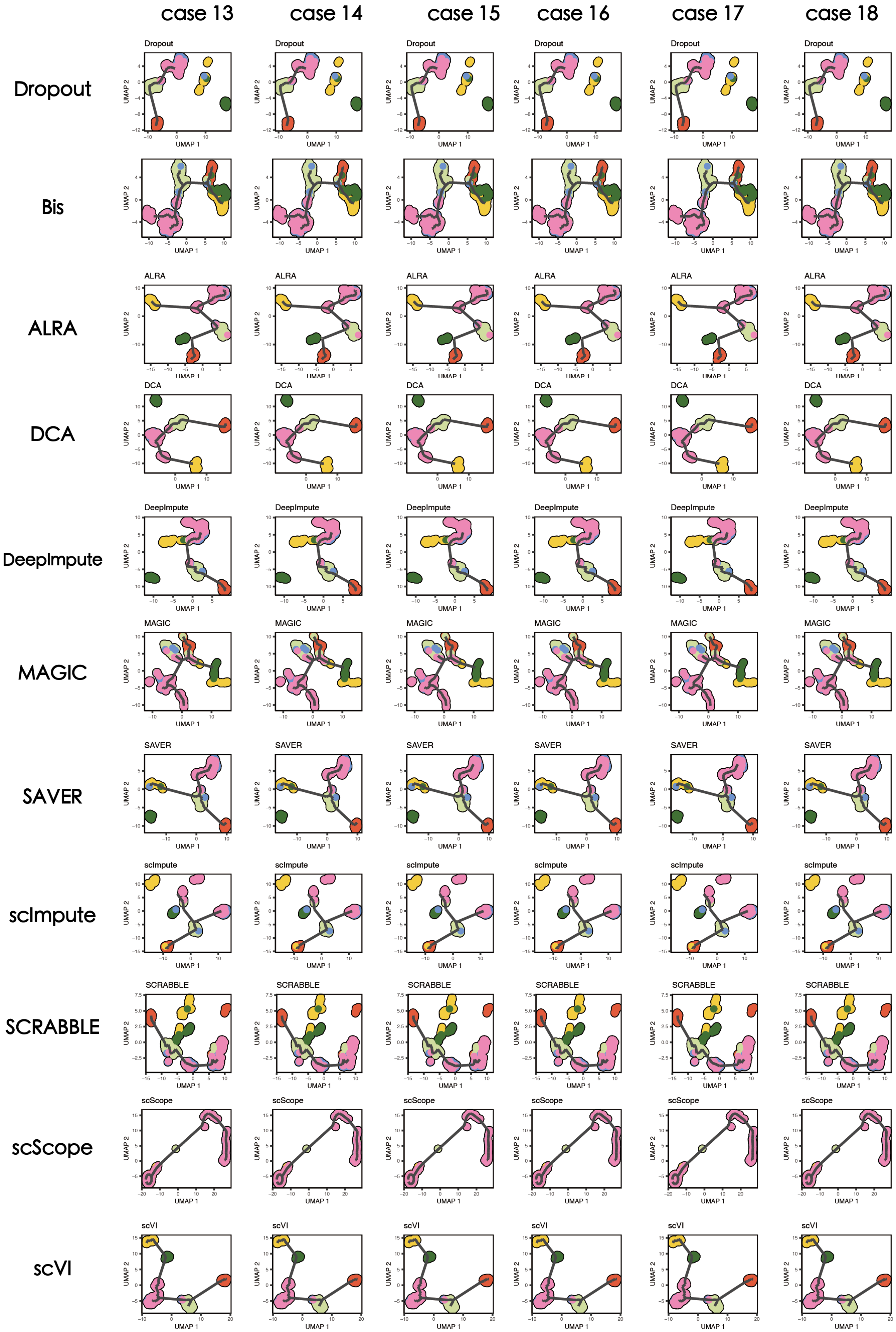

Figure 11: The cases 13-18 of trajectories reconstructed by Monocle3 from the imputed scRNA-seq data using all methods.

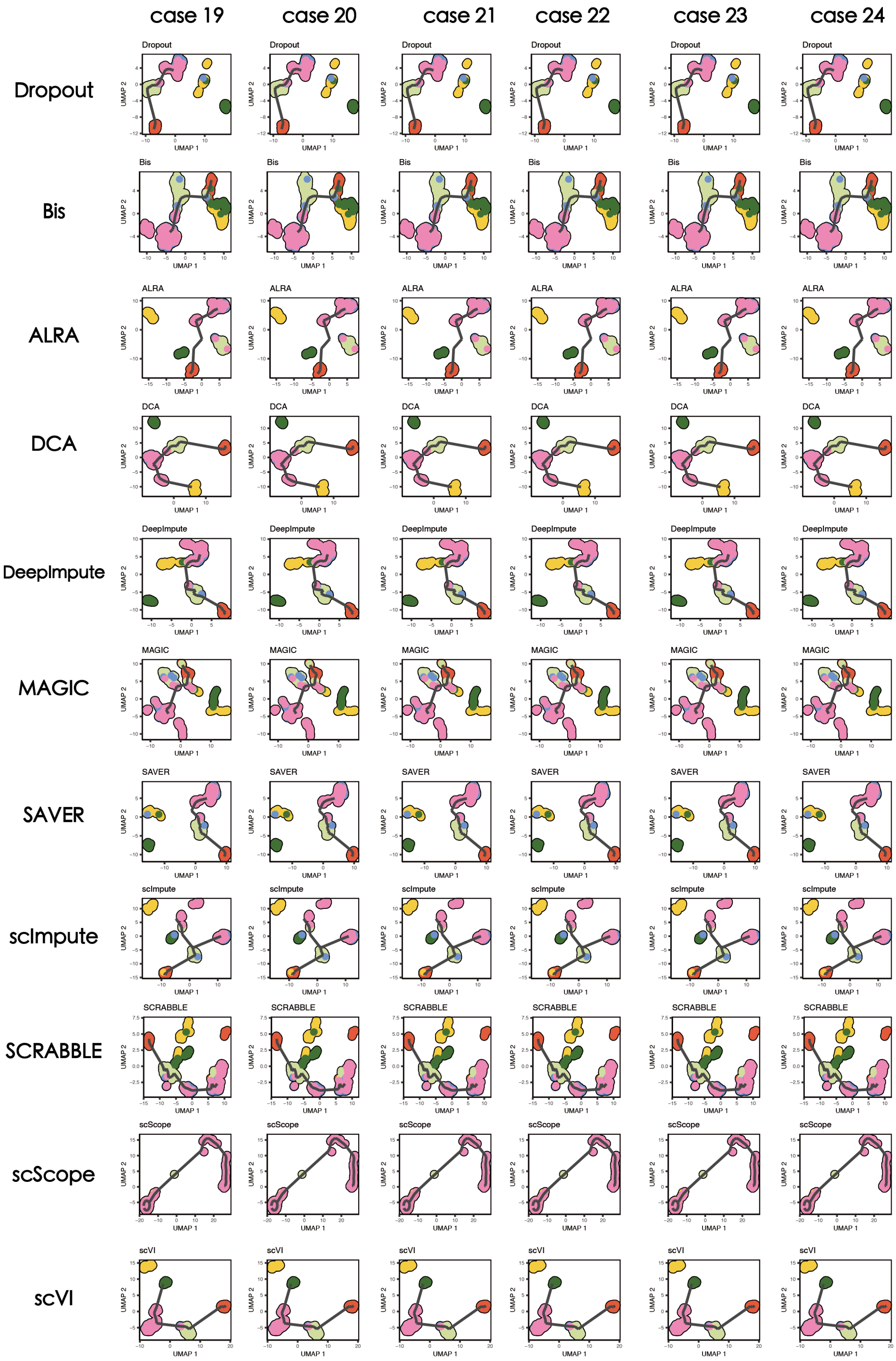

Figure 12: The cases 19-24 of trajectories reconstructed by Monocle3 from the imputed scRNA-seq data using all methods.

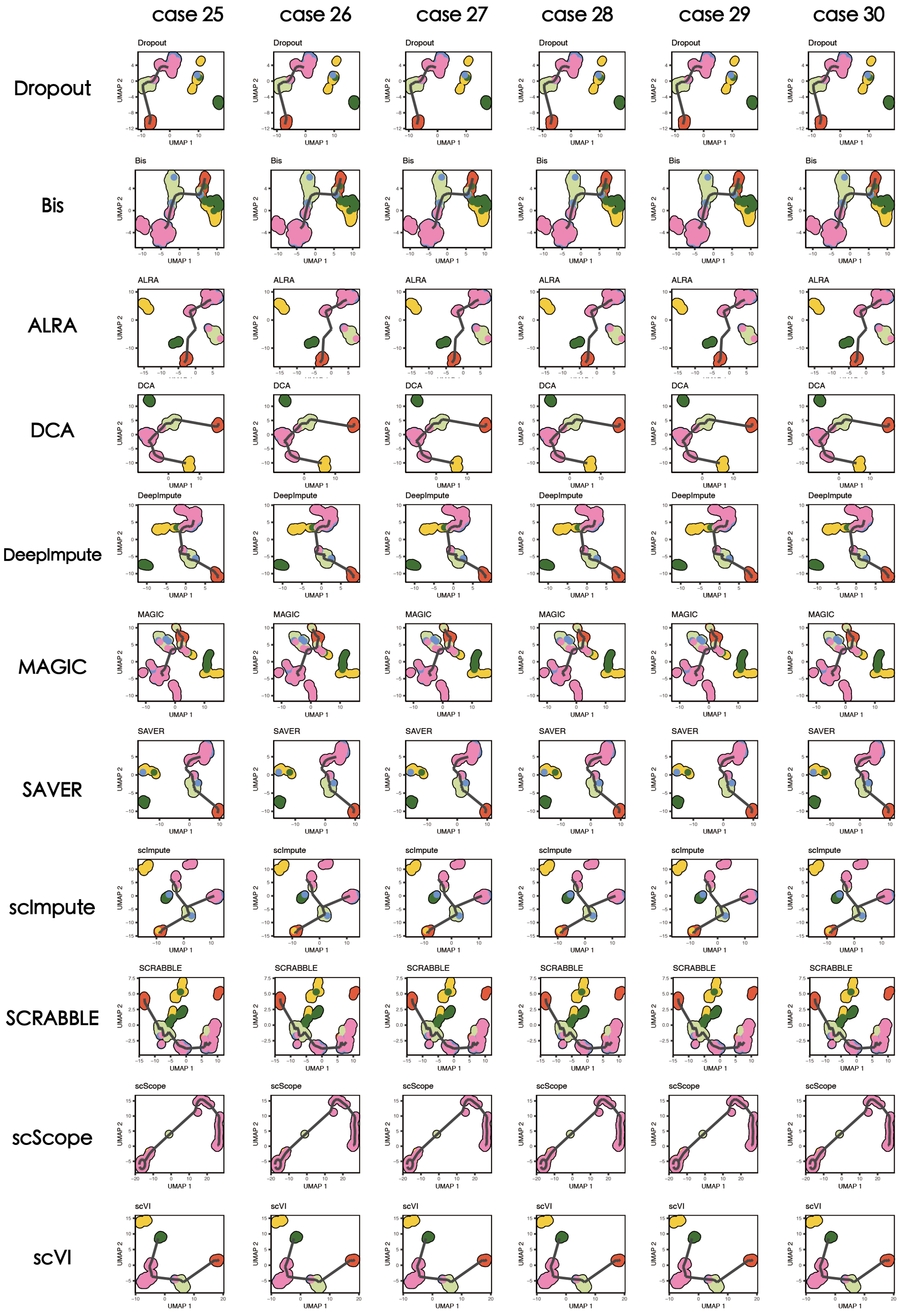

Figure 13: The cases 25-30 of trajectories reconstructed by Monocle3 from the imputed scRNA-seq data using all methods.

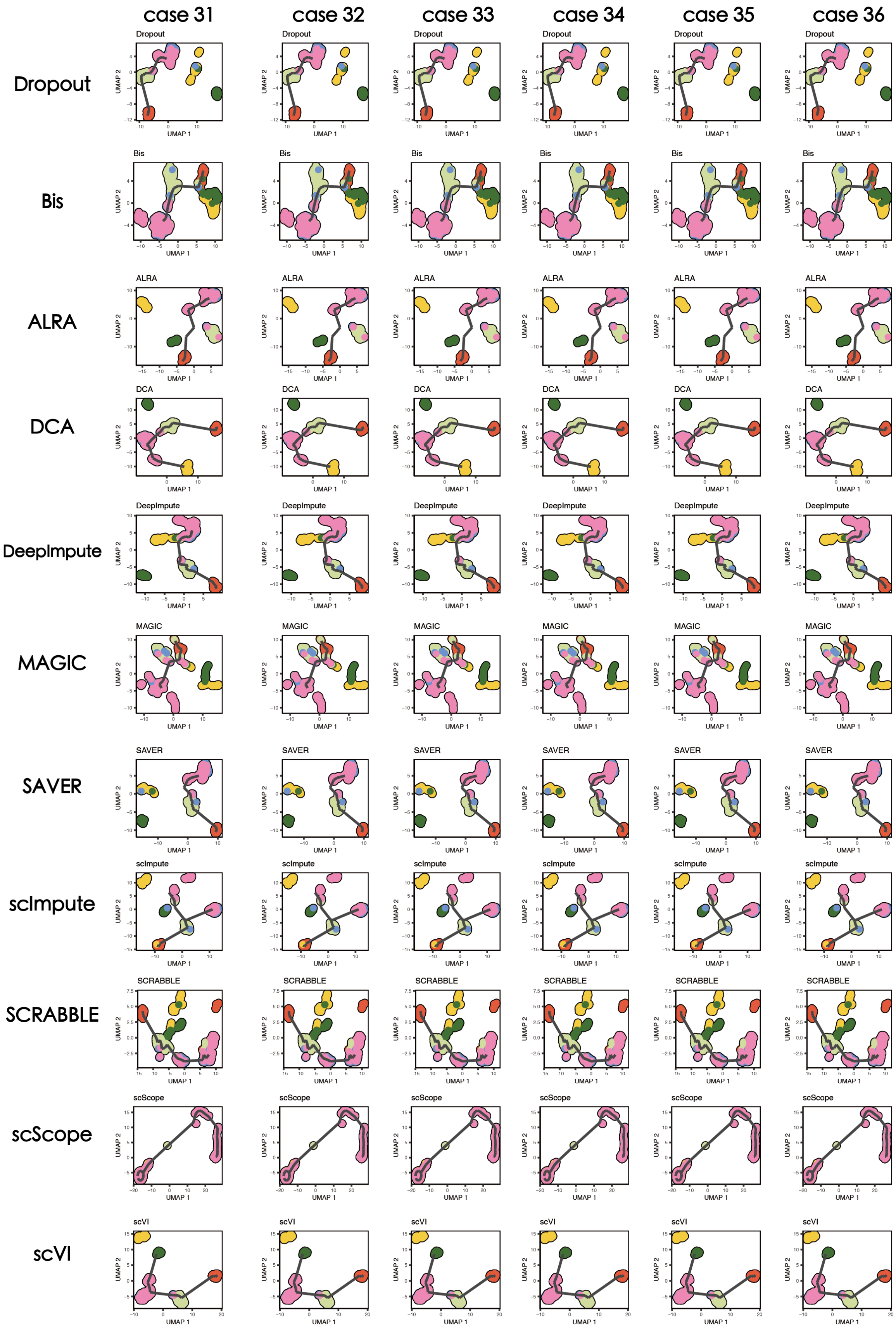

Figure 14: The cases 31-36 of trajectories reconstructed by Monocle3 from the imputed scRNA-seq data using all methods.

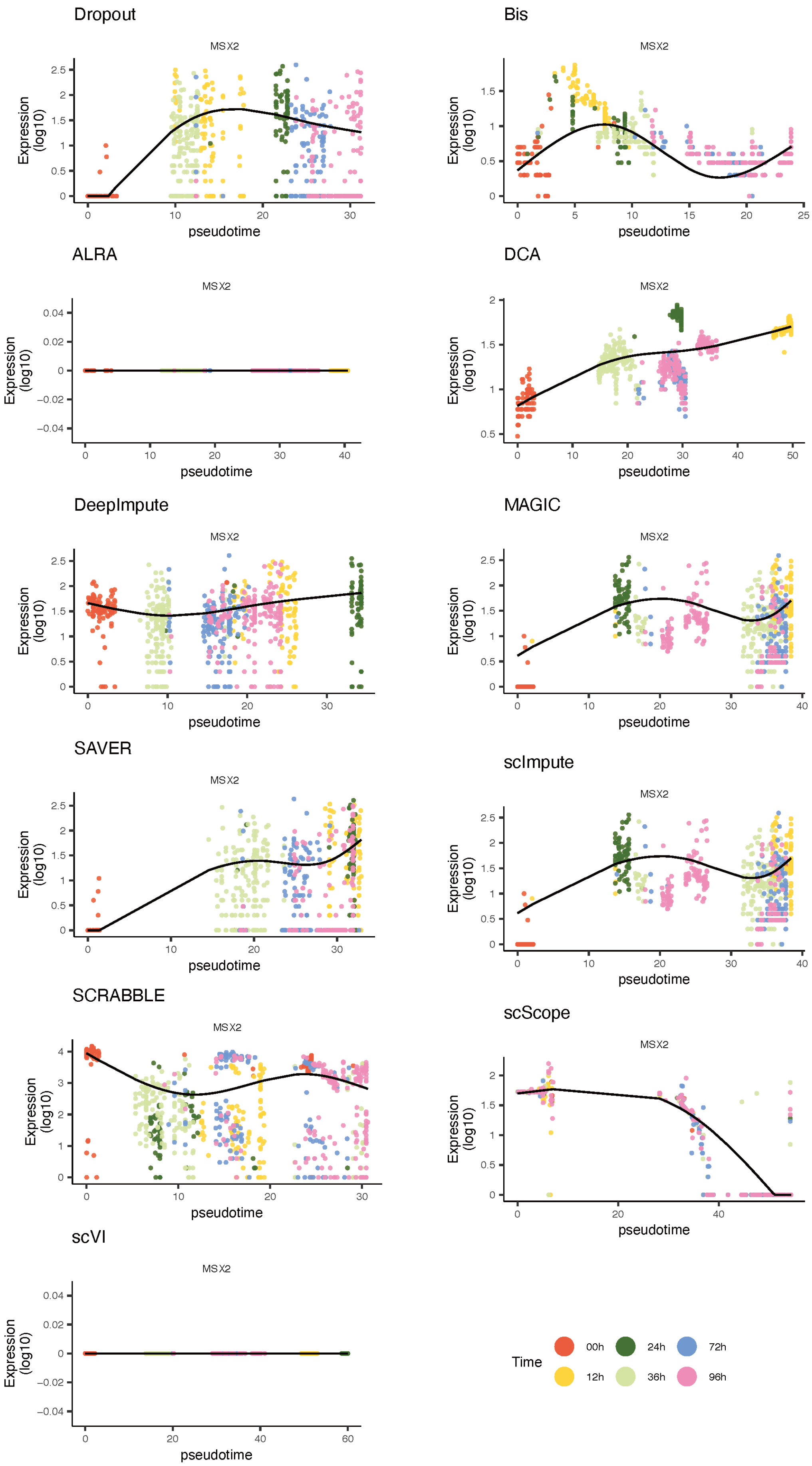

Figure 15: The expression dynamics of Key DE gene MSX2 are shown in the order of the pseudotime.

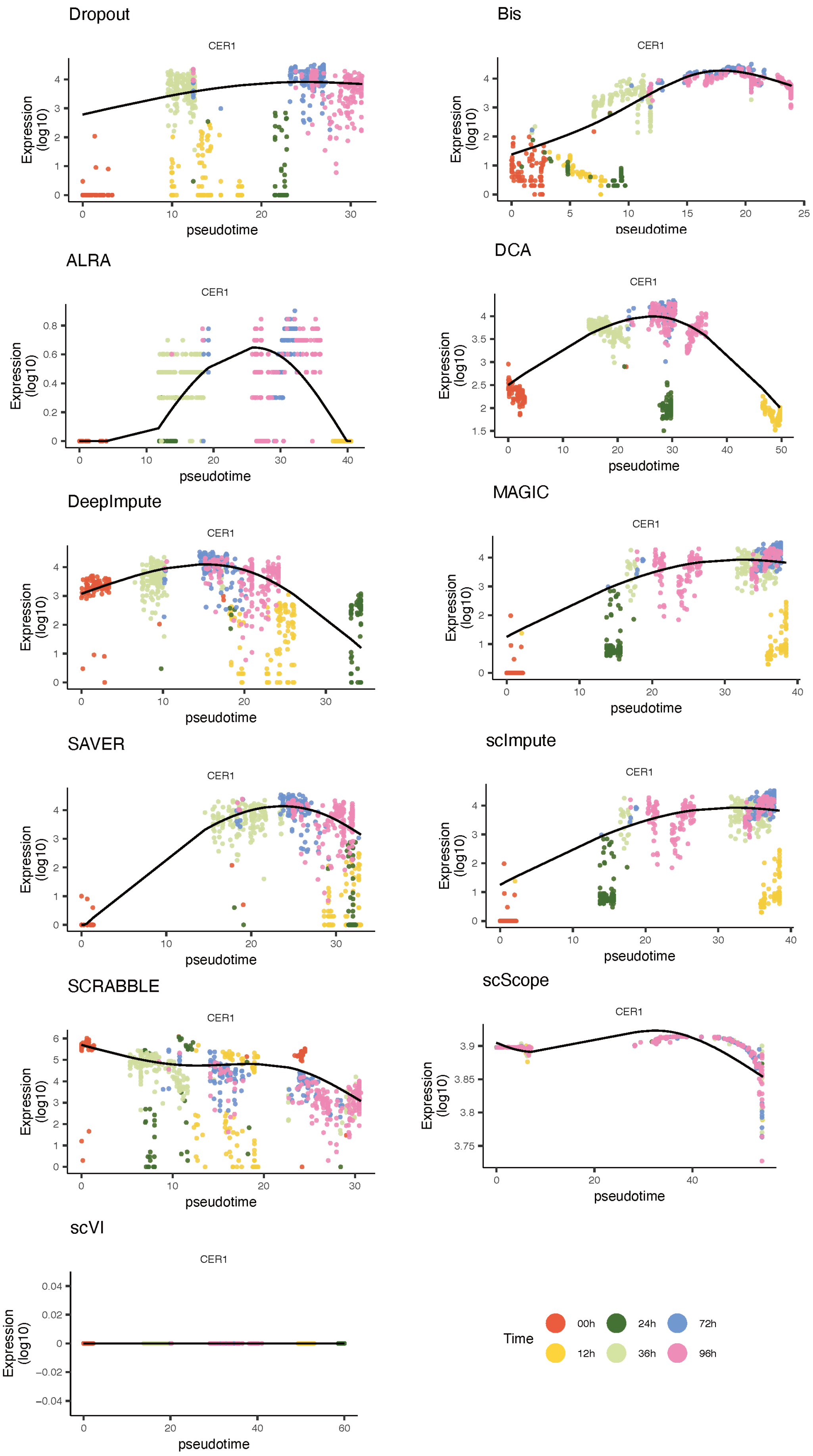

Figure 16: The expression dynamics of Key DE gene CER1 are shown in the order of the pseudotime.

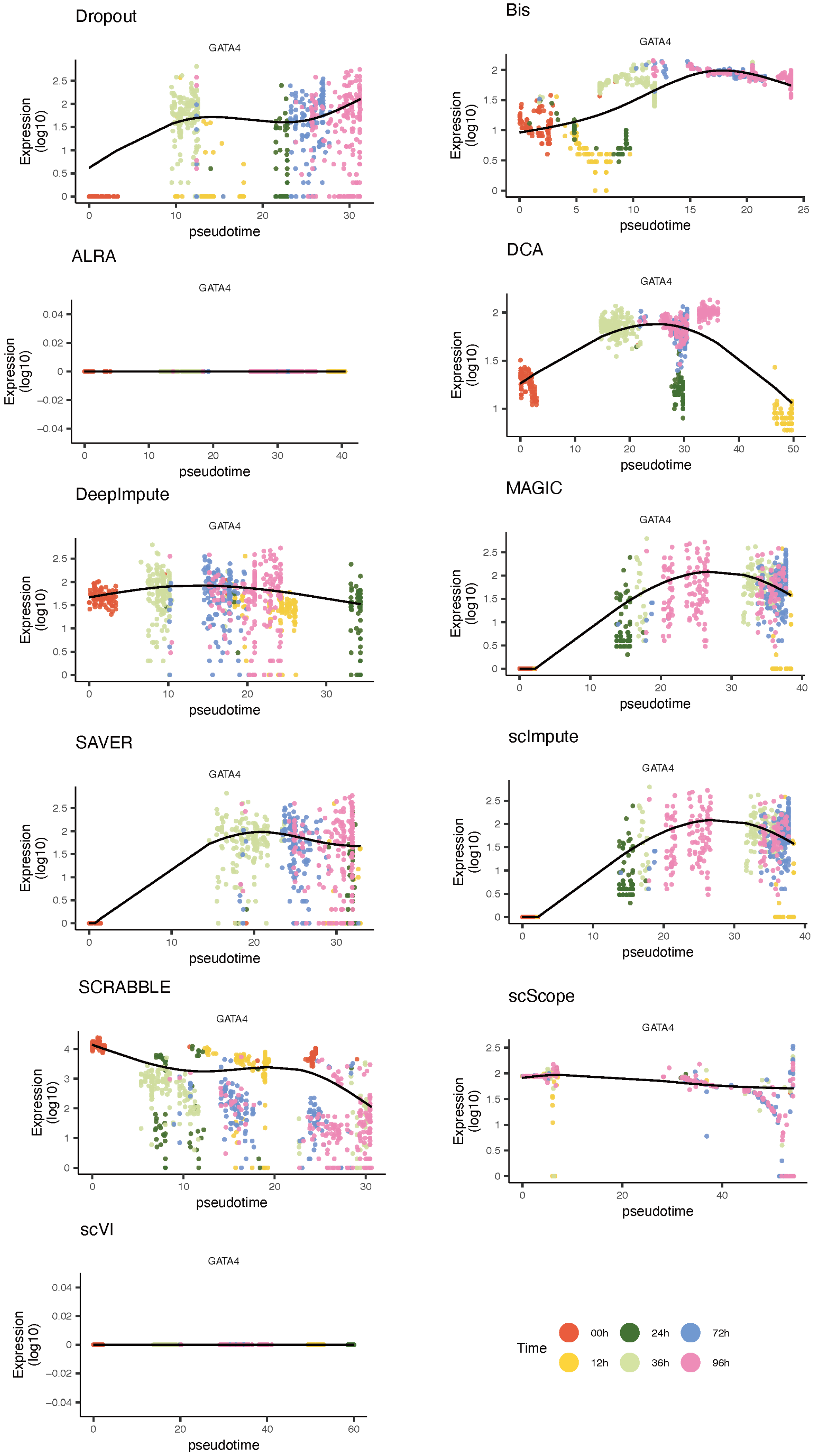

Figure 17: The expression dynamics of Key DE gene GATA4 are shown in the order of the pseudotime.

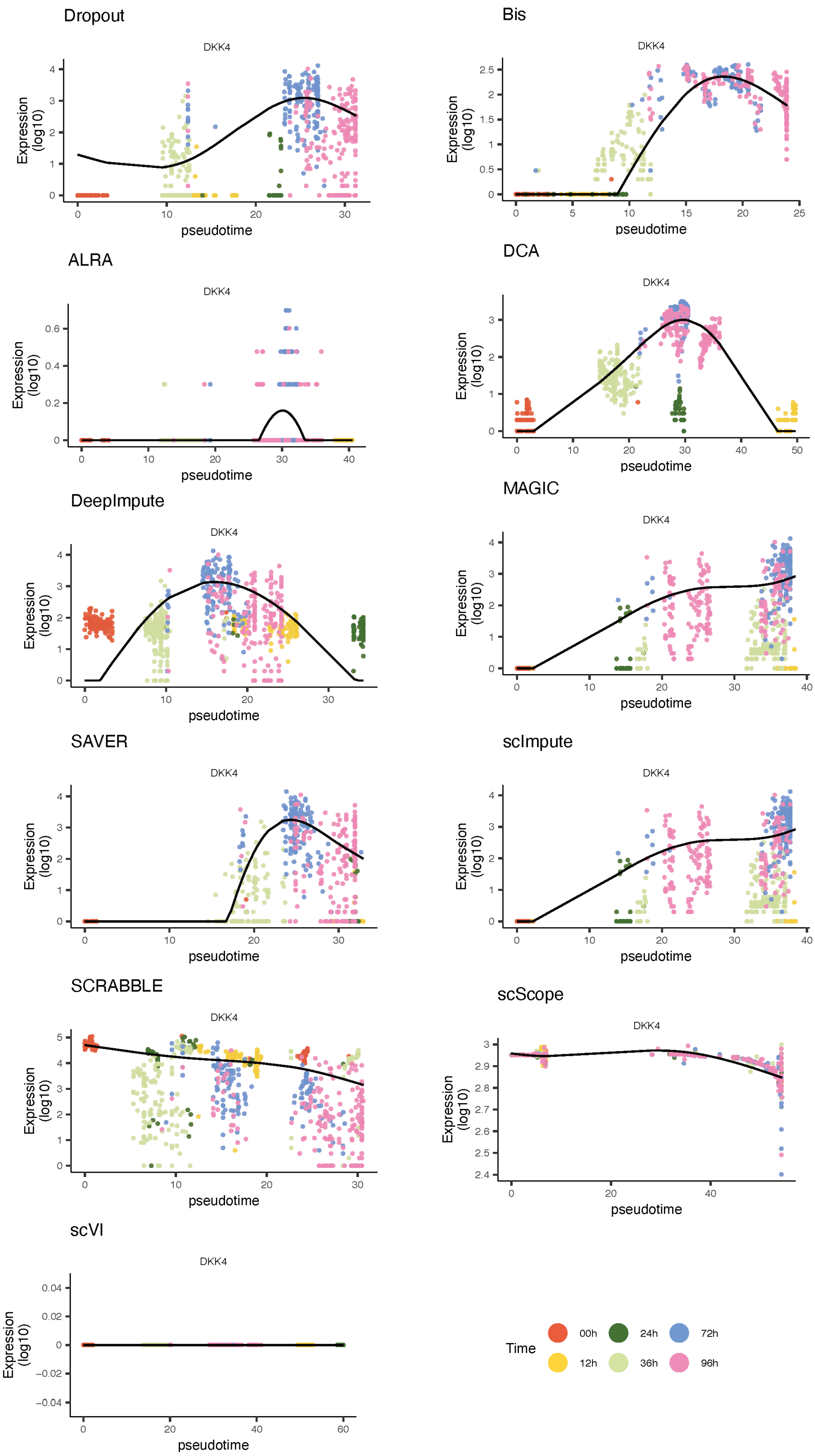

Figure 18: The expression dynamics of Key DE gene DKK4 are shown in the order of the pseudotime.

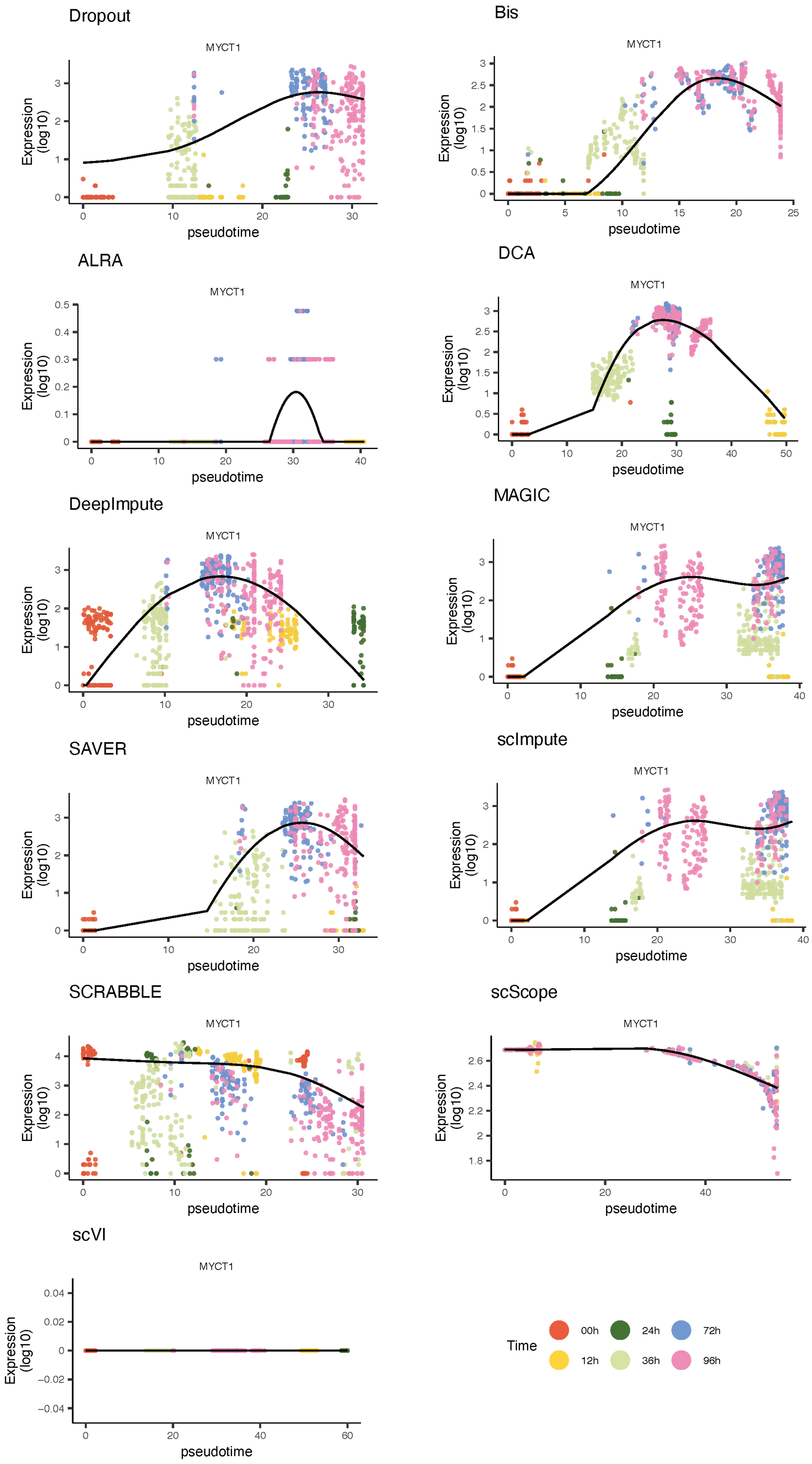

Figure 19: The expression dynamics of Key DE gene MYCT1 are shown in the order of the pseudotime.

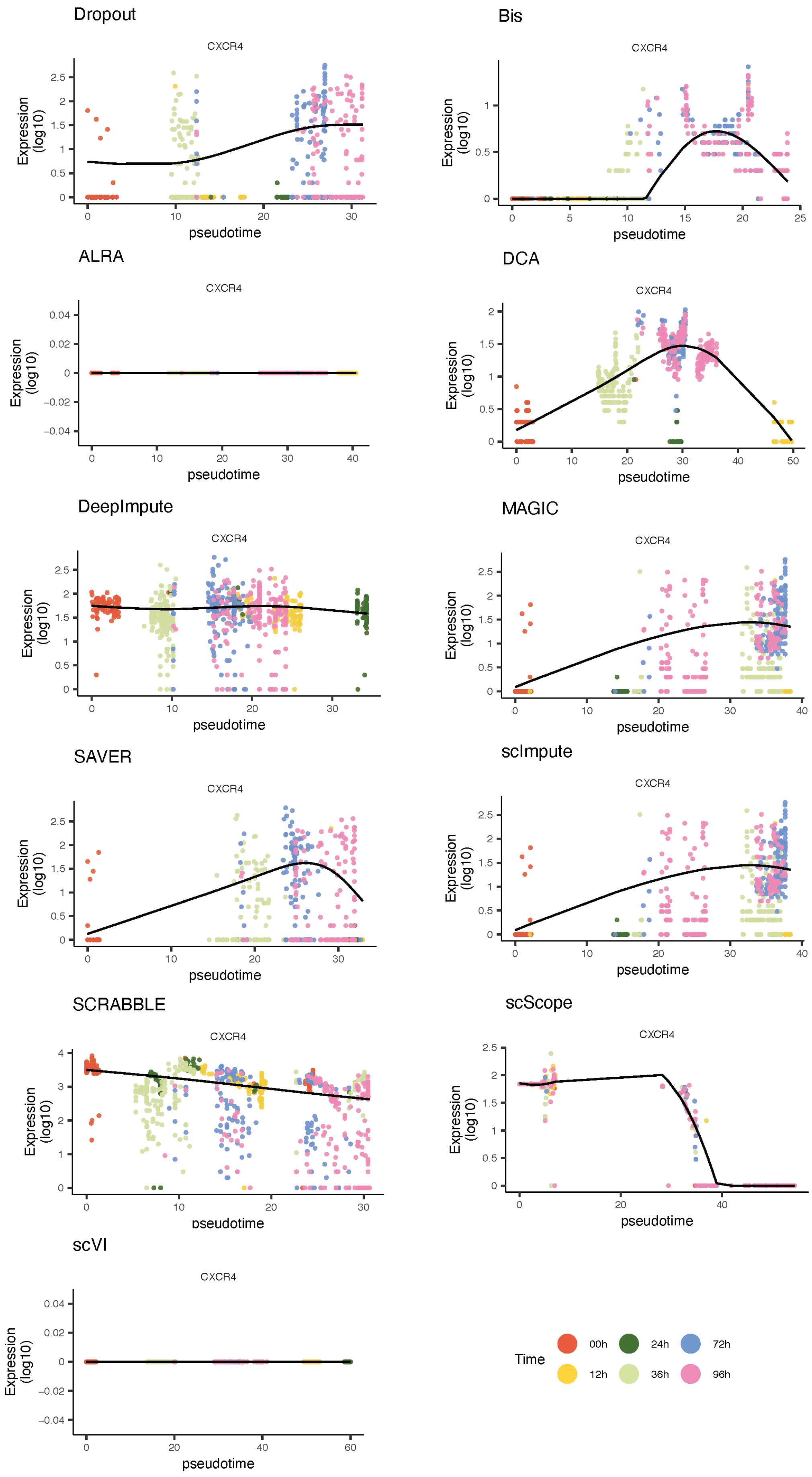

Figure 20: The expression dynamics of Key DE gene CXCR4 are shown in the order of the pseudotime.

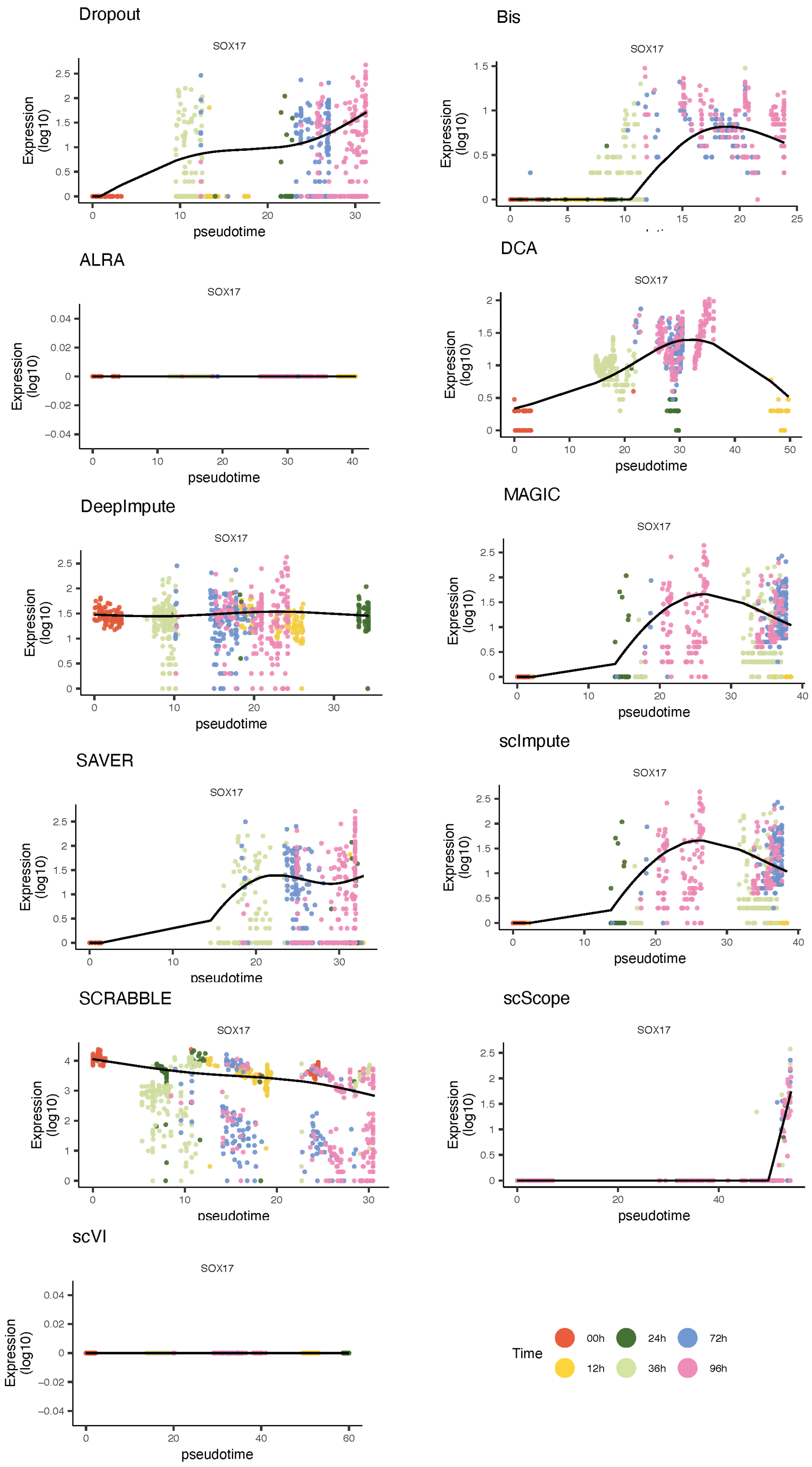

Figure 21: The expression dynamics of Key DE gene SOX17 are shown in the order of the pseudotime.

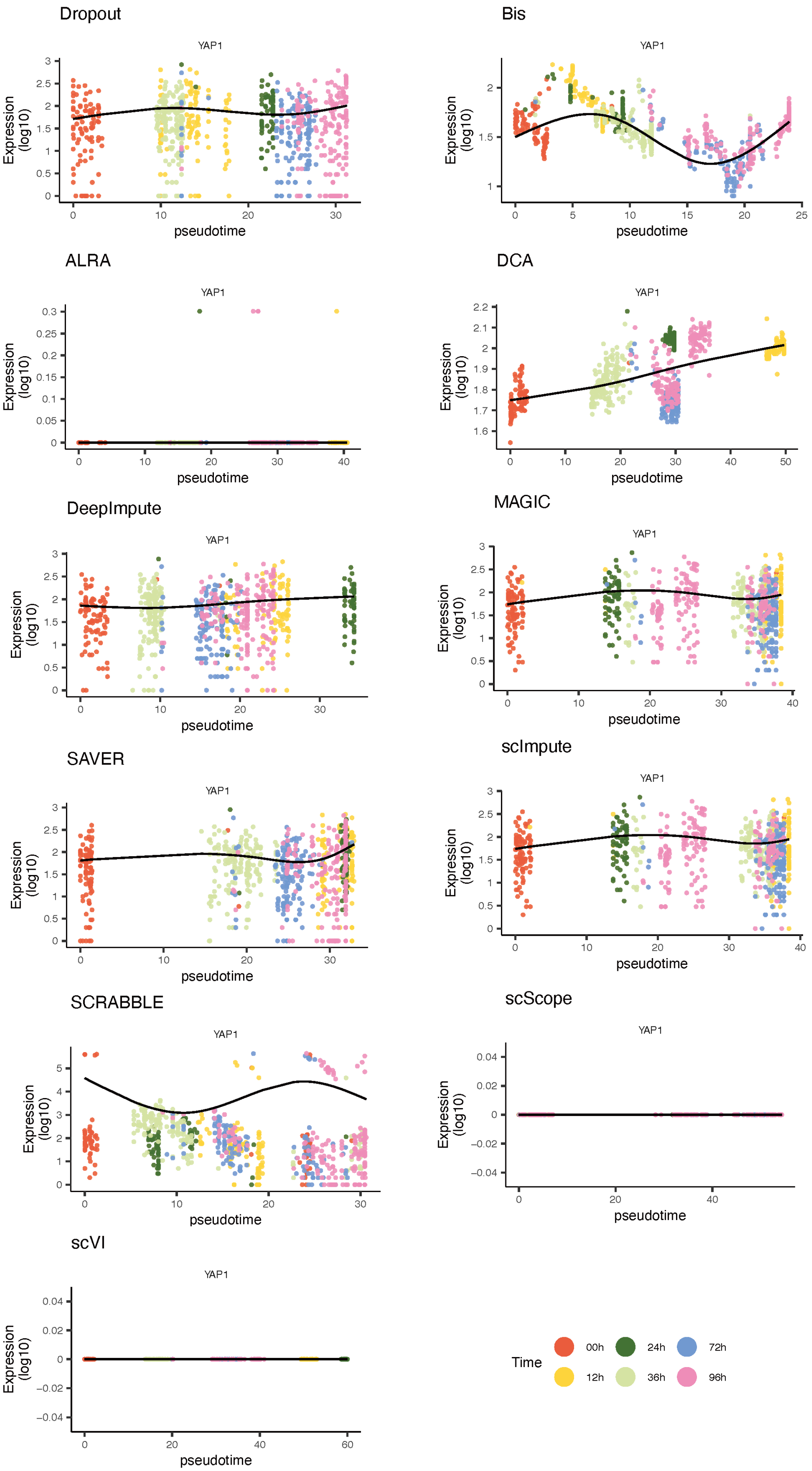

Figure 22: The expression dynamics of Key DE gene YAP1 are shown in the order of the pseudotime.

7 Bis can reveal the developmental characteristic of cytokine-induced memory-like natural killer cells in the circulating immune microenvironment

Table 9: HNSCC patient information: Patient demographics and HPV status of patients included in the cohort for scRNASeq and Flow cytometry validation.

HN #	Gender	Age group	Smoking	Alcohol	Disease site	*T-Stage	*N-Stage	*M-Stage	HPV (p16 IHC)	Inflam. status	Analysis
1	M	70-79	Yes	No	Oral cavity	T4A	N2B	M0	Neg	High	scRNAseq, IHC
2	F	60-69	No	No	Oral cavity	T3	N2a	M0	Neg	Low	scRNAseq
3	M	80-89	No	No	Oral cavity	T4a	N0	M0	Neg	NA	scRNAseq
4	M	50-59	Yes	Yes	Oral cavity	T3	N1	M0	Neg	Low	scRNAseq
5	F	50-59	Yes	Yes	Oral cavity	†T3	†N3b	†M0	Neg	Med	scRNAseq
6	M	30-39	Yes	Yes	Oral cavity	T3	N0	M0	Neg	High	scRNAseq
7	M	60-69	Yes	Yes	Larynx	T3	N0	M0	Neg	Low	scRNAseq
8	F	70-79	Yes	Yes	Oral cavity	†T1	†N0	†M0	Neg	Med	scRNAseq, IHC
9	F	70-79	Yes	Yes	Oral cavity	T3	N2B	M0	Neg	Med	scRNAseq, IHC, Vectra
10	M	50-59	No	Yes	Oral cavity	T3	N0	M0	Neg	High	scRNAseq
11	M	80-89	No	No	Oral cavity	T2	N0	M0	Neg	Low	scRNAseq
12	M	50-59	Yes	Yes	Oropharynx	T2	N1	M0	Pos	Med	scRNAseq, IHC, Vectra
13	M	70-79	No	No	Oropharynx	T2	N0	M0	Pos	High	scRNAseq, IHC, Vectra
14	M	50-59	Yes	Yes	Oropharynx	T1	N1	M0	Pos	High	scRNAseq, IHC, Vectra
15	F	60-69	Yes	NA	Oral cavity	T2	N0	M0	Neg	Low	scRNAseq, IHC, Vectra
16	M	40-49	Yes	Yes	Oropharynx	T2	N1	M0	Pos	Med	scRNAseq, IHC
17	M	50-59	Yes	Yes	Oropharynx	T1	N1	M0	Pos	High	scRNAseq, IHC
18	M	50-59	Yes	Yes	Oropharynx	T2	N2	M0	Pos	Low	scRNAseq, IHC, Vectra

***TNM Eight Edition AJCC guidelines for clinical staging (with exception of patients 5 and 8)**

†Pathologically staged

NA - not available

IHC - immunohistochemistry fibroblast staining

Vectra - multispectral imaging

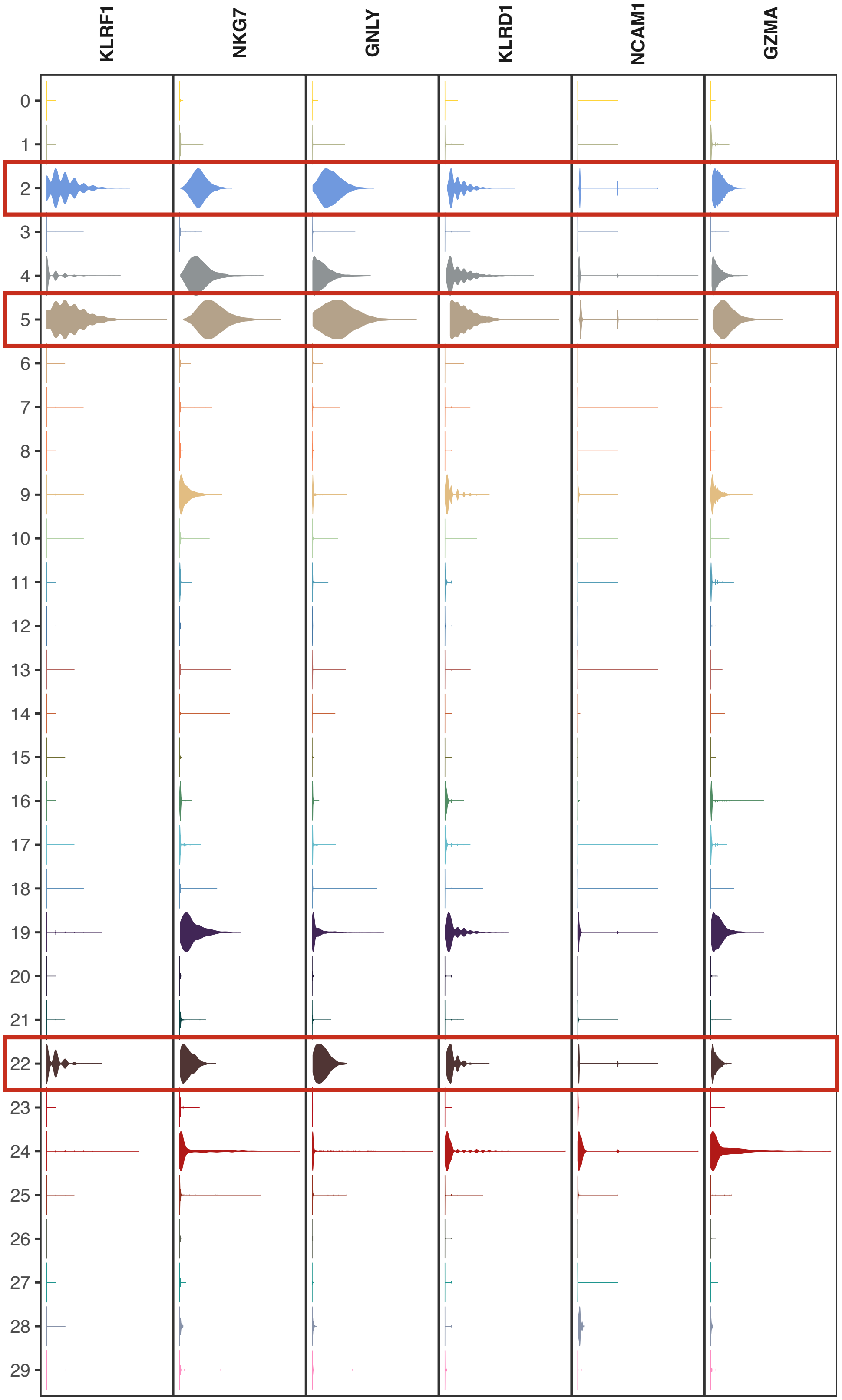

Figure 23: Violin plots representing the expression of marker genes of NK cells. Clusters 2, 5, and 22 Share similar expression patterns in marker genes.

	Top 10	Secreted	Cell membrane	Transcription factors
hNK_Bl1	*FGFBP2*<br>*GZMB*<br>*GZMA*<br>*SPON2*<br>*S100A4*<br>*CST7*<br>*FCGR3A*<br>*IGFBP7*<br>*GZMH*<br>*CFL1*	*FGFBP2*<br>*GZMB*<br>*GZMA*<br>*SPON2*<br>*CST7*<br>*IGFBP7*<br>*GZMH*<br>*LGALS1*<br>*LAIR2*<br>*PRF1*	*FCGR3A*<br>*CFL1*<br>*ITGB2*<br>*CYBA*<br>*CD247*<br>*KIR2DL3*<br>*KLRD1*<br>*LCP1*<br>*CD164*<br>*EMP3*	*GTF3C1*
hNK_Bl2	*GZMK*<br>*CD44*<br>*PPP1R14B*<br>*CXCR3*<br>*RPL36A*<br>*SCML1*<br>*COTL1*<br>*NCF1*<br>*XCL1*<br>*HLA-DRB1*	*GZMK*<br>*XCL1*<br>*LTB*<br>*SPOCK2*	*CD44*<br>*CXCr3*<br>*NCF1*<br>*HLA-DRB1*<br>*CD83*<br>*CD82*	*SCML1*<br>*ZFP36L2*

Figure 24: Top ten genes significantly differentiating between the two blood NK cell subsets in one representative sample.

gene	p_val	cluster	gene	p_val	cluster	gene	p_val	cluster	gene	p_val	cluster
SPON2	8.19E-142	BI1	LY6E	4.71E-16	BI1	GZMK	2.00E-115	BI2	RPL27	1.37E-21	BI2
NKG7	1.83E-112	BI1	RAP1B	5.83E-16	BI1	EEF1A1	1.19E-103	BI2	CD3E	2.35E-21	BI2
ACTB	3.75E-111	BI1	PDIA3	7.31E-16	BI1	RPS2	1.71E-83	BI2	PIK3R1	2.12E-20	BI2
PFN1	1.89E-98	BI1	DHRS7	8.54E-16	BI1	RPL13	4.42E-82	BI2	RPL4	3.61E-19	BI2
TMSB10	1.35E-96	BI1	PPIB	9.11E-16	BI1	RPL13A	1.46E-78	BI2	CD2	7.43E-19	BI2
PRF1	1.77E-95	BI1	SDF2L1	1.10E-15	BI1	RPS15A	1.06E-76	BI2	RPL24	1.02E-18	BI2
FGFBP2	1.98E-94	BI1	TBC1D10C	1.47E-15	BI1	XCL1	6.67E-75	BI2	CLDND1	1.23E-18	BI2
S100A4	3.11E-91	BI1	MANF	3.46E-15	BI1	RPS27	5.08E-73	BI2	RPS10	5.43E-18	BI2
CFL1	5.47E-85	BI1	UCP2	1.49E-14	BI1	SELL	1.08E-72	BI2	IFITM3	7.25E-18	BI2
HLA-B	1.44E-82	BI1	CTSC	1.51E-14	BI1	XCL2	5.67E-66	BI2	CD69	7.88E-18	BI2
AKR1C3	1.54E-63	BI1	CAP1	1.72E-14	BI1	RPS12	7.66E-65	BI2	NEIL1	9.09E-18	BI2
ARPC2	4.99E-63	BI1	RHOA	6.49E-14	BI1	RPS29	9.49E-63	BI2	AC044849.1	3.18E-17	BI2
PTGDS	9.38E-63	BI1	EBP	1.09E-13	BI1	RPL34	2.30E-62	BI2	IER2	5.20E-17	BI2
ACTG1	1.34E-55	BI1	GNG2	1.53E-13	BI1	RPS6	4.42E-61	BI2	PPP1R15A	5.58E-17	BI2
GZMB	1.25E-54	BI1	NCR3	3.67E-13	BI1	RPS28	9.50E-61	BI2	IFRD1	1.39E-16	BI2
ALOX5AP	1.19E-51	BI1	NDUFB2	5.10E-13	BI1	RPL41	4.36E-59	BI2	SOCS1	5.70E-16	BI2
IGFBP7	9.45E-50	BI1	ANXA4	5.19E-13	BI1	RPL10	1.22E-58	BI2	CD74	7.41E-16	BI2
FCGR3A	7.94E-48	BI1	PPP1R18	5.30E-13	BI1	RPS14	1.48E-57	BI2	NR4A2	1.05E-15	BI2
SERF2	9.07E-44	BI1	PSMA7	5.66E-13	BI1	RPL3	2.52E-57	BI2	TCF7	2.17E-15	BI2
S100A6	5.68E-43	BI1	FAM49B	5.67E-13	BI1	CMC1	1.92E-56	BI2	RPL23	6.27E-14	BI2
LGALS1	9.39E-43	BI1	SUB1	6.05E-13	BI1	RPLP2	1.06E-55	BI2	BTG1	2.39E-12	BI2
ITGB2	1.06E-42	BI1	TRAPPC1	7.39E-13	BI1	RPL32	1.10E-55	BI2	JUN	2.71E-12	BI2
CST7	8.90E-41	BI1	ABI3	7.41E-13	BI1	RPS23	1.19E-55	BI2	TSC22D3	5.74E-12	BI2
CD247	8.71E-40	BI1	ISG15	7.97E-13	BI1	DUSP2	1.79E-54	BI2	FXYD5	1.08E-11	BI2
CD99	9.26E-38	BI1	LCP1	8.54E-13	BI1	RPS18	4.26E-54	BI2	SH2D1A	1.45E-11	BI2
MYL6	9.50E-38	BI1	PSAP	1.00E-12	BI1	RPL23A	1.72E-53	BI2	PDCD4	1.71E-11	BI2
DBI	1.85E-37	BI1	PREX1	1.39E-12	BI1	RPS19	6.52E-52	BI2	SNHG8	3.05E-11	BI2
CTSD	6.11E-37	BI1	ZEB2	1.56E-12	BI1	RPL18A	1.47E-50	BI2	CNN2	7.43E-11	BI2
MYL12A	6.29E-37	BI1	TMED9	1.56E-12	BI1	DUSP1	2.14E-49	BI2	LTB	1.24E-10	BI2
HES6	2.94E-36	BI1	ENO1	1.84E-12	BI1	RPS3	1.67E-47	BI2	FOSL2	1.73E-10	BI2
UBB	7.39E-33	BI1	COX6C	1.90E-12	BI1	RPS3A	2.06E-47	BI2	KLRC2	1.85E-10	BI2
MYOM2	1.08E-32	BI1	UBL5	2.28E-12	BI1	TPT1	6.62E-47	BI2	MAP3K8	2.14E-10	BI2
AK5	3.20E-31	BI1	TPM4	2.53E-12	BI1	RPL39	1.01E-46	BI2	IER5	3.64E-10	BI2
EFHD2	1.26E-30	BI1	CD38	2.58E-12	BI1	RPS8	1.14E-46	BI2	TENT5C	5.97E-10	BI2
S100A11	1.27E-30	BI1	S100B	2.59E-12	BI1	RPL31	2.09E-46	BI2	GCHFR	9.15E-10	BI2
ABHD17A	1.64E-29	BI1	SPCS2	4.05E-12	BI1	RPL27A	2.73E-45	BI2	HLA-DRA	4.34E-09	BI2
RAC2	2.11E-29	BI1	CD164	4.16E-12	BI1	RPL21	3.68E-45	BI2	HLA-DQB1	7.47E-09	BI2
ARHGDIB	4.10E-29	BI1	MTSS1	5.97E-12	BI1	RPS25	1.01E-44	BI2	TMEM123	8.05E-09	BI2
GZMM	1.16E-27	BI1	NEDD8	6.51E-12	BI1	FOS	3.73E-44	BI2	LINC01871	2.66E-08	BI2
CALR	2.45E-27	BI1	GIMAP7	1.32E-11	BI1	RPS21	3.75E-44	BI2	HLA-DRB1	4.45E-08	BI2
CTSW	3.93E-27	BI1	LAT2	1.37E-11	BI1	RPS27A	1.17E-43	BI2	IRF1	9.43E-08	BI2
KLRB1	5.97E-27	BI1	RAC1	1.61E-11	BI1	RPS16	7.51E-42	BI2	RHOH	1.07E-07	BI2
TTC38	1.26E-26	BI1	PRSS23	1.65E-11	BI1	CD44	1.34E-40	BI2	ZFP36	1.31E-07	BI2
CX3CR1	1.45E-26	BI1	AGTRAP	2.05E-11	BI1	RPL10A	1.36E-39	BI2	TNFAIP3	1.42E-07	BI2
CORO1A	9.64E-26	BI1	TMEM258	2.36E-11	BI1	RPL19	1.67E-39	BI2	AC103591.3	2.24E-07	BI2
ADGRG1	1.28E-25	BI1	CYCS	2.41E-11	BI1	MAFF	1.69E-39	BI2	B3GNT7	2.37E-07	BI2
CD6	2.44E-25	BI1	HSPA5	2.57E-11	BI1	NFKBIA	4.30E-39	BI2	B4GALT1	2.62E-06	BI2
FLNA	1.79E-24	BI1	CD160	2.66E-11	BI1	RPL26	6.89E-39	BI2	PMAIP1	4.94E-06	BI2
FCER1G	4.30E-24	BI1	SPN	2.81E-11	BI1	RPS24	1.38E-37	BI2	CCL5	9.89E-57	BI3
LAIR2	6.09E-24	BI1	PRDX5	3.17E-11	BI1	RPL30	1.46E-37	BI2	KLRC11	1.12E-49	BI3
CHST2	9.68E-24	BI1	PGAM1	5.15E-11	BI1	RPL37	1.50E-37	BI2	RPS191	2.71E-33	BI3
CALM1	1.05E-23	BI1	IFI27L2	7.45E-11	BI1	RPLP1	2.18E-37	BI2	HLA-DRB5	1.05E-29	BI3
CCL4	1.29E-23	BI1	GLRX	1.76E-10	BI1	RPL28	1.14E-36	BI2	CD52	5.33E-29	BI3
AES	1.46E-23	BI1	CST3	1.77E-10	BI1	RPL18	2.30E-36	BI2	LINC018711	4.23E-28	BI3
CAPZB	1.61E-23	BI1	TMEM59	1.78E-10	BI1	RPL35	4.58E-36	BI2	GZMH	7.42E-23	BI3
PLAC8	2.15E-23	BI1	OSTF1	1.79E-10	BI1	RPSA	4.66E-36	BI2	RPS26	3.96E-22	BI3
PRSS57	4.69E-23	BI1	ITGAL	1.94E-10	BI1	RPL7	9.47E-36	BI2	HLA-DPB1	1.32E-21	BI3
EMP3	8.67E-23	BI1	KIR3DL2	2.01E-10	BI1	RPL35A	1.06E-35	BI2	PATL2	2.19E-20	BI3
GZMA	1.54E-22	BI1	ZNHIT1	2.12E-10	BI1	RPLP0	1.30E-34	BI2	GNLY	4.21E-19	BI3
TMIGD2	7.25E-22	BI1	FEZ1	2.55E-10	BI1	RPS5	8.98E-34	BI2	ANXA5	4.82E-14	BI3
ITGB7	7.29E-22	BI1	FGR	3.12E-10	BI1	RPL9	9.55E-34	BI2	CD3E1	5.03E-13	BI3
PPP1CA	4.76E-21	BI1	P4HB	3.19E-10	BI1	RPL8	2.20E-33	BI2	ZFAS1	2.15E-12	BI3
SH3BGRL3	4.84E-21	BI1	MMP23B	3.79E-10	BI1	RPL36	2.45E-32	BI2	JAKMIP1	5.42E-12	BI3
CLIC1	5.84E-20	BI1	MINOS1	4.07E-10	BI1	RPS13	2.79E-32	BI2	HLA-DPA1	8.95E-12	BI3
PSME2	1.82E-19	BI1	MYDGF	4.99E-10	BI1	RPL5	7.53E-32	BI2	LYAR	5.15E-11	BI3
EVL	2.12E-19	BI1	HAVCR2	8.27E-10	BI1	AREG	6.82E-31	BI2	ITGB1	2.48E-08	BI3
FCRL6	2.20E-19	BI1	TLN1	8.93E-10	BI1	RPL6	3.12E-30	BI2	PRSS231	2.08E-07	BI3
ARPC1B	4.22E-19	BI1	CALM3	9.42E-10	BI1	RPL36A	4.22E-30	BI2	CD21	1.36E-05	BI3
RHOC	1.31E-18	BI1	SCP2	1.00E-09	BI1	COTL1	1.65E-29	BI2	RPS4Y1	4.24E-05	BI3
ANXA2	1.80E-18	BI1	DYNLL1	2.01E-09	BI1	RPL37A	3.19E-29	BI2	DSTN	0.00027805	BI3
ARPC3	1.85E-18	BI1	DYNLRB1	2.45E-09	BI1	RPS7	3.80E-28	BI2	DRAM2	0.00089667	BI3
SYNGR1	4.34E-18	BI1	APOBEC3C	2.70E-09	BI1	RPS9	9.88E-28	BI2	SERPINB1	0.00184702	BI3
MARCKSL1	4.43E-18	BI1	GNG5	2.84E-09	BI1	RPL12	2.58E-27	BI2	HLA-DRB11	0.00332732	BI3
CLIC3	5.38E-18	BI1	VASP	3.32E-09	BI1	RPS4X	7.83E-27	BI2			
MYL12B	1.07E-17	BI1	CISD3	3.36E-09	BI1	RPL29	1.22E-26	BI2			
APLP2	1.56E-17	BI1	DDIT4	3.74E-09	BI1	KLRC1	2.24E-26	BI2			
MSN	3.54E-17	BI1	STARD3NL	4.62E-09	BI1	MCUB	3.79E-26	BI2			
LSP1	3.77E-17	BI1	TES	6.76E-09	BI1	EEF1B2	6.29E-25	BI2			
KIR2DL3	3.78E-17	BI1	NDUFA12	2.87E-08	BI1	RPS17	8.93E-25	BI2			
WDR1	7.63E-17	BI1	FERMT3	2.93E-08	BI1	CXCR4	1.46E-24	BI2			
MAPK1	1.53E-16	BI1	ATOX1	3.01E-08	BI1	RPS20	2.36E-24	BI2			
GLIPR2	2.58E-16	BI1	PDIA6	3.39E-08	BI1	ZFP36L2	1.10E-23	BI2			
TPM3	3.32E-16	BI1	CCL3	3.76E-07	BI1	CAPN12	1.44E-23	BI2			
HMOX2	4.40E-16	BI1	IL32	1.22E-06	BI1	NOP53	3.85E-22	BI2			
			PCBP1	1.92E-06	BI1	RPL17	1.15E-21	BI2			

Figure 25: Gene signatures of the three CIML NK cell subsets from human peripheral blood samples are shown. Adjusted p-values with non-parametric Wilcoxon rank-sum tests are provided.

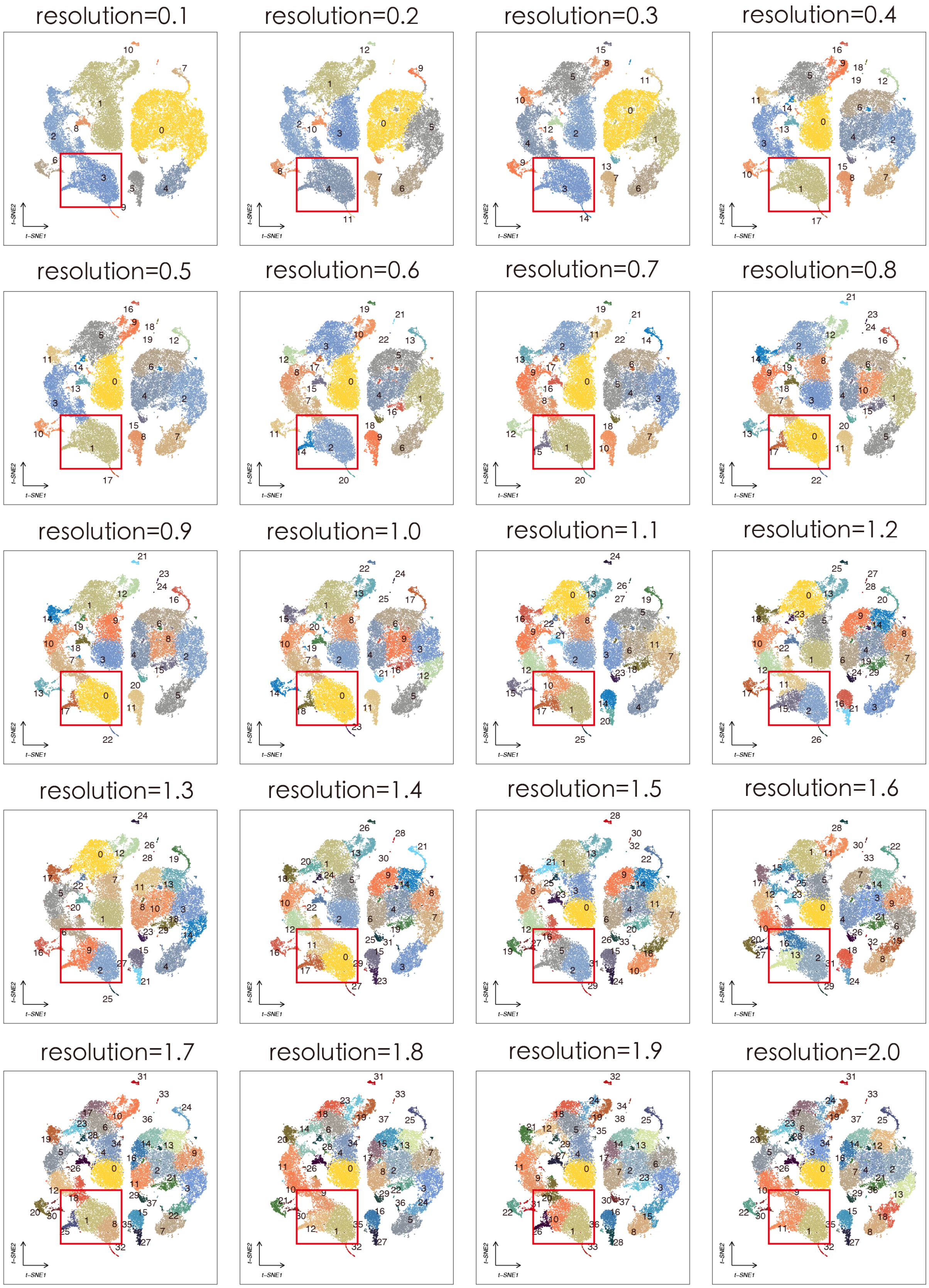

Figure 26: t-SNE plots visualizing the peripheral blood dataset before imputation colored by cell clusters with different resolution.

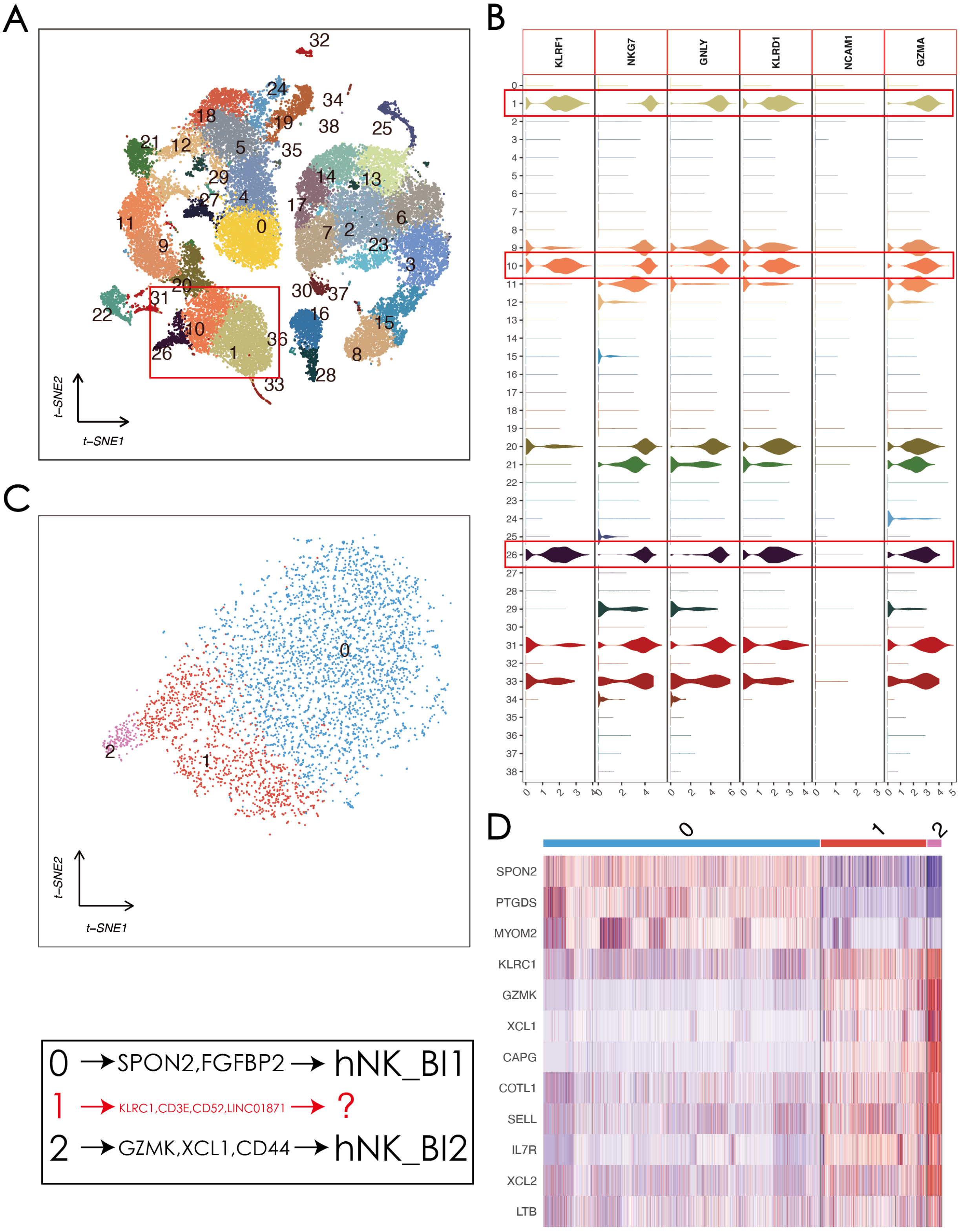

Figure 27: **(A)** t-SNE plots visualizing the peripheral blood dataset before imputation colored by cell clusters. **(B)** Violin plots representing the expression of marker genes of NK cells. Clusters 1, 10, and 26 Share similar expression patterns in marker genes. **(C)** t-SNE plots visualizing NK cells colored by cell clusters. **(D)** Heatmaps of selected differentially expressed genes in the three cell subsets.

Table 10: The cluster biomarkers obtained from differential analysis using Seurat.

avg_log2FC	p_val_adj	cluster	gene	resolution	avg_log2FC	p_val_adj	cluster	gene	resolution
4.014794	0	3	GNLY	0.1	1.308272	0	3	FCRL6	0.1
3.470928	0	3	NKG7	0.1	1.288891	0	3	AKR1C3	0.1
3.434522	0	3	GZMB	0.1	1.287678	0	3	RARRES3	0.1
3.325065	0	3	FGFBP2	0.1	1.281903	0	3	CD63	0.1
3.175444	0	3	PRF1	0.1	1.276118	0	3	APOBEC3G	0.1
3.066611	0	3	SPON2	0.1	1.274997	0	3	ID2	0.1
2.835306	0	3	KLRF1	0.1	1.255398	0	3	BIN2	0.1
2.828038	0	3	CST7	0.1	1.235393	0	3	RHOC	0.1
2.824821	0	3	GZMA	0.1	1.231728	0	3	TPST2	0.1
2.806212	0	3	CTSW	0.1	1.220501	0	3	RAC2	0.1
2.680158	0	3	PTGDS	0.1	1.210044	0	3	RAP1B	0.1
2.678029	0	3	HOPX	0.1	1.208003	0	3	SH2D1B	0.1
2.583951	0	3	CLIC3	0.1	1.19826	0	3	RUNX3	0.1
2.474782	0	3	KLRD1	0.1	1.196893	0	3	AREG	0.1
2.453565	0	3	CD7	0.1	1.189436	0	3	C1orf21	0.1
2.412211	0	3	CCL5	0.1	1.173913	0	3	ZAP70	0.1
2.336695	0	3	KLRB1	0.1	1.17048	0	3	IFITM2	0.1
2.329664	0	3	CCL4	0.1	1.169794	0	3	CD99	0.1
2.301212	0	3	TRDC	0.1	1.163415	0	3	PTGDR	0.1
2.239124	0	3	CD247	0.1	1.162951	0	3	XCL2	0.1
2.227545	0	3	CMC1	0.1	1.160634	0	3	HLA-A	0.1
2.157064	0	3	GZMH	0.1	1.153068	0	3	SUN2	0.1
2.116647	0	3	KLRC1	0.1	1.129567	0	3	ARPC5L	0.1
2.084582	0	3	IL2RB	0.1	1.118969	0	3	MYO1F	0.1
1.945135	0	3	MATK	0.1	1.114755	0	3	PFN1	0.1
1.90326	0	3	IGFBP7	0.1	1.113348	0	3	PRKCH	0.1
1.884007	0	3	IFITM1	0.1	1.10719	0	3	CALM1	0.1
1.819092	0	3	GZMM	0.1	1.100419	0	3	CCND3	0.1
1.679308	0	3	FCGR3A	0.1	1.093941	0	3	IL2RG	0.1
1.649947	0	3	APMAP	0.1	1.092998	0	3	DOK2	0.1
1.633466	0	3	ABHD17A	0.1	1.083508	0	3	TXK	0.1
1.608864	0	3	PRSS23	0.1	1.071902	0	3	DUSP2	0.1
1.564989	0	3	S1PR5	0.1	1.063178	0	3	MYL12A	0.1
1.506852	0	3	ADGRG1	0.1	1.061877	0	3	TNFRSF18	0.1
1.497622	0	3	UBB	0.1	1.041469	0	3	7-Sep	0.1
1.450099	0	3	CHST12	0.1	1.039393	0	3	TBX21	0.1
1.449997	0	3	TTC38	0.1	1.034966	0	3	DHRS7	0.1
1.428553	0	3	C12orf75	0.1	1.034445	0	3	PYHIN1	0.1
1.416956	0	3	DDIT4	0.1	1.029878	0	3	LAIR2	0.1
1.385173	0	3	NCR3	0.1	1.029877	0	3	SAMD3	0.1
1.378179	0	3	HCST	0.1	1.0281	0	3	JAK1	0.1
1.368566	0	3	LITAF	0.1	1.020274	0	3	EVL	0.1
1.361762	0	3	MYOM2	0.1	1.00878	0	3	CEP78	0.1
1.355721	0	3	HLA-C	0.1	1.002539	0	3	HLA-E	0.1
1.347029	0	3	CHST2	0.1	1.001933	0	3	CCL3	0.1
1.343458	0	3	ARL4C	0.1	1.249677	1.92E-248	3	S100B	0.1
1.342579	0	3	ITGB2	0.1					
1.324152	0	3	CD160	0.1					
1.31825	0	3	EFHD2	0.1					
1.315207	0	3	PLAC8	0.1					

Table 11: The cluster biomarkers obtained from differential analysis using Seurat.

**avg_log2FC**	**p_val_adj**	**cluster**	**gene**	**resolution**	**avg_log2FC**	**p_val_adj**	**cluster**	**gene**	**resolution**
4.014435	0	4	GNLY	0.2	1.309567	0	4	FCRL6	0.2
3.468731	0	4	NKG7	0.2	1.288656	0	4	RARRES3	0.2
3.430609	0	4	GZMB	0.2	1.288008	0	4	AKR1C3	0.2
3.325754	0	4	FGFBP2	0.2	1.282302	0	4	CD63	0.2
3.174951	0	4	PRF1	0.2	1.276298	0	4	APOBEC3G	0.2
3.067408	0	4	SPON2	0.2	1.275653	0	4	ID2	0.2
2.834992	0	4	KLRF1	0.2	1.256366	0	4	BIN2	0.2
2.828656	0	4	CST7	0.2	1.234389	0	4	RHOC	0.2
2.824247	0	4	GZMA	0.2	1.232373	0	4	TPST2	0.2
2.805807	0	4	CTSW	0.2	1.220332	0	4	RAC2	0.2
2.68162	0	4	PTGDS	0.2	1.210355	0	4	RAP1B	0.2
2.67781	0	4	HOPX	0.2	1.207149	0	4	SH2D1B	0.2
2.584942	0	4	CLIC3	0.2	1.199539	0	4	RUNX3	0.2
2.473272	0	4	KLRD1	0.2	1.197541	0	4	AREG	0.2
2.453255	0	4	CD7	0.2	1.190336	0	4	C1orf21	0.2
2.412172	0	4	CCL5	0.2	1.172587	0	4	ZAP70	0.2
2.338308	0	4	KLRB1	0.2	1.169826	0	4	IFITM2	0.2
2.330104	0	4	CCL4	0.2	1.169038	0	4	CD99	0.2
2.299494	0	4	TRDC	0.2	1.164231	0	4	PTGDR	0.2
2.240166	0	4	CD247	0.2	1.162668	0	4	XCL2	0.2
2.225809	0	4	CMC1	0.2	1.16065	0	4	HLA-A	0.2
2.156609	0	4	GZMH	0.2	1.151751	0	4	SUN2	0.2
2.112718	0	4	KLRC1	0.2	1.129275	0	4	ARPC5L	0.2
2.084445	0	4	IL2RB	0.2	1.118194	0	4	MYO1F	0.2
1.943916	0	4	MATK	0.2	1.114269	0	4	PFN1	0.2
1.902116	0	4	IGFBP7	0.2	1.113626	0	4	PRKCH	0.2
1.884706	0	4	IFITM1	0.2	1.106507	0	4	CALM1	0.2
1.817867	0	4	GZMM	0.2	1.101224	0	4	CCND3	0.2
1.679639	0	4	FCGR3A	0.2	1.093585	0	4	IL2RG	0.2
1.6504	0	4	APMAP	0.2	1.09059	0	4	DOK2	0.2
1.634224	0	4	ABHD17A	0.2	1.083803	0	4	TXK	0.2
1.608234	0	4	PRSS23	0.2	1.073092	0	4	DUSP2	0.2
1.562282	0	4	S1PR5	0.2	1.062886	0	4	MYL12A	0.2
1.508214	0	4	ADGRG1	0.2	1.059952	0	4	TNFRSF18	0.2
1.497878	0	4	UBB	0.2	1.041908	0	4	7-Sep	0.2
1.45123	0	4	CHST12	0.2	1.040517	0	4	TBX21	0.2
1.450308	0	4	TTC38	0.2	1.035107	0	4	PYHIN1	0.2
1.429036	0	4	C12orf75	0.2	1.034187	0	4	DHRS7	0.2
1.417299	0	4	DDIT4	0.2	1.028931	0	4	JAK1	0.2
1.385263	0	4	NCR3	0.2	1.028863	0	4	LAIR2	0.2
1.378082	0	4	HCST	0.2	1.028178	0	4	SAMD3	0.2
1.368686	0	4	LITAF	0.2	1.020663	0	4	EVL	0.2
1.36272	0	4	MYOM2	0.2	1.009522	0	4	CEP78	0.2
1.355593	0	4	HLA-C	0.2	1.003413	0	4	HLA-E	0.2
1.348295	0	4	CHST2	0.2	1.003238	0	4	CCL3	0.2
1.342703	0	4	ITGB2	0.2	1.249152	1.44E-247	4	S100B	0.2
1.342481	0	4	ARL4C	0.2					
1.324843	0	4	CD160	0.2					
1.318342	0	4	EFHD2	0.2					
1.314735	0	4	PLAC8	0.2					

Table 12: The cluster biomarkers obtained from differential analysis using Seurat.

avg_log2FC	p_val_adj	cluster	gene	resolution	avg_log2FC	p_val_adj	cluster	gene	resolution
4.009375	0	3	GNLY	0.3	1.308919	0	3	FCRL6	0.3
3.464274	0	3	NKG7	0.3	1.289442	0	3	RARRES3	0.3
3.420344	0	3	GZMB	0.3	1.289432	0	3	AKR1C3	0.3
3.324157	0	3	FGFBP2	0.3	1.280773	0	3	CD63	0.3
3.169425	0	3	PRF1	0.3	1.27492	0	3	APOBEC3G	0.3
3.064948	0	3	SPON2	0.3	1.274693	0	3	ID2	0.3
2.834279	0	3	KLRF1	0.3	1.256634	0	3	BIN2	0.3
2.827684	0	3	CST7	0.3	1.233418	0	3	RHOC	0.3
2.816884	0	3	GZMA	0.3	1.232301	0	3	TPST2	0.3
2.80108	0	3	CTSW	0.3	1.217685	0	3	RAC2	0.3
2.685741	0	3	PTGDS	0.3	1.210349	0	3	RAP1B	0.3
2.675788	0	3	HOPX	0.3	1.208389	0	3	SH2D1B	0.3
2.583314	0	3	CLIC3	0.3	1.201228	0	3	RUNX3	0.3
2.473105	0	3	KLRD1	0.3	1.198278	0	3	AREG	0.3
2.451711	0	3	CD7	0.3	1.189391	0	3	C1orf21	0.3
2.409389	0	3	CCL5	0.3	1.171157	0	3	ZAP70	0.3
2.340021	0	3	KLRB1	0.3	1.169076	0	3	IFITM2	0.3
2.328017	0	3	CCL4	0.3	1.166486	0	3	CD99	0.3
2.299204	0	3	TRDC	0.3	1.166249	0	3	PTGDR	0.3
2.237841	0	3	CD247	0.3	1.159828	0	3	HLA-A	0.3
2.22432	0	3	CMC1	0.3	1.158181	0	3	XCL2	0.3
2.152552	0	3	GZMH	0.3	1.151085	0	3	SUN2	0.3
2.110445	0	3	KLRC1	0.3	1.129878	0	3	ARPC5L	0.3
2.085271	0	3	IL2RB	0.3	1.118536	0	3	MYO1F	0.3
1.942961	0	3	MATK	0.3	1.112479	0	3	PRKCH	0.3
1.902735	0	3	IGFBP7	0.3	1.11183	0	3	PFN1	0.3
1.88402	0	3	IFITM1	0.3	1.103635	0	3	CALM1	0.3
1.818345	0	3	GZMM	0.3	1.098647	0	3	CCND3	0.3
1.680333	0	3	FCGR3A	0.3	1.092806	0	3	IL2RG	0.3
1.649176	0	3	APMAP	0.3	1.092153	0	3	DOK2	0.3
1.633953	0	3	ABHD17A	0.3	1.084576	0	3	TXK	0.3
1.608673	0	3	PRSS23	0.3	1.075477	0	3	DUSP2	0.3
1.563245	0	3	S1PR5	0.3	1.063199	0	3	MYL12A	0.3
1.508221	0	3	ADGRG1	0.3	1.060936	0	3	TNFRSF18	0.3
1.49553	0	3	UBB	0.3	1.041119	0	3	TBX21	0.3
1.452673	0	3	CHST12	0.3	1.039591	0	3	7-Sep	0.3
1.447852	0	3	TTC38	0.3	1.035015	0	3	PYHIN1	0.3
1.427217	0	3	C12orf75	0.3	1.034165	0	3	DHRS7	0.3
1.419296	0	3	DDIT4	0.3	1.029965	0	3	JAK1	0.3
1.383587	0	3	NCR3	0.3	1.028897	0	3	LAIR2	0.3
1.378248	0	3	HCST	0.3	1.028506	0	3	SAMD3	0.3
1.369845	0	3	LITAF	0.3	1.022094	0	3	EVL	0.3
1.360883	0	3	MYOM2	0.3	1.010356	0	3	CEP78	0.3
1.354972	0	3	HLA-C	0.3	1.002618	0	3	HLA-E	0.3
1.348861	0	3	CHST2	0.3	1.002461	0	3	CCL3	0.3
1.343242	0	3	ARL4C	0.3	1.251481	3.71E-246	3	S100B	0.3
1.343132	0	3	ITGB2	0.3					
1.32448	0	3	CD160	0.3					
1.31726	0	3	EFHD2	0.3					
1.314231	0	3	PLAC8	0.3					

Table 13: The cluster biomarkers obtained from differential analysis using Seurat.

avg_log2FC	p_val_adj	cluster	gene	resolution	avg_log2FC	p_val_adj	cluster	gene	resolution
4.01018	0	1	GNLY	0.4	1.308053	0	1	FCRL6	0.4
3.464939	0	1	NKG7	0.4	1.288645	0	1	AKR1C3	0.4
3.420244	0	1	GZMB	0.4	1.288231	0	1	RARRES3	0.4
3.323293	0	1	FGFBP2	0.4	1.279798	0	1	CD63	0.4
3.168127	0	1	PRF1	0.4	1.273934	0	1	APOBEC3G	0.4
3.063716	0	1	SPON2	0.4	1.273402	0	1	ID2	0.4
2.834236	0	1	KLRF1	0.4	1.25567	0	1	BIN2	0.4
2.827499	0	1	CST7	0.4	1.233843	0	1	RHOC	0.4
2.816065	0	1	GZMA	0.4	1.23287	0	1	TPST2	0.4
2.80118	0	1	CTSW	0.4	1.216696	0	1	RAC2	0.4
2.684586	0	1	PTGDS	0.4	1.209634	0	1	RAP1B	0.4
2.675352	0	1	HOPX	0.4	1.20962	0	1	SH2D1B	0.4
2.582168	0	1	CLIC3	0.4	1.200805	0	1	RUNX3	0.4
2.473671	0	1	KLRD1	0.4	1.197296	0	1	AREG	0.4
2.451858	0	1	CD7	0.4	1.188586	0	1	C1orf21	0.4
2.409439	0	1	CCL5	0.4	1.172282	0	1	ZAP70	0.4
2.339113	0	1	KLRB1	0.4	1.169586	0	1	IFITM2	0.4
2.326818	0	1	CCL4	0.4	1.166514	0	1	CD99	0.4
2.30052	0	1	TRDC	0.4	1.166257	0	1	PTGDR	0.4
2.236632	0	1	CD247	0.4	1.159351	0	1	HLA-A	0.4
2.226722	0	1	CMC1	0.4	1.158054	0	1	XCL2	0.4
2.151804	0	1	GZMH	0.4	1.152621	0	1	SUN2	0.4
2.114663	0	1	KLRC1	0.4	1.128909	0	1	ARPC5L	0.4
2.084998	0	1	IL2RB	0.4	1.11954	0	1	MYO1F	0.4
1.944061	0	1	MATK	0.4	1.111522	0	1	PRKCH	0.4
1.903479	0	1	IGFBP7	0.4	1.111263	0	1	PFN1	0.4
1.884518	0	1	IFITM1	0.4	1.102961	0	1	CALM1	0.4
1.818902	0	1	GZMM	0.4	1.098246	0	1	CCND3	0.4
1.679087	0	1	FCGR3A	0.4	1.094219	0	1	DOK2	0.4
1.64812	0	1	APMAP	0.4	1.093076	0	1	IL2RG	0.4
1.63359	0	1	ABHD17A	0.4	1.084757	0	1	TXK	0.4
1.609282	0	1	PRSS23	0.4	1.074319	0	1	DUSP2	0.4
1.565978	0	1	S1PR5	0.4	1.062597	0	1	MYL12A	0.4
1.507309	0	1	ADGRG1	0.4	1.061031	0	1	TNFRSF18	0.4
1.494889	0	1	UBB	0.4	1.040366	0	1	TBX21	0.4
1.451637	0	1	CHST12	0.4	1.038985	0	1	7-Sep	0.4
1.446977	0	1	TTC38	0.4	1.034439	0	1	DHRS7	0.4
1.426566	0	1	C12orf75	0.4	1.034187	0	1	PYHIN1	0.4
1.419496	0	1	DDIT4	0.4	1.030279	0	1	SAMD3	0.4
1.383659	0	1	NCR3	0.4	1.030272	0	1	LAIR2	0.4
1.378121	0	1	HCST	0.4	1.029283	0	1	JAK1	0.4
1.370744	0	1	LITAF	0.4	1.02191	0	1	EVL	0.4
1.360072	0	1	MYOM2	0.4	1.009611	0	1	CEP78	0.4
1.354893	0	1	HLA-C	0.4	1.001866	0	1	HLA-E	0.4
1.348013	0	1	CHST2	0.4	1.001588	0	1	CCL3	0.4
1.344507	0	1	ARL4C	0.4	1.250658	8.34E-246	1	S100B	0.4

Table 14: The cluster biomarkers obtained from differential analysis using Seurat.

avg_log2FC	p_val_adj	cluster	gene	resolution	avg_log2FC	p_val_adj	cluster	gene	resolution
4.00902	0	1	GNLY	0.5	1.308922	0	1	FCRL6	0.5
3.463192	0	1	NKG7	0.5	1.288657	0	1	AKR1C3	0.5
3.416775	0	1	GZMB	0.5	1.288514	0	1	RARRES3	0.5
3.324105	0	1	FGFBP2	0.5	1.28038	0	1	CD63	0.5
3.168534	0	1	PRF1	0.5	1.274068	0	1	ID2	0.5
3.062797	0	1	SPON2	0.5	1.273321	0	1	APOBEC3G	0.5
2.833674	0	1	KLRF1	0.5	1.256541	0	1	BIN2	0.5
2.827345	0	1	CST7	0.5	1.233012	0	1	TPST2	0.5
2.814432	0	1	GZMA	0.5	1.232238	0	1	RHOC	0.5
2.800321	0	1	CTSW	0.5	1.217515	0	1	RAC2	0.5
2.68603	0	1	PTGDS	0.5	1.210481	0	1	RAP1B	0.5
2.675296	0	1	HOPX	0.5	1.205996	0	1	SH2D1B	0.5
2.58182	0	1	CLIC3	0.5	1.201306	0	1	RUNX3	0.5
2.47281	0	1	KLRD1	0.5	1.194494	0	1	AREG	0.5
2.45142	0	1	CD7	0.5	1.189105	0	1	C1orf21	0.5
2.409099	0	1	CCL5	0.5	1.171101	0	1	ZAP70	0.5
2.33982	0	1	KLRB1	0.5	1.16941	0	1	IFITM2	0.5
2.326928	0	1	CCL4	0.5	1.166575	0	1	CD99	0.5
2.298882	0	1	TRDC	0.5	1.166347	0	1	PTGDR	0.5
2.236147	0	1	CD247	0.5	1.159929	0	1	HLA-A	0.5
2.224639	0	1	CMC1	0.5	1.158762	0	1	XCL2	0.5
2.151145	0	1	GZMH	0.5	1.151425	0	1	SUN2	0.5
2.1103	0	1	KLRC1	0.5	1.128919	0	1	ARPC5L	0.5
2.084355	0	1	IL2RB	0.5	1.118465	0	1	MYO1F	0.5
1.943078	0	1	MATK	0.5	1.112163	0	1	PRKCH	0.5
1.902584	0	1	IGFBP7	0.5	1.111322	0	1	PFN1	0.5
1.882606	0	1	IFITM1	0.5	1.102503	0	1	CALM1	0.5
1.819135	0	1	GZMM	0.5	1.098706	0	1	CCND3	0.5
1.67966	0	1	FCGR3A	0.5	1.092692	0	1	IL2RG	0.5
1.648438	0	1	APMAP	0.5	1.091327	0	1	DOK2	0.5
1.634635	0	1	ABHD17A	0.5	1.085225	0	1	TXK	0.5
1.608491	0	1	PRSS23	0.5	1.074087	0	1	DUSP2	0.5
1.563477	0	1	S1PR5	0.5	1.062994	0	1	MYL12A	0.5
1.508449	0	1	ADGRG1	0.5	1.061959	0	1	TNFRSF18	0.5
1.493573	0	1	UBB	0.5	1.040905	0	1	TBX21	0.5
1.450624	0	1	CHST12	0.5	1.039595	0	1	7-Sep	0.5
1.447649	0	1	TTC38	0.5	1.035223	0	1	PYHIN1	0.5
1.427235	0	1	C12orf75	0.5	1.033483	0	1	DHRS7	0.5
1.419677	0	1	DDIT4	0.5	1.02864	0	1	JAK1	0.5
1.384812	0	1	NCR3	0.5	1.028345	0	1	SAMD3	0.5
1.378598	0	1	HCST	0.5	1.028238	0	1	LAIR2	0.5
1.369821	0	1	LITAF	0.5	1.022126	0	1	EVL	0.5
1.36035	0	1	MYOM2	0.5	1.009364	0	1	CEP78	0.5
1.355017	0	1	HLA-C	0.5	1.00268	0	1	CCL3	0.5
1.347922	0	1	CHST2	0.5	1.002533	0	1	HLA-E	0.5
1.343176	0	1	ITGB2	0.5	1.251687	3.03E-246	1	S100B	0.5
1.342647	0	1	ARL4C	0.5					
1.324	0	1	CD160	0.5					
1.317065	0	1	EFHD2	0.5					
1.313002	0	1	PLAC8	0.5					

Table 15: The cluster biomarkers obtained from differential analysis using Seurat.

avg_log2FC	p_val_adj	cluster	gene	resolution	avg_log2FC	p_val_adj	cluster	gene	resolution
1.763141	2.54E-189	0	FGFBP2	0.6	1.819116	9.46E-193	0	FGFBP2	0.9
1.499765	1.77E-121	0	SPON2	0.6	1.515168	7.56E-121	0	SPON2	0.9
1.073317	2.45E-108	0	PRF1	0.6	1.088978	6.27E-109	0	PRF1	0.9
1.099332	1.66E-83	0	FCGR3A	0.6	1.003974	6.79E-94	0	S100A4	0.9
1.075723	2.33E-41	0	LGALS1	0.6	1.103839	6.09E-83	0	FCGR3A	0.9
1.046997	7.91E-41	0	IGFBP7	0.6	1.077201	8.97E-41	0	LGALS1	0.9
2.724291	1.13E-32	0	PTGDS	0.6	1.043016	7.73E-40	0	IGFBP7	0.9
1.003364	4.44E-12	0	MYOM2	0.6	2.848082	5.59E-33	0	PTGDS	0.9
1.856074	2.62E-217	1	GZMK	0.6	1.025457	6.18E-12	0	MYOM2	0.9
1.221811	4.34E-164	1	XCL1	0.6	1.875078	2.86E-222	1	GZMK	0.9
1.253122	9.90E-111	1	XCL2	0.6	1.236997	6.99E-168	1	XCL1	0.9
1.206908	3.92E-84	1	SELL	0.6	1.25859	3.59E-111	1	XCL2	0.9
1.161844	1.53E-65	1	COTL1	0.6	1.192667	2.81E-83	1	SELL	0.9
1.020933	4.96E-65	1	CD44	0.6	1.179079	5.96E-68	1	COTL1	0.9
1.070541	5.91E-57	1	DUSP2	0.6	1.037145	5.67E-67	1	CD44	0.9
1.014145	6.44E-52	1	NFKBIA	0.6	1.065615	1.86E-56	1	DUSP2	0.9
1.061849	1.85E-40	1	FOS	0.6	1.034527	4.61E-40	1	FOS	0.9
1.861679	4.15E-193	0	FGFBP2	0.7	1.817763	1.23E-189	0	FGFBP2	1.0
1.52649	2.89E-119	0	SPON2	0.7	1.537082	4.68E-122	0	SPON2	1.0
1.090834	7.01E-107	0	PRF1	0.7	1.093236	2.23E-108	0	PRF1	1.0
1.012818	3.00E-92	0	S100A4	0.7	1.004555	8.93E-93	0	S100A4	1.0
1.098229	1.68E-80	0	FCGR3A	0.7	1.094489	4.69E-81	0	FCGR3A	1.0
1.071061	4.19E-39	0	LGALS1	0.7	1.06859	8.13E-40	0	LGALS1	1.0
1.038729	1.14E-38	0	IGFBP7	0.7	1.032116	1.77E-38	0	IGFBP7	1.0
2.842396	3.38E-32	0	PTGDS	0.7	2.891955	7.74E-33	0	PTGDS	1.0
1.050175	2.91E-12	0	MYOM2	0.7	1.026337	7.68E-12	0	MYOM2	1.0
1.898262	1.89E-227	1	GZMK	0.7	1.891157	2.09E-226	1	GZMK	1.0
1.235692	2.96E-171	1	XCL1	0.7	1.249881	4.85E-171	1	XCL1	1.0
1.27857	8.88E-114	1	XCL2	0.7	1.271826	1.56E-113	1	XCL2	1.0
1.215856	9.58E-86	1	SELL	0.7	1.211259	2.57E-86	1	SELL	1.0
1.046658	1.40E-69	1	CD44	0.7	1.193514	1.34E-69	1	COTL1	1.0
1.191251	5.35E-68	1	COTL1	0.7	1.048848	3.09E-68	1	CD44	1.0
1.083411	1.33E-57	1	DUSP2	0.7	1.076008	1.91E-57	1	DUSP2	1.0
1.038374	9.22E-40	1	FOS	0.7	1.034451	1.28E-40	1	FOS	1.0
1.861679	4.15E-193	0	FGFBP2	0.8	1.539667	1.29E-231	0	SPON2	1.1
1.52649	2.89E-119	0	SPON2	0.8	1.079363	8.49E-203	0	PRF1	1.1
1.090834	7.01E-107	0	PRF1	0.8	1.175233	2.72E-190	0	FCER1G	1.1
1.012818	3.00E-92	0	S100A4	0.8	1.231215	1.87E-186	0	FCGR3A	1.1
1.098229	1.68E-80	0	FCGR3A	0.8	1.051725	6.32E-180	0	FGFBP2	1.1
1.071061	4.19E-39	0	LGALS1	0.8	1.280813	1.13E-120	0	IGFBP7	1.1
1.038729	1.14E-38	0	IGFBP7	0.8	1.022603	5.65E-70	0	AKR1C3	1.1
2.842396	3.38E-32	0	PTGDS	0.8	2.422906	7.64E-48	0	PTGDS	1.1
1.050175	2.91E-12	0	MYOM2	0.8	1.185787	1.47E-40	0	MYOM2	1.1
1.898262	1.89E-227	1	GZMK	0.8	2.469914	0	1	CD3D	1.1
1.235692	2.96E-171	1	XCL1	0.8	1.566999	4.81E-277	1	CD3G	1.1
1.27857	8.88E-114	1	XCL2	0.8	1.612389	8.27E-238	1	CD52	1.1
1.215856	9.58E-86	1	SELL	0.8	1.675221	5.13E-207	1	IL7R	1.1
1.046658	1.40E-69	1	CD44	0.8	1.630126	1.46E-199	1	TRAC	1.1
1.191251	5.35E-68	1	COTL1	0.8	1.236421	2.31E-151	1	CD3E	1.1
1.083411	1.33E-57	1	DUSP2	0.8	1.070068	8.22E-112	1	IL32	1.1
1.038374	9.22E-40	1	FOS	0.8	1.151038	1.65E-39	1	TRGC1	1.1

Table 16: The cluster biomarkers obtained from differential analysis using Seurat.

avg_log2FC	p_val_adj	cluster	gene	resolution	avg_log2FC	p_val_adj	cluster	gene	resolution
2.037642	1.69E-267	2	GZMK	1.1	1.50094	3.98E-215	0	SPON2	1.4
1.359538	3.75E-211	2	XCL1	1.1	1.078855	1.46E-195	0	PRF1	1.4
1.371353	2.52E-118	2	XCL2	1.1	1.217287	9.31E-192	0	FCER1G	1.4
1.342924	4.89E-94	2	SELL	1.1	1.246124	1.20E-182	0	FCGR3A	1.4
1.29336	5.01E-80	2	COTL1	1.1	1.00578	2.15E-159	0	FGFBP2	1.4
1.021831	2.61E-60	2	CD44	1.1	1.287582	6.61E-117	0	IGFBP7	1.4
1.115556	4.23E-53	2	DUSP2	1.1	2.336047	7.01E-43	0	PTGDS	1.4
1.018369	2.70E-42	2	NFKBIA	1.1	1.163225	6.63E-38	0	MYOM2	1.4
1.535268	1.57E-223	0	SPON2	1.2	2.467952	0	1	CD3D	1.4
1.089213	4.31E-201	0	PRF1	1.2	1.56377	1.38E-270	1	CD3G	1.4
1.217377	5.08E-195	0	FCER1G	1.2	1.598196	2.32E-232	1	CD52	1.4
1.256035	1.45E-186	0	FCGR3A	1.2	1.660777	4.76E-200	1	IL7R	1.4
1.036468	5.88E-169	0	FGFBP2	1.2	1.610527	1.45E-190	1	TRAC	1.4
1.296495	2.73E-120	0	IGFBP7	1.2	1.217555	3.15E-144	1	CD3E	1.4
1.007975	7.22E-73	0	CHST2	1.2	1.069865	2.40E-110	1	IL32	1.4
1.014707	4.88E-67	0	AKR1C3	1.2	1.144008	5.60E-38	1	TRGC1	1.4
2.405128	4.61E-45	0	PTGDS	1.2	2.090298	8.03E-263	2	GZMK	1.4
1.196911	3.48E-40	0	MYOM2	1.2	1.411646	1.37E-216	2	XCL1	1.4
2.46374	0	1	CD3D	1.2	1.388517	1.52E-109	2	XCL2	1.4
1.568059	4.40E-277	1	CD3G	1.2	1.393837	1.51E-92	2	SELL	1.4
1.614893	5.24E-239	1	CD52	1.2	1.327646	3.80E-74	2	COTL1	1.4
1.687443	4.64E-208	1	IL7R	1.2	1.097007	4.84E-63	2	CD44	1.4
1.620049	3.40E-196	1	TRAC	1.2	1.101467	2.43E-45	2	DUSP2	1.4
1.224254	8.43E-148	1	CD3E	1.2	1.079838	8.29E-43	2	NFKBIA	1.4
1.073922	7.93E-113	1	IL32	1.2	1.061573	2.01E-31	2	FOS	1.4
1.148773	9.95E-39	1	TRGC1	1.2	1.533742	1.65E-224	0	SPON2	1.5
2.091059	1.99E-264	2	GZMK	1.2	1.080961	1.58E-199	0	PRF1	1.5
1.389902	2.90E-211	2	XCL1	1.2	1.177726	1.28E-186	0	FCER1G	1.5
1.368701	2.23E-110	2	XCL2	1.2	1.249414	1.55E-186	0	FCGR3A	1.5
1.372218	2.61E-92	2	SELL	1.2	1.050454	1.03E-175	0	FGFBP2	1.5
1.315748	5.82E-75	2	COTL1	1.2	1.294247	4.69E-120	0	IGFBP7	1.5
1.064626	1.53E-59	2	CD44	1.2	1.013145	1.85E-67	0	AKR1C3	1.5
1.113163	3.20E-47	2	DUSP2	1.2	2.392894	3.14E-45	0	PTGDS	1.5
1.057521	9.85E-42	2	NFKBIA	1.2	1.168325	5.29E-38	0	MYOM2	1.5
1.044352	6.10E-31	2	FOS	1.2	2.470231	0	1	CD3D	1.5
1.824559	6.96E-187	0	FGFBP2	1.3	1.568653	3.55E-275	1	CD3G	1.5
1.543219	1.68E-120	0	SPON2	1.3	1.610876	8.36E-239	1	CD52	1.5
1.084896	6.39E-105	0	PRF1	1.3	1.675823	1.15E-205	1	IL7R	1.5
1.076778	7.92E-78	0	FCGR3A	1.3	1.628582	1.38E-196	1	TRAC	1.5
1.048862	5.75E-38	0	LGALS1	1.3	1.224249	2.20E-147	1	CD3E	1.5
1.03393	8.45E-38	0	IGFBP7	1.3	1.074633	1.62E-111	1	IL32	1.5
2.887823	2.63E-32	0	PTGDS	1.3	1.166489	3.22E-41	1	TRGC1	1.5
1.024683	6.84E-12	0	MYOM2	1.3	2.05877	6.03E-270	2	GZMK	1.5
1.904172	4.37E-229	1	GZMK	1.3	1.389915	3.96E-217	2	XCL1	1.5
1.266023	1.13E-175	1	XCL1	1.3	1.395137	7.82E-120	2	XCL2	1.5
1.284988	2.00E-113	1	XCL2	1.3	1.38843	6.88E-98	2	SELL	1.5
1.222931	9.52E-86	1	SELL	1.3	1.312796	3.25E-80	2	COTL1	1.5
1.206464	1.50E-71	1	COTL1	1.3	1.042767	1.37E-60	2	CD44	1.5
1.05957	2.87E-69	1	CD44	1.3	1.086913	7.89E-47	2	DUSP2	1.5
1.084251	4.89E-57	1	DUSP2	1.3	1.0549	1.41E-44	2	NFKBIA	1.5
1.021757	2.41E-39	1	FOS	1.3	1.027335	4.18E-32	2	FOS	1.5

Table 17: The cluster biomarkers obtained from differential analysis using Seurat.

avg_log2FC	p_val_adj	cluster	gene	resolution	avg_log2FC	p_val_adj	cluster	gene	resolution
1.529714	7.88E-179	0	SPON2	1.6	1.05621	8.06E-108	1	IL32	1.8
1.004558	8.35E-145	0	PRF1	1.6	1.156379	4.95E-40	1	TRGC1	1.8
1.179649	2.11E-138	0	FCGR3A	1.6	2.031762	2.24E-264	2	GZMK	1.8
1.021357	2.83E-131	0	FGFBP2	1.6	1.357572	3.87E-209	2	XCL1	1.8
1.018191	8.94E-119	0	FCER1G	1.6	1.376752	9.43E-118	2	XCL2	1.8
1.172615	2.85E-80	0	IGFBP7	1.6	1.355472	8.17E-96	2	SELL	1.8
2.396037	1.02E-32	0	PTGDS	1.6	1.273925	3.20E-77	2	COTL1	1.8
1.122053	3.08E-26	0	MYOM2	1.6	1.023574	6.57E-60	2	CD44	1.8
2.471976	0	1	CD3D	1.6	1.11295	8.45E-53	2	DUSP2	1.8
1.550403	3.96E-219	1	CD3G	1.6	1.016251	1.15E-41	2	NFKBIA	1.8
1.870799	2.13E-197	1	TRAC	1.6	1.258604	1.02E-172	0	SPON2	1.9
1.676912	2.61E-163	1	CD52	1.6	2.279692	1.46E-57	0	PTGDS	1.9
1.589959	9.85E-135	1	IL7R	1.6	1.080334	1.59E-30	0	MYOM2	1.9
1.18368	5.71E-92	1	CD8B	1.6	1.003925	2.50E-65	1	KLRC1	1.9
1.157622	1.84E-87	1	CD3E	1.6	2.614305	9.62E-258	2	GZMK	1.9
1.063329	1.02E-68	1	IL32	1.6	1.272695	1.58E-204	2	SPTSSB	1.9
1.153799	7.33E-52	1	CD8A	1.6	1.889826	1.28E-193	2	XCL1	1.9
1.051192	5.71E-50	1	VIM	1.6	1.273313	5.84E-165	2	CAPG	1.9
2.116645	6.43E-278	2	GZMK	1.6	1.813391	4.05E-79	2	COTL1	1.9
1.388337	2.65E-200	2	XCL1	1.6	1.890936	5.17E-79	2	SELL	1.9
1.331352	6.17E-98	2	XCL2	1.6	1.451269	8.89E-77	2	IL7R	1.9
1.384918	1.51E-90	2	SELL	1.6	1.559418	1.01E-60	2	CD44	1.9
1.303309	3.39E-69	2	COTL1	1.6	1.471313	1.19E-59	2	XCL2	1.9
1.121999	3.74E-66	2	CD44	1.6	1.494477	9.89E-38	2	LTB	1.9
1.103123	6.66E-45	2	DUSP2	1.6	1.063533	7.43E-23	2	IFITM3	1.9
1.039857	3.20E-38	2	NFKBIA	1.6	1.129001	2.42E-20	2	NFKBIA	1.9
1.09528	1.36E-31	2	FOS	1.6	1.101871	2.37E-19	2	AREG	1.9
1.214719	1.91E-163	0	SPON2	1.7	1.164882	8.67E-17	2	DUSP2	1.9
2.261687	1.43E-57	0	PTGDS	1.7	1.529531	1.48E-218	0	SPON2	2.0
1.005994	9.18E-26	0	MYOM2	1.7	1.076563	1.38E-194	0	PRF1	2.0
1.042864	8.17E-41	1	KLRC1	1.7	1.224098	1.07E-192	0	FCER1G	2.0
1.767637	2.34E-202	2	GZMK	1.7	1.226467	5.32E-178	0	FCGR3A	2.0
1.236799	4.18E-162	2	XCL1	1.7	1.020275	2.73E-162	0	FGFBP2	2.0
1.268753	8.31E-110	2	XCL2	1.7	1.295275	1.60E-117	0	IGFBP7	2.0
1.27817	1.65E-95	2	SELL	1.7	1.008501	1.89E-65	0	AKR1C3	2.0
1.166743	7.38E-62	2	COTL1	1.7	2.393842	7.36E-44	0	PTGDS	2.0
1.049085	7.70E-59	2	NFKBIA	1.7	1.182183	2.88E-38	0	MYOM2	2.0
1.003953	3.71E-48	2	DUSP2	1.7	2.480162	0	1	CD3D	2.0
1.015623	2.72E-37	2	FOS	1.7	1.574892	1.20E-274	1	CD3G	2.0
1.524929	1.09E-226	0	SPON2	1.8	1.613155	3.34E-237	1	CD52	2.0
1.077117	1.71E-201	0	PRF1	1.8	1.671521	1.90E-201	1	IL7R	2.0
1.162552	4.08E-187	0	FCER1G	1.8	1.639521	3.38E-198	1	TRAC	2.0
1.225499	4.93E-184	0	FCGR3A	1.8	1.233177	1.91E-148	1	CD3E	2.0
1.043064	4.22E-176	0	FGFBP2	1.8	1.075875	6.99E-111	1	IL32	2.0
1.269321	8.96E-119	0	IGFBP7	1.8	1.173087	4.57E-41	1	TRGC1	2.0
1.005145	1.10E-68	0	AKR1C3	1.8	2.057301	1.79E-253	2	GZMK	2.0
2.400655	6.09E-47	0	PTGDS	1.8	1.381876	2.98E-208	2	XCL1	2.0
1.183508	4.49E-40	0	MYOM2	1.8	1.356495	1.36E-105	2	XCL2	2.0
2.439286	0	1	CD3D	1.8	1.363325	9.51E-90	2	SELL	2.0
1.545066	5.37E-265	1	CD3G	1.8	1.297546	5.97E-71	2	COTL1	2.0
1.588645	1.32E-231	1	CD52	1.8	1.055739	8.53E-59	2	CD44	2.0
1.665522	1.08E-200	1	IL7R	1.8	1.084869	2.72E-46	2	DUSP2	2.0
1.596137	1.12E-191	1	TRAC	1.8	1.0447	1.25E-38	2	NFKBIA	2.0
1.221494	8.98E-146	1	CD3E	1.8	1.043908	3.74E-30	2	FOS	2.0

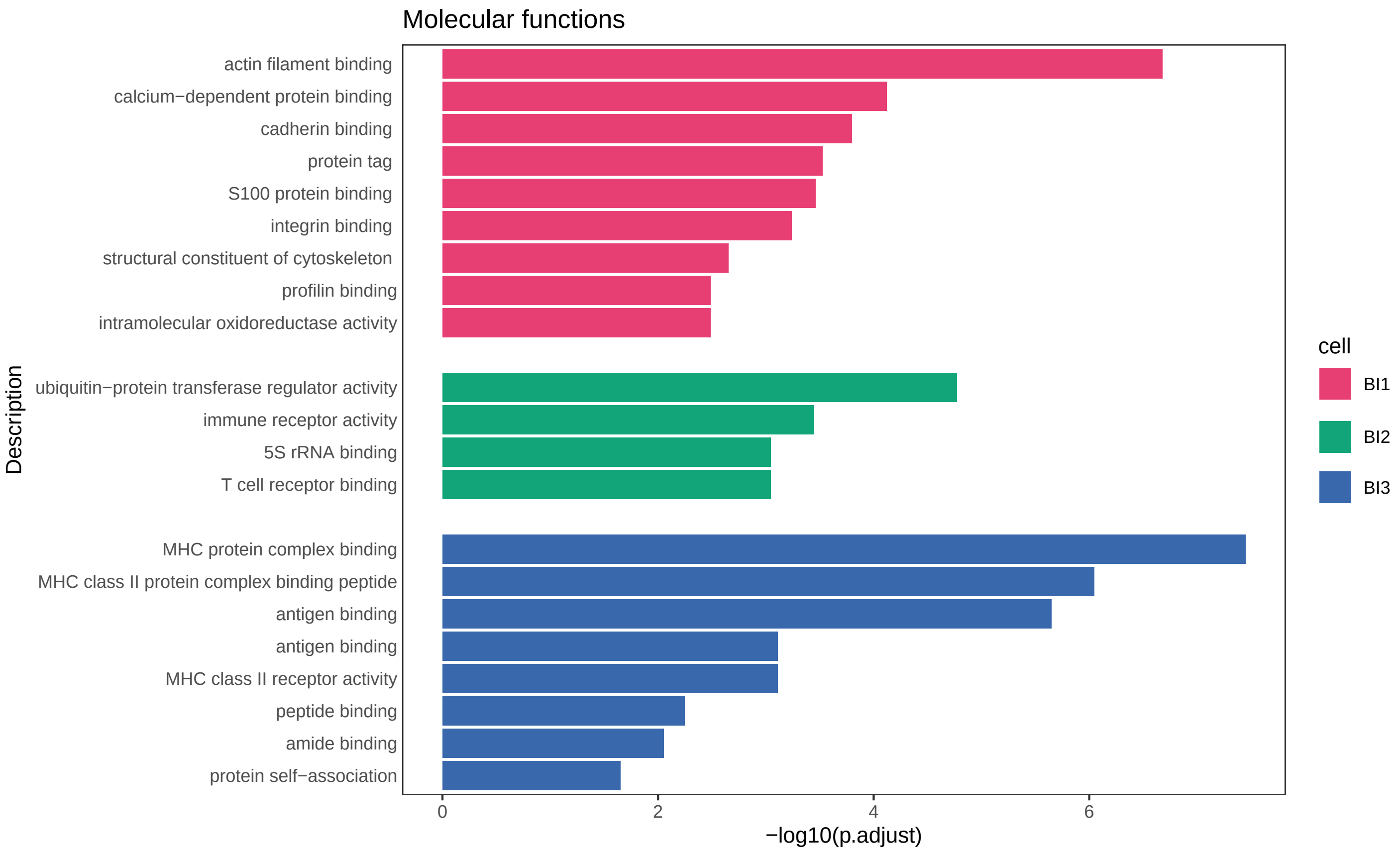

Figure 28: Top enriched Gene Ontology (GO) terms related to distinct cell subsets in terms of molecular function.

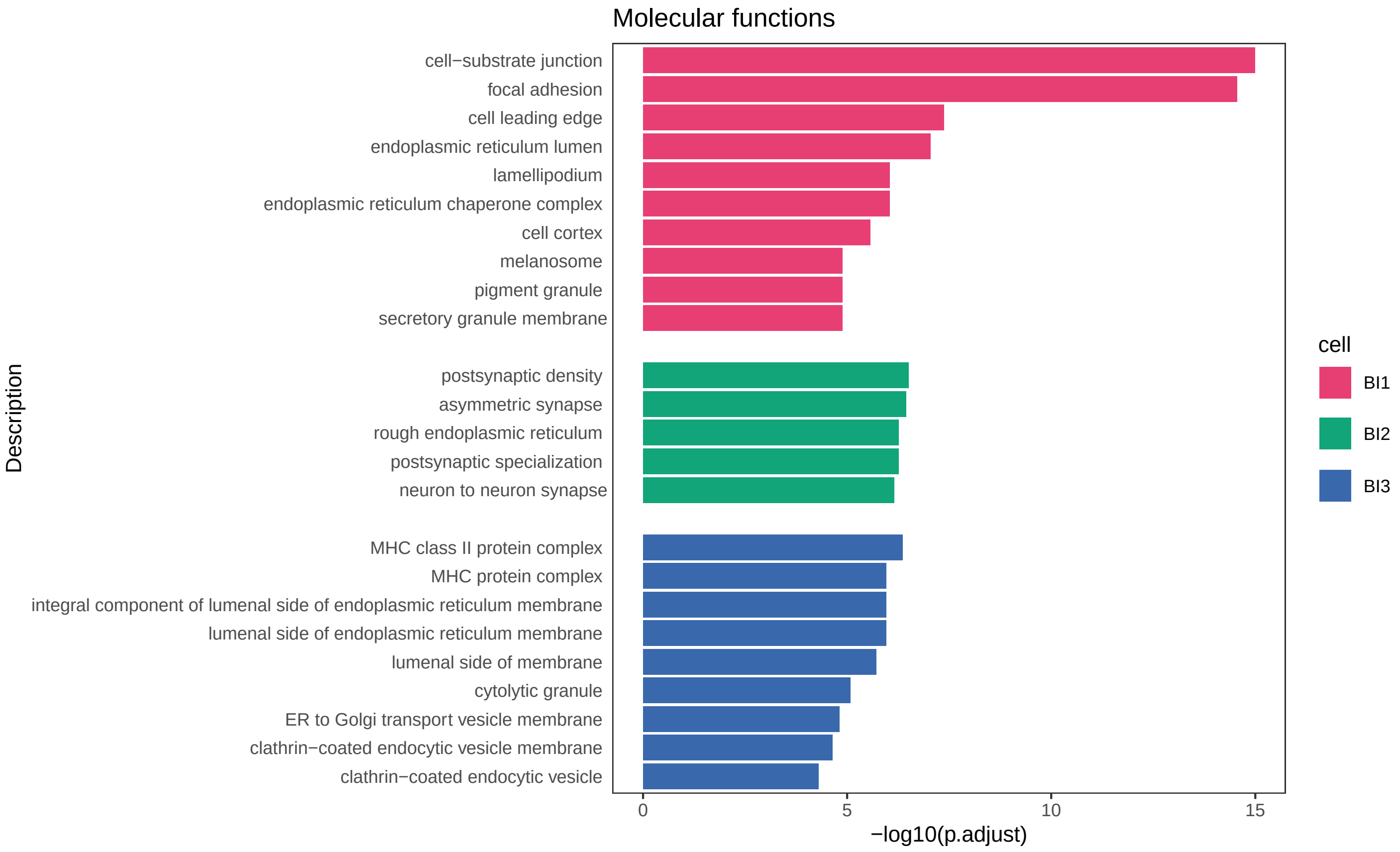

Figure 29: Top enriched Gene Ontology (GO) terms related to distinct cell subsets in terms of cellular components.

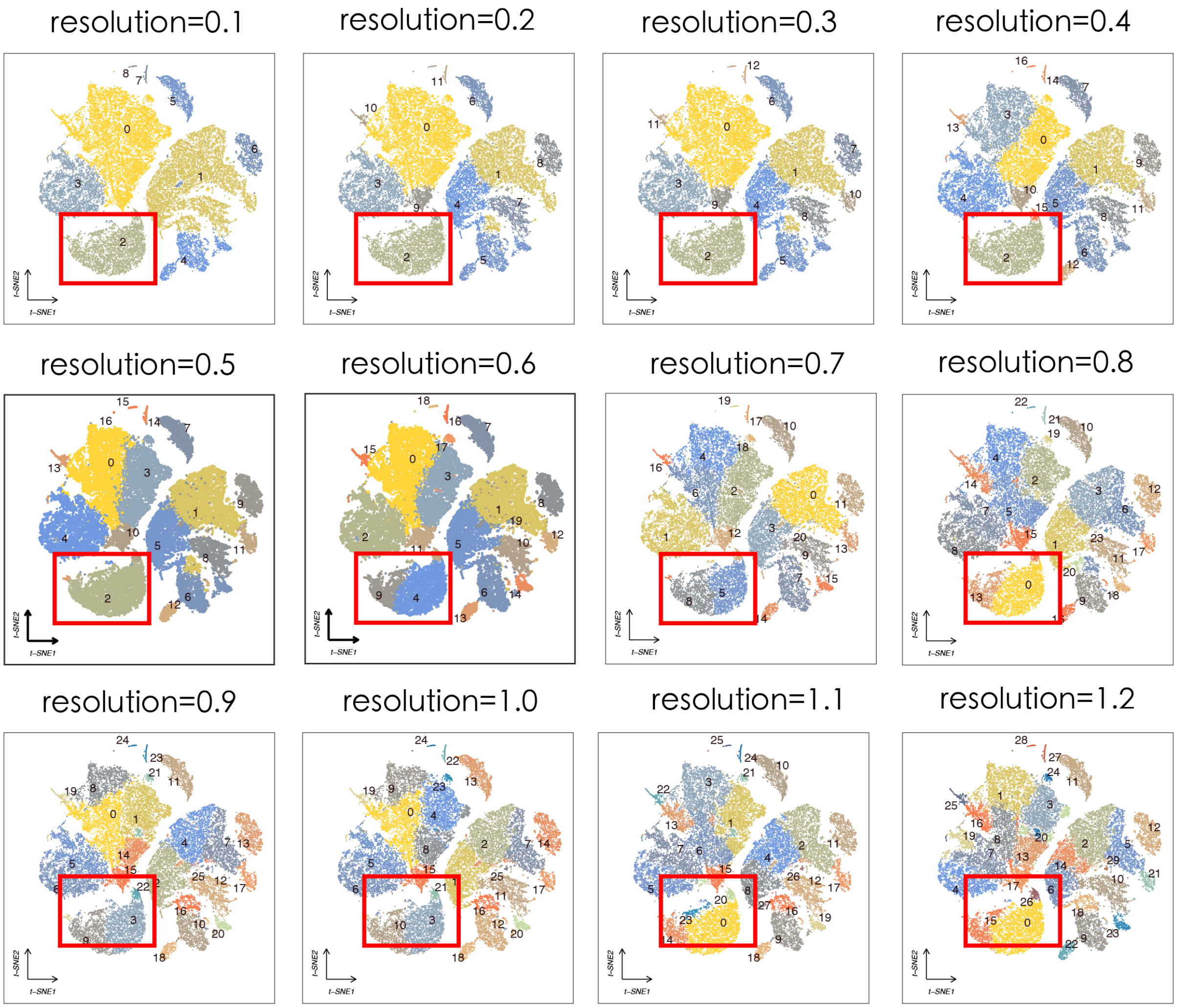

Figure 30: t-SNE plots visualizing the peripheral blood dataset after Bis imputation colored by cell clusters with different resolutions.

Table 18: The cluster biomarkers obtained from Bis-imputed data using Seurat.

avg_log2FC	p_val_adj	cluster	gene	resolution	avg_log2FC	p_val_adj	cluster	gene	resolution
1.282258	0	2	TNFRSF18	0.1	1.339937	0	2	TNFRSF18	0.2
1.814899	0	2	MIB2	0.1	1.863843	0	2	MIB2	0.2
2.16658	0	2	MMP23B	0.1	2.205396	0	2	MMP23B	0.2
-88.2172	0	2	RPL22	0.1	-88.1457	0	2	RPL22	0.2
-11.4185	0	2	RBP7	0.1	-11.4058	0	2	RBP7	0.2
-2.96195	0	2	PGD	0.1	-2.9323	0	2	PGD	0.2
1.091827	0	2	FBXO6	0.1	1.140114	0	2	FBXO6	0.2
1.09721	0	2	DHRS3	0.1	-88.1384	0	2	COTL1	0.2
1.514654	0	2	EFHD2	0.1	-16.6651	0	2	CD44	0.2
-3.32338	0	2	CDA	0.1	3.408495	0	2	XCL1	0.2
-49.9625	0	2	CCL5	0.1	-16.5835	9.74E-10	2	GZMK	0.2
-296.477	0.013658	2	HLA-DPA1	0.1	-40.1535	0	2	FCGR3A	0.2
-186.168	0	2	RPL11	0.1	25.52639	0	2	PRF1	0.2
-17.0769	0	2	GZMH	0.1	4.133381	0	2	IGFBP7	0.2
1.993874	0	2	RUNX3	0.1	10.20446	0	2	CST7	0.2
-19.9452	0	2	STMN1	0.1	15.79909	0	2	SPON2	0.2
-141.671	0	2	CD52	0.1	-17.0054	0	2	GZMH	0.2
15.72819	0	2	SPON2	0.1	-2.84767	0	2	GZMB	0.2
1.444759	0	2	CCDC28B	0.1	37.5874	0	2	FGFBP2	0.2
-8.05335	0	2	LCK	0.1	-49.891	0	2	CCL5	0.2
-2.09494	0	2	AK2	0.1	-344.996	1	2	HLA-DPB1	0.2
-1.67312	0	2	PHC2	0.1	-5.74115	0	2	CSF3R	0.2
1.577396	0	2	AC004865.2	0.1	1.016271	0	2	FHL3	0.2
-5.75115	0	2	CSF3R	0.1	-5.89268	0	2	PABPC4	0.2
-5.92632	0	2	PABPC4	0.1	-8.94316	0	2	PPT1	0.2
10.13304	0	2	CST7	0.1	-3.17675	0	2	SMAP2	0.2
-3.22218	0	2	SMAP2	0.1	-1.02945	0	2	CITED4	0.2
-1.04494	0	2	CITED4	0.1	-9.39028	0	2	EBNA1BP2	0.2
-1.92467	0	2	ARMH1	0.1	-1.91669	0	2	ARMH1	0.2
-158.914	0	2	RPS8	0.1	-158.842	0	2	RPS8	0.2
4.080785	0	2	IGFBP7	0.1	-1.55319	0	2	PLK3	0.2
-15.7822	0	2	NASP	0.1	-15.7177	0	2	NASP	0.2
-2.91919	0	2	GZMB	0.1	-1.45058	0	2	USP1	0.2
25.46086	0	2	PRF1	0.1	2.607434	0	2	JAK1	0.2
-1.08505	0	2	PDE4B	0.1	-1.06375	0	2	PDE4B	0.2
37.51595	0	2	FGFBP2	0.1	-8.00564	0	2	AC103591.3	0.2
-1.11465	0	2	IFI44L	0.1	-1.05348	0	2	IFI44L	0.2
3.344678	0	2	XCL1	0.1	3.526227	0	2	GBP5	0.2
1.847111	0	2	TGFBR3	0.1	1.854586	0	2	TGFBR3	0.2
-1.26296	0	2	EVI5	0.1	-1.25815	0	2	EVI5	0.2
1.355396	0	2	VAV3	0.1	1.392049	0	2	VAV3	0.2
-40.2245	0	2	FCGR3A	0.1	1.309039	0	2	HENMT1	0.2
-1.40089	0	2	SORT1	0.1	-1.02889	0	2	PRPF38B	0.2
-20.3861	0	2	PSMA5	0.1	-1.39452	0	2	SORT1	0.2
-1.03516	0	2	AHCYL1	0.1	-20.3485	0	2	PSMA5	0.2
-1.62511	0	2	DRAM2	0.1	-1.02168	0	2	AHCYL1	0.2
-16.7347	0	2	CD44	0.1	-22.7377	0	2	CD53	0.2
-13.7986	0	2	RHOC	0.1	-1.58705	0	2	DRAM2	0.2
-16.6551	3.28E-11	2	GZMK	0.1	1.200498	0	2	DENND2D	0.2
-88.2099	0	2	COTL1	0.1	-13.7301	0	2	RHOC	0.2

Table 19: The cluster biomarkers obtained from Bis-imputed data using Seurat.

avg_log2FC	p_val_adj	cluster	gene	resolution	avg_log2FC	p_val_adj	cluster	gene	resolution
1.282258	0	2	TNFRSF18	0.3	1.139402	1.9E-118	0	FGFBP2	0.4
1.814899	0	2	MIB2	0.3	0.643382	1.6E-115	0	NKG7	0.4
2.16658	0	2	MMP23B	0.3	0.59918	4.04E-97	0	TMSB4X	0.4
-88.2172	0	2	RPL22	0.3	1.068942	6.54E-92	0	S100A4	0.4
-296.477	1	2	HLA-DPA1	0.3	0.779893	8.43E-83	0	CST7	0.4
-49.9625	0	2	CCL5	0.3	0.641623	3.23E-74	0	TMSB10	0.4
37.51595	0	2	FGFBP2	0.3	0.733848	2.58E-66	0	GZMB	0.4
-17.0769	0	2	GZMH	0.3	0.897122	2.50E-64	0	S100A6	0.4
-2.91919	0	2	GZMB	0.3	0.628575	3.12E-62	0	PFN1	0.4
15.72819	0	2	SPON2	0.3	0.758137	1.31E-58	0	SH3BGRL3	0.4
10.13304	0	2	CST7	0.3	0.64864	5.59E-56	0	SERF2	0.4
4.080785	0	2	IGFBP7	0.3	0.608905	6.43E-52	0	CFL1	0.4
25.46086	0	2	PRF1	0.3	0.590429	1.72E-51	0	SPON2	0.4
-40.2245	0	2	FCGR3A	0.3	0.636502	8.14E-48	0	ACTB	0.4
3.344678	0	2	XCL1	0.3	1.211455	1.46E-45	0	LGALS1	0.4
-16.7347	0	2	CD44	0.3	0.593891	1.63E-45	0	ARPC2	0.4
-88.2099	0	2	COTL1	0.3	0.782628	5.45E-44	0	CCL4	0.4
-1.53304	0	2	PTAFR	0.3	0.604101	5.91E-44	0	MYL12A	0.4
1.444759	0	2	CCDC28B	0.3	0.601794	4.22E-33	0	GZMH	0.4
-8.05335	0	2	LCK	0.3	0.523064	1.12E-31	0	MYL6	0.4
-2.09494	0	2	AK2	0.3	0.846209	7.37E-28	0	AKR1C3	0.4
-1.67312	0	2	PHC2	0.3	0.82232	1.10E-27	0	TRBC1	0.4
1.577396	0	2	AC004865.2	0.3	0.572653	2.45E-27	0	CCL5	0.4
-5.75115	0	2	CSF3R	0.3	0.582544	1.39E-26	0	EFHD2	0.4
-5.92632	0	2	PABPC4	0.3	0.605019	2.08E-26	0	EMP3	0.4
-8.97666	0	2	PPT1	0.3	0.606699	1.76E-25	0	PPDPF	0.4
-3.22218	0	2	SMAP2	0.3	0.680354	4.84E-25	0	DBI	0.4
-1.04494	0	2	CITED4	0.3	0.579422	6.51E-25	0	RARRES3	0.4
-1.92467	0	2	ARMH1	0.3	0.694029	1.81E-24	0	PPP1R18	0.4
-158.914	0	2	RPS8	0.3	0.513495	8.92E-24	0	IGFBP7	0.4
-1.56582	0	2	PLK3	0.3	0.667164	1.23E-22	0	C12orf75	0.4
-15.7822	0	2	NASP	0.3	0.522596	7.02E-22	0	SUB1	0.4
-1.50338	0	2	USP1	0.3	0.551032	2.01E-21	0	ABHD17A	0.4
2.543206	0	2	JAK1	0.3	0.590776	1.83E-20	0	PRR13	0.4
-1.08505	0	2	PDE4B	0.3	0.508158	4.18E-20	0	TTC38	0.4
-8.05779	0	2	AC103591.3	0.3	0.566232	3.15E-18	0	CEBPB	0.4
-1.11465	0	2	IFI44L	0.3	0.58709	8.05E-18	0	GNG2	0.4
3.464724	0	2	GBP5	0.3	0.629252	4.27E-17	0	ASCL2	0.4
1.847111	0	2	TGFBR3	0.3	1.163422	4.90E-17	0	PTGDS	0.4
-1.26296	0	2	EVI5	0.3	0.50867	1.33E-16	0	UBL5	0.4
1.355396	0	2	VAV3	0.3	0.549507	8.54E-16	0	POMP	0.4
1.267311	0	2	HENMT1	0.3	0.541606	1.18E-15	0	ALOX5AP	0.4
-1.40089	0	2	SORT1	0.3	0.504963	1.81E-15	0	KLRB1	0.4
-20.3861	0	2	PSMA5	0.3	0.681749	2.23E-15	0	HDDC2	0.4
-1.03516	0	2	AHCYL1	0.3	0.555912	1.79E-13	0	IFI27L2	0.4
-1.62511	0	2	DRAM2	0.3	0.520374	1.43E-12	0	SCP2	0.4
1.153205	0	2	DENND2D	0.3	0.514491	3.72E-11	0	LAIR2	0.4
-13.7986	0	2	RHOC	0.3	0.778701	2.08E-08	0	MYOM2	0.4
-4.08999	0	2	CSDE1	0.3	0.500488	7.38E-07	0	CISD3	0.4
1.024486	0	2	TSPAN2	0.3	0.64459	0.062559	0	S100B	0.4

Table 20: The cluster biomarkers obtained from Bis-imputed data using Seurat.

avg_log2FC	p_val_adj	cluster	gene	resolution	avg_log2FC	p_val_adj	cluster	gene	resolution
2.05416	4.7E-263	1	GZMK	0.4	1.279408	9.90E-96	2	MALAT1	0.4
1.431511	1.6E-213	1	XCL1	0.4	1.569357	2.47E-90	2	MT-CO2	0.4
0.800633	2.4E-163	1	SPTSSB	0.4	1.483786	4.38E-86	2	MT-CO1	0.4
1.371508	1.7E-103	1	XCL2	0.4	1.511464	3.78E-84	2	MT-CO3	0.4
0.590663	5.1E-100	1	EEF1A1	0.4	1.642321	1.17E-78	2	MT-ND2	0.4
0.699525	1.4E-99	1	CAPG	0.4	1.557002	1.03E-76	2	MT-ND4	0.4
1.361506	4.48E-90	1	SELL	0.4	1.640919	7.07E-67	2	MT-ND3	0.4
0.657401	1.03E-84	1	RPS2	0.4	1.589611	2.61E-65	2	MT-ATP6	0.4
1.387313	8.84E-83	1	COTL1	0.4	1.584281	4.89E-63	2	MT-CYB	0.4
0.561892	1.49E-76	1	RPS15A	0.4	0.890936	7.60E-49	2	HLA-C	0.4
1.167486	2.92E-70	1	CD44	0.4	0.917761	1.18E-48	2	HLA-A	0.4
0.516229	5.39E-69	1	RPL13	0.4	1.309196	1.01E-34	2	MT-ND1	0.4
0.524732	1.27E-67	1	RPL13A	0.4	1.301771	2.49E-26	2	PRF1	0.4
0.627831	6.14E-66	1	RPS12	0.4	0.899651	2.57E-23	2	HLA-B	0.4
0.66419	3.13E-65	1	RPS18	0.4	1.495355	1.39E-19	2	MT-ND5	0.4
0.522851	7.55E-62	1	RPL32	0.4	0.663568	6.98E-17	2	HLA-E	0.4
0.715569	1.38E-60	1	TPT1	0.4	0.751973	9.45E-10	2	DDX5	0.4
0.603705	1.08E-59	1	RPS23	0.4	0.713831	4.69E-07	2	ITGB2	0.4
0.63041	5.55E-56	1	RPLP1	0.4	1.171512	3.77E-05	2	SUN2	0.4
0.508381	7.94E-56	1	RPL18A	0.4	1.215486	0.000105	2	MT-ND6	0.4
0.622419	4.52E-55	1	RPL10A	0.4	1.118794	0.00059	2	PTPRC	0.4
0.630241	1.85E-52	1	RPSA	0.4	1.353138	0.007045	2	SON	0.4
0.565451	5.55E-50	1	RPL37	0.4	0.968911	0.007141	2	MYO1F	0.4
0.57431	3.95E-48	1	RPS24	0.4	0.949565	0.079699	2	NEAT1	0.4
0.865984	3.77E-47	1	CMC1	0.4	1.22312	4.47E-06	2	CXCR4	0.4
0.764537	6.20E-45	1	IL7R	0.4	0.726693	0.000126	2	SPON2	0.4
0.511831	1.36E-44	1	RPS8	0.4	0.599101	0.000533	2	FCGR3A	0.4
0.538022	3.28E-41	1	RPL8	0.4	1.210052	0.000933	2	PDIA4	0.4
0.510877	6.26E-40	1	RPS21	0.4	0.539473	0.004316	2	ZBP1	0.4
0.970602	6.42E-40	1	NFKBIA	0.4					
0.536771	1.10E-39	1	RPS5	0.4					
0.727582	2.23E-38	1	KLRC1	0.4					
0.631166	3.39E-38	1	RPLP0	0.4					
0.992313	8.65E-38	1	DUSP2	0.4					
0.608109	1.22E-36	1	RPL36A	0.4					
1.02575	2.54E-32	1	FOS	0.4					
0.785475	5.32E-30	1	LTB	0.4					
0.527844	2.96E-29	1	CAPN12	0.4					
0.677983	3.53E-29	1	MAFF	0.4					
0.819723	1.50E-28	1	AREG	0.4					
0.737992	1.81E-28	1	IFITM3	0.4					
0.758336	2.62E-28	1	CD2	0.4					
0.590078	2.27E-26	1	EEF1B2	0.4					
0.798404	9.53E-24	1	DUSP1	0.4					
0.629616	3.81E-23	1	CD74	0.4					
0.553956	2.12E-17	1	SOCS1	0.4					
0.508434	1.46E-14	1	PIK3R1	0.4					
0.522154	2.16E-13	1	AC245014.3	0.4					
0.603312	1.85E-12	1	AC103591.3	0.4					
0.516391	0.024625	1	JUN	0.4					

Table 21: The cluster biomarkers obtained from Bis-imputed data using Seurat.

avg_log2FC	p_val_adj	cluster	gene	resolution	avg_log2FC	p_val_adj	cluster	gene	resolution
1.314455	0	2	TNFRSF18	0.5	1.347448	8.16E-107	0	FGFBP2	0.6
1.869094	0	2	MIB2	0.5	0.572325	2.27E-77	0	NKG7	0.6
2.187427	0	2	MMP23B	0.5	0.754586	1.69E-59	0	CST7	0.6
-96.5894	0	2	RPL22	0.5	0.911602	8.69E-56	0	S100A4	0.6
-11.407	0	2	RBP7	0.5	0.758116	1.67E-52	0	GZMB	0.6
-98.0219	0	2	COTL1	0.5	0.669526	1.40E-42	0	SPON2	0.6
-16.6449	0	2	CD44	0.5	0.798517	1.99E-37	0	S100A6	0.6
3.403173	0	2	XCL1	0.5	0.690671	2.69E-35	0	FCGR3A	0.6
-40.1573	0	2	FCGR3A	0.5	0.912666	3.30E-33	0	GZMH	0.6
22.55794	0	2	PRF1	0.5	0.512223	3.27E-30	0	CFL1	0.6
-12.5349	0	2	FGFBP2	0.5	0.548063	6.46E-30	0	ARPC2	0.6
-18.9407	0	2	GZMH	0.5	0.568089	1.08E-28	0	ACTB	0.6
-3.01231	0	2	GZMB	0.5	0.708545	5.59E-28	0	CCL4	0.6
15.77026	0	2	SPON2	0.5	0.577017	6.90E-28	0	SH3BGRL3	0.6
10.20076	0	2	CST7	0.5	0.997867	1.30E-24	0	LGALS1	0.6
4.032438	0	2	IGFBP7	0.5	0.709827	1.30E-20	0	PRSS23	0.6
-1.52066	0	2	PTAFR	0.5	0.563859	2.15E-18	0	CCL5	0.6
1.367303	0	2	CCDC28B	0.5	0.782984	1.49E-16	0	TRBC1	0.6
-8.01294	0	2	LCK	0.5	0.587897	1.71E-16	0	EFHD2	0.6
-1.66154	0	2	PHC2	0.5	0.760218	2.86E-16	0	AKR1C3	0.6
1.62397	0	2	AC004865.2	0.5	0.638816	2.64E-14	0	TTC38	0.6
-5.74158	0	2	CSF3R	0.5	0.659858	3.11E-14	0	PPP1R18	0.6
1.011862	0	2	FHL3	0.5	0.524935	5.93E-14	0	IGFBP7	0.6
-5.90159	0	2	PABPC4	0.5	0.675967	1.74E-12	0	FCRL6	0.6
-8.97548	0	2	PPT1	0.5	0.500075	3.19E-12	0	RARRES3	0.6
-3.18631	0	2	SMAP2	0.5	0.656905	5.71E-12	0	ASCL2	0.6
-1.03222	0	2	CITED4	0.5	0.912666	3.30E-33	0	GZMH	0.6
-9.75177	0	2	EBNA1BP2	0.5	0.758116	1.67E-52	0	GZMB	0.6
-1.93677	0	2	ARMH1	0.5	0.570564	1.04E-11	0	CEBPB	0.6
-158.82	0	2	RPS8	0.5	0.576122	4.93E-11	0	C12orf75	0.6
-1.55542	0	2	PLK3	0.5	0.530498	4.18E-10	0	DBI	0.6
-17.5118	0	2	NASP	0.5	0.606027	1.25E-09	0	CX3CR1	0.6
-1.68863	0	2	USP1	0.5	0.861282	1.85E-09	0	PTGDS	0.6
2.530196	0	2	JAK1	0.5	0.539493	2.03E-09	0	DHRS7	0.6
-1.06216	0	2	PDE4B	0.5	0.576908	3.17E-09	0	GNG2	0.6
-8.03444	0	2	AC103591.3	0.5	0.514365	2.10E-08	0	ALOX5AP	0.6
-1.03724	0	2	IFI44L	0.5	0.544578	7.77E-07	0	S100A11	0.6
3.540257	0	2	GBP5	0.5	0.55756	1.70E-06	0	IFI27L2	0.6
1.825025	0	2	TGFBR3	0.5	0.563005	3.14E-06	0	HDDC2	0.6
-1.25513	0	2	EVI5	0.5	0.524376	0.000726	0	GIMAP7	0.6
1.391991	0	2	VAV3	0.5	0.548419	0.025504	0	MYOM2	0.6
1.273495	0	2	HENMT1	0.5	2.224567	6.0E-259	1	GZMK	0.6
-1.39261	0	2	SORT1	0.5	1.488812	9.3E-184	1	XCL1	0.6
-21.0458	0	2	PSMA5	0.5	0.817828	7.1E-133	1	SPTSSB	0.6
-1.02739	0	2	AHCYL1	0.5	0.588523	6.84E-83	1	EEF1A1	0.6
-1.59228	0	2	DRAM2	0.5	1.305972	3.98E-80	1	XCL2	0.6
1.102147	0	2	DENND2D	0.5	0.676046	9.92E-77	1	CAPG	0.6
-13.7411	0	2	RHOC	0.5	1.392596	9.27E-71	1	SELL	0.6
-4.06533	0	2	CSDE1	0.5	0.638478	4.31E-68	1	RPS2	0.6
1.043233	0	2	TSPAN2	0.5					

Table 22: The cluster biomarkers obtained from Bis-imputed data using Seurat.

avg_log2FC	p_val_adj	cluster	gene	resolution	avg_log2FC	p_val_adj	cluster	gene	resolution
1.375711	9.4E-110	0	FGFBP2	0.7	0.622687	2.16E-67	1	RPS2	0.7
0.566137	1.33E-76	0	NKG7	0.7	0.53926	6.88E-65	1	RPS15A	0.7
0.77567	2.76E-62	0	CST7	0.7	1.203572	4.59E-63	1	CD44	0.7
0.938219	2.75E-57	0	S100A4	0.7	1.236745	2.26E-61	1	COTL1	0.7
0.77489	1.11E-53	0	GZMB	0.7	0.523771	2.02E-60	1	RPL13A	0.7
0.714638	1.24E-46	0	SPON2	0.7	0.505056	9.44E-60	1	RPL13	0.7
0.849425	1.07E-41	0	S100A6	0.7	0.831089	1.62E-53	1	IL7R	0.7
0.68455	5.52E-36	0	FCGR3A	0.7	0.592038	7.97E-53	1	RPS12	0.7
0.930611	1.76E-35	0	GZMH	0.7	0.700898	5.20E-52	1	TPT1	0.7
0.602034	3.03E-32	0	ACTB	0.7	0.640111	1.41E-51	1	RPS18	0.7
0.512678	2.84E-30	0	CFL1	0.7	0.501036	9.36E-50	1	RPL32	0.7
0.752888	3.63E-30	0	CCL4	0.7	0.586096	1.19E-49	1	RPS23	0.7
0.585673	3.58E-28	0	SH3BGRL3	0.7	0.604847	2.56E-45	1	RPL10A	0.7
0.527451	4.03E-28	0	ARPC2	0.7	0.869667	1.85E-40	1	CMC1	0.7
1.124926	1.49E-27	0	LGALS1	0.7	0.557458	5.75E-40	1	RPLP1	0.7
0.719034	1.80E-21	0	PRSS23	0.7	0.580891	5.18E-39	1	RPSA	0.7
0.803879	6.28E-18	0	AKR1C3	0.7	0.522004	2.19E-38	1	RPL37	0.7
0.553673	1.27E-17	0	CCL5	0.7	1.064426	1.34E-37	1	DUSP2	0.7
0.602306	1.80E-17	0	EFHD2	0.7	1.069436	4.29E-37	1	NFKBIA	0.7
0.554719	2.37E-15	0	IGFBP7	0.7	0.521202	1.59E-34	1	RPS24	0.7
0.75214	2.98E-15	0	TRBC1	0.7	1.129502	6.11E-34	1	FOS	0.7
0.644529	1.57E-14	0	TTC38	0.7	0.740191	2.24E-32	1	KLRC1	0.7
0.650641	5.89E-14	0	PPP1R18	0.7	1.017292	3.81E-32	1	LTB	0.7
0.57261	1.45E-13	0	ABHD17A	0.7	0.506086	1.19E-31	1	RPL8	0.7
0.630078	3.41E-13	0	C12orf75	0.7	0.594316	1.42E-28	1	RPLP0	0.7
0.597273	5.05E-13	0	DBI	0.7	0.904708	1.98E-28	1	AREG	0.7
0.570173	7.29E-13	0	ADGRG1	0.7	0.572772	5.59E-28	1	RPL36A	0.7
0.5182	1.36E-12	0	PPDPF	0.7	0.927312	3.24E-26	1	DUSP1	0.7
0.664311	1.85E-12	0	FCRL6	0.7	0.722808	2.31E-23	1	MAFF	0.7
0.516638	4.23E-12	0	EMP3	0.7	0.567903	1.72E-20	1	EEF1B2	0.7
0.642149	1.53E-11	0	ASCL2	0.7	0.707818	4.00E-20	1	IFITM3	0.7
0.578391	1.57E-11	0	CEBPB	0.7	0.541772	8.72E-20	1	MCUB	0.7
0.923581	2.44E-11	0	PTGDS	0.7	0.737381	4.16E-19	1	CD2	0.7
0.572811	1.36E-10	0	DHRS7	0.7	0.600546	8.71E-17	1	CD74	0.7
0.632121	1.64E-10	0	CX3CR1	0.7	0.550905	1.95E-16	1	AC044849.1	0.7
0.577063	1.31E-08	0	GNG2	0.7	0.598408	6.98E-16	1	SOCS1	0.7
0.519019	2.27E-08	0	ALOX5AP	0.7	0.633963	2.62E-15	1	AC245014.3	0.7
0.595867	1.73E-07	0	IFI27L2	0.7	0.566923	9.09E-15	1	ZFP36L2	0.7
0.553603	6.83E-07	0	S100A11	0.7	0.512271	8.77E-13	1	IFRD1	0.7
0.582944	7.63E-07	0	HDDC2	0.7	0.651107	3.96E-11	1	AC103591.3	0.7
0.523055	0.000971	0	GIMAP7	0.7	0.51738	3.13E-10	1	PIK3R1	0.7
0.561242	0.007122	0	MYOM2	0.7	0.523042	6.49E-07	1	PPP1R15A	0.7
2.17447	8.81E-255	1	GZMK	0.7	0.585535	0.000149	1	IER2	0.7
1.466618	6.99E-194	1	XCL1	0.7	0.557718	0.097673	1	JUN	0.7
0.816821	7.30E-137	1	SPTSSB	0.7	1.528857	3.51E-37	2	MT-ND2	0.7
1.322728	1.03E-84	1	XCL2	0.7	1.387268	9.88E-35	2	MT-CO2	0.7
0.579613	3.42E-84	1	EEF1A1	0.7	1.341658	1.99E-31	2	MT-CO1	0.7
0.663981	3.23E-77	1	CAPG	0.7	1.460748	1.26E-29	2	MT-ATP6	0.7
1.40286	6.77E-76	1	SELL	0.7	1.005741	5.92E-28	2	MALAT1	0.7
					1.502354	1.12E-16	2	PRF1	0.7

Table 23: The cluster biomarkers obtained from Bis-imputed data using Seurat.

avg_log2FC	p_val_adj	cluster	gene	resolution	avg_log2FC	p_val_adj	cluster	gene	resolution
1.334078	8.2E-105	0	FGFBP2	0.8	0.647231	1.3E-109	0	NKG7	0.9
0.570263	7.36E-77	0	NKG7	0.8	1.096363	7.77E-107	0	FGFBP2	0.9
0.754814	3.05E-59	0	CST7	0.8	0.622654	2.42E-94	0	TMSB4X	0.9
0.906603	6.74E-55	0	S100A4	0.8	1.045595	6.70E-83	0	S100A4	0.9
0.74749	4.81E-51	0	GZMB	0.8	0.694263	5.87E-80	0	TMSB10	0.9
0.651684	7.29E-42	0	SPON2	0.8	0.75378	6.57E-73	0	CST7	0.9
0.793467	1.10E-36	0	S100A6	0.8	0.725893	9.97E-62	0	GZMB	0.9
0.685049	1.08E-34	0	FCGR3A	0.8	0.637348	2.47E-59	0	PFN1	0.9
0.927688	3.84E-34	0	GZMH	0.8	0.86292	2.17E-56	0	S100A6	0.9
0.503154	4.41E-29	0	CFL1	0.8	0.672873	3.38E-55	0	SERF2	0.9
0.568325	1.39E-28	0	ACTB	0.8	0.760687	1.37E-54	0	SH3BGRL3	0.9
0.530803	2.64E-28	0	ARPC2	0.8	0.635243	1.93E-51	0	CFL1	0.9
0.70546	1.09E-27	0	CCL4	0.8	0.640458	4.09E-45	0	ACTB	0.9
0.571872	6.03E-27	0	SH3BGRL3	0.8	1.261027	9.96E-44	0	LGALS1	0.9
0.98162	7.34E-24	0	LGALS1	0.8	0.601393	5.94E-43	0	ARPC2	0.9
0.685663	1.36E-19	0	PRSS23	0.8	0.516277	3.45E-41	0	SPON2	0.9
0.562742	3.45E-18	0	CCL5	0.8	0.599687	1.47E-39	0	MYL12A	0.9
0.585224	3.03E-16	0	EFHD2	0.8	0.730477	7.42E-37	0	CCL4	0.9
0.780333	4.70E-16	0	TRBC1	0.8	0.577215	3.03E-30	0	GZMH	0.9
0.756798	4.81E-16	0	AKR1C3	0.8	0.530553	4.64E-30	0	MYL6	0.9
0.653081	3.81E-14	0	PPP1R18	0.8	0.850557	2.97E-27	0	TRBC1	0.9
0.526912	6.59E-14	0	IGFBP7	0.8	0.730244	1.14E-26	0	DBI	0.9
0.606293	3.55E-13	0	TTC38	0.8	0.85001	2.53E-26	0	AKR1C3	0.9
0.677344	2.19E-12	0	FCRL6	0.8	0.605079	5.67E-25	0	RARRES3	0.9
0.560617	2.70E-12	0	ADGRG1	0.8	0.555793	1.08E-24	0	CCL5	0.9
0.564223	2.25E-11	0	CEBPB	0.8	0.612175	2.13E-24	0	PPDPF	0.9
0.530182	3.09E-11	0	ABHD17A	0.8	0.577415	3.43E-24	0	EFHD2	0.9
0.579172	4.05E-11	0	C12orf75	0.8	0.703386	1.54E-23	0	PPP1R18	0.9
0.619982	1.36E-10	0	ASCL2	0.8	0.512902	1.05E-22	0	IGFBP7	0.9
0.551168	5.94E-10	0	DHRS7	0.8	0.684539	2.36E-22	0	C12orf75	0.9
0.521883	8.15E-10	0	DBI	0.8	0.540306	5.08E-22	0	SUB1	0.9
0.603821	1.72E-09	0	CX3CR1	0.8	0.511475	8.02E-22	0	PPIA	0.9
0.850706	5.63E-09	0	PTGDS	0.8	0.555792	2.05E-21	0	EMP3	0.9
0.570087	8.38E-09	0	GNG2	0.8	0.512021	2.47E-21	0	AES	0.9
0.516468	1.94E-08	0	ALOX5AP	0.8	0.611449	8.49E-20	0	PRR13	0.9
0.539045	1.50E-06	0	S100A11	0.8	0.543161	1.19E-19	0	ABHD17A	0.9
0.553074	2.79E-06	0	IFI27L2	0.8	0.623073	5.53E-19	0	POMP	0.9
0.505844	6.79E-06	0	C1orf21	0.8	0.515636	1.48E-18	0	UQCR11	0.9
0.544153	1.73E-05	0	HDDC2	0.8	0.532851	1.66E-17	0	ATP5ME	0.9
0.511569	0.001985	0	GIMAP7	0.8	0.541187	3.07E-17	0	UBL5	0.9
0.527664	0.065568	0	MYOM2	0.8	0.659535	5.88E-17	0	ASCL2	0.9
2.223597	1.09E-258	1	GZMK	0.8	0.504428	9.19E-17	0	UCP2	0.9
1.467974	9.63E-184	1	XCL1	0.8	1.152129	2.59E-16	0	PTGDS	0.9
0.824933	1.72E-134	1	SPTSSB	0.8	0.516749	4.20E-16	0	CEBPB	0.9
0.591954	1.45E-83	1	EEF1A1	0.8	0.709039	3.64E-15	0	HDDC2	0.9
1.313864	2.18E-80	1	XCL2	0.8	0.500078	1.98E-14	0	KLRB1	0.9
0.682323	1.03E-77	1	CAPG	0.8	0.54757	2.33E-14	0	GNG2	0.9
1.398598	1.01E-69	1	SELL	0.8	0.500672	1.49E-13	0	NDUFB7	0.9
0.639435	1.09E-67	1	RPS2	0.8	0.569937	1.42E-12	0	IFI27L2	0.9
0.551207	6.93E-64	1	RPS15A	0.8	0.507925	1.48E-11	0	SCP2	0.9
					0.530995	5.65E-11	0	S100A11	0.9

Table 24: The cluster biomarkers obtained from Bis-imputed data using Seurat.

avg_log2FC	p_val_adj	cluster	gene	resolution	avg_log2FC	p_val_adj	cluster	gene	resolution
0.513101	2.15E-10	0	C1orf21	0.9	0.585612	1.25E-14	1	AC245014.3	0.9
0.524133	2.86E-10	0	LAIR2	0.9	0.5404	4.00E-14	1	ZFP36L2	0.9
0.75892	1.72E-07	0	MYOM2	0.9	0.549963	4.38E-13	1	PIK3R1	0.9
0.640612	0.040193	0	S100B	0.9	0.637868	6.76E-11	1	AC103591.3	0.9
2.133327	6.9E-259	1	GZMK	0.9	0.509822	2.54E-07	1	TAGLN2	0.9
1.393531	1.4E-183	1	XCL1	0.9	0.544125	0.000149	1	IER2	0.9
0.790792	2.2E-139	1	SPTSSB	0.9	0.522216	0.021347	1	JUN	0.9
0.598347	1.41E-89	1	EEF1A1	0.9	1.25875	4.15E-94	2	MALAT1	0.9
1.316063	1.89E-89	1	XCL2	0.9	1.564839	6.97E-90	2	MT-CO2	0.9
1.357665	1.10E-77	1	SELL	0.9	1.47589	8.44E-85	2	MT-CO1	0.9
0.640144	4.24E-77	1	CAPG	0.9	1.519478	2.29E-84	2	MT-CO3	0.9
0.647538	3.61E-73	1	RPS2	0.9	1.650817	4.22E-78	2	MT-ND2	0.9
0.565448	1.59E-68	1	RPS15A	0.9	1.560277	1.78E-77	2	MT-ND4	0.9
1.24562	1.41E-64	1	COTL1	0.9	1.63127	1.75E-65	2	MT-ND3	0.9
1.158935	2.38E-63	1	CD44	0.9	1.597133	9.69E-64	2	MT-ATP6	0.9
0.536691	1.68E-62	1	RPL13A	0.9	1.577673	1.70E-60	2	MT-CYB	0.9
0.522547	2.49E-62	1	RPL13	0.9	0.918682	2.96E-48	2	HLA-A	0.9
0.503329	5.10E-57	1	RPL34	0.9	0.876887	3.66E-48	2	HLA-C	0.9
0.724844	1.29E-55	1	TPT1	0.9	1.32687	1.23E-34	2	MT-ND1	0.9
0.603635	8.00E-55	1	RPS12	0.9	1.286434	4.61E-26	2	PRF1	0.9
0.646315	3.59E-53	1	RPS18	0.9	0.921511	6.46E-25	2	HLA-B	0.9
0.508174	2.76E-50	1	RPL32	0.9	1.50576	1.91E-20	2	MT-ND5	0.9
0.595548	3.87E-50	1	RPS23	0.9	0.662165	1.36E-16	2	HLA-E	0.9
0.773082	8.78E-49	1	IL7R	0.9	0.739149	3.10E-09	2	DDX5	0.9
0.612119	2.55E-45	1	RPL10A	0.9	0.706811	1.10E-06	2	ITGB2	0.9
0.587048	3.12E-45	1	RPLP1	0.9	1.225385	5.11E-06	2	MT-ND6	0.9
0.882431	4.58E-44	1	CMC1	0.9	1.14944	2.41E-05	2	SUN2	0.9
0.61125	1.87E-43	1	RPSA	0.9	1.124715	0.000204	2	PTPRC	0.9
0.538874	9.40E-41	1	RPL37	0.9	1.349595	0.00525	2	SON	0.9
0.553935	1.63E-39	1	RPS24	0.9	0.950257	0.006497	2	MYO1F	0.9
1.023753	3.86E-39	1	NFKBIA	0.9	0.929996	0.125556	2	NEAT1	0.9
1.037298	1.25E-36	1	DUSP2	0.9	1.220859	7.77E-05	2	CXCR4	0.9
0.53491	3.62E-35	1	RPL8	0.9	0.503853	0.000144	2	CTSW	0.9
1.091752	5.64E-34	1	FOS	0.9	0.719382	0.000477	2	SPON2	0.9
0.717251	4.99E-33	1	KLRC1	0.9	0.581516	0.001432	2	FCGR3A	0.9
0.947882	6.60E-33	1	LTB	0.9	1.24662	0.001803	2	PDIA4	0.9
0.623646	4.42E-32	1	RPLP0	0.9	1.13204	0.002446	2	HSPA5	0.9
0.506482	2.86E-31	1	RPS5	0.9	0.89952	0.008221	2	PDIA3	0.9
0.588186	1.45E-30	1	RPL36A	0.9					
0.873592	1.27E-29	1	AREG	0.9					
0.741061	2.15E-29	1	MAFF	0.9					
0.559249	3.36E-27	1	CAPN12	0.9					
0.876283	1.72E-26	1	DUSP1	0.9					
0.752657	5.86E-23	1	CD2	0.9					
0.713544	8.85E-23	1	IFITM3	0.9					
0.578221	8.51E-22	1	EEF1B2	0.9					
0.532166	2.92E-18	1	AC044849.1	0.9					
0.596643	7.88E-18	1	CD74	0.9					
0.517761	2.94E-17	1	TCF7	0.9					
0.590627	9.90E-17	1	SOCS1	0.9					

Table 25: The cluster biomarkers obtained from Bis-imputed data using Seurat.

avg_log2FC	p_val_adj	cluster	gene	resolution	avg_log2FC	p_val_adj	cluster	gene	resolution
0.646987	8.7E-110	0	NKG7	1.0	0.514009	2.72E-10	0	C1orf21	1.0
1.095802	1.5E-106	0	FGFBP2	1.0	0.521231	3.82E-10	0	LAIR2	1.0
0.621573	7.08E-94	0	TMSB4X	1.0	0.75596	2.19E-07	0	MYOM2	1.0
1.041573	3.62E-82	0	S100A4	1.0	0.63736	0.045945	0	S100B	1.0
0.693478	9.04E-80	0	TMSB10	1.0	2.132685	1.0E-256	1	GZMK	1.0
0.751118	2.80E-72	0	CST7	1.0	1.395956	2.8E-184	1	XCL1	1.0
0.722733	4.26E-61	0	GZMB	1.0	0.792363	6.7E-140	1	SPTSSB	1.0
0.63304	2.01E-58	0	PFN1	1.0	0.600343	3.70E-90	1	EEF1A1	1.0
0.869722	7.88E-57	0	S100A6	1.0	1.319204	7.42E-90	1	XCL2	1.0
0.674835	1.50E-55	0	SERF2	1.0	1.361717	3.27E-78	1	SELL	1.0
0.764049	7.21E-55	0	SH3BGRL3	1.0	0.641554	2.12E-77	1	CAPG	1.0
0.633773	4.66E-51	0	CFL1	1.0	0.646746	1.57E-72	1	RPS2	1.0
0.644855	1.43E-45	0	ACTB	1.0	0.569354	2.05E-69	1	RPS15A	1.0
0.60418	3.00E-43	0	ARPC2	1.0	1.248598	6.05E-65	1	COTL1	1.0
1.254723	4.86E-43	0	LGALS1	1.0	1.166862	9.58E-65	1	CD44	1.0
0.513263	6.41E-41	0	SPON2	1.0	0.525025	4.57E-63	1	RPL13	1.0
0.597204	3.51E-39	0	MYL12A	1.0	0.535766	3.53E-62	1	RPL13A	1.0
0.731735	6.60E-37	0	CCL4	1.0	0.508827	2.68E-58	1	RPL34	1.0
0.535725	1.19E-30	0	MYL6	1.0	0.727839	2.10E-56	1	TPT1	1.0
0.579709	1.88E-30	0	GZMH	1.0	0.606123	1.67E-55	1	RPS12	1.0
0.848774	4.37E-27	0	TRBC1	1.0	0.647846	2.21E-53	1	RPS18	1.0
0.733323	6.96E-27	0	DBI	1.0	0.512231	1.94E-51	1	RPL32	1.0
0.848529	3.41E-26	0	AKR1C3	1.0	0.597482	1.49E-50	1	RPS23	1.0
0.55498	1.48E-24	0	CCL5	1.0	0.774845	5.30E-49	1	IL7R	1.0
0.60009	1.74E-24	0	RARRES3	1.0	0.615006	7.18E-46	1	RPL10A	1.0
0.581744	1.86E-24	0	EFHD2	1.0	0.586237	9.57E-45	1	RPLP1	1.0
0.612914	2.15E-24	0	PPDPF	1.0	0.885354	3.58E-44	1	CMC1	1.0
0.700848	2.66E-23	0	PPP1R18	1.0	0.607313	2.01E-42	1	RPSA	1.0
0.511281	1.25E-22	0	IGFBP7	1.0	0.536906	4.81E-40	1	RPL37	1.0
0.676345	8.07E-22	0	C12orf75	1.0	0.554127	1.71E-39	1	RPS24	1.0
0.564243	1.16E-21	0	EMP3	1.0	1.024693	2.73E-39	1	NFKBIA	1.0
0.533389	1.68E-21	0	SUB1	1.0	1.035888	2.37E-36	1	DUSP2	1.0
0.511757	2.77E-21	0	AES	1.0	0.537326	2.21E-35	1	RPL8	1.0
0.504538	3.75E-21	0	PPIA	1.0	1.095142	3.10E-34	1	FOS	1.0
0.544654	8.87E-20	0	ABHD17A	1.0	0.956737	6.33E-34	1	LTB	1.0
0.610632	1.05E-19	0	PRR13	1.0	0.714882	7.32E-33	1	KLRC1	1.0
0.620233	9.62E-19	0	POMP	1.0	0.622804	5.66E-32	1	RPLP0	1.0
0.517555	1.20E-18	0	UQCR11	1.0	0.50568	2.94E-31	1	RPS5	1.0
0.548702	1.13E-17	0	UBL5	1.0	0.583708	9.25E-30	1	RPL36A	1.0
0.534028	1.42E-17	0	ATP5ME	1.0	0.743582	1.49E-29	1	MAFF	1.0
0.515626	3.89E-17	0	UCP2	1.0	0.870946	2.86E-29	1	AREG	1.0
0.657311	8.09E-17	0	ASCL2	1.0	0.550842	2.76E-26	1	CAPN12	1.0
1.146973	3.40E-16	0	PTGDS	1.0	0.875561	5.75E-26	1	DUSP1	1.0
0.513304	6.31E-16	0	CEBPB	1.0	0.726647	3.07E-23	1	IFITM3	1.0
0.698546	1.45E-14	0	HDDC2	1.0	0.752222	1.04E-22	1	CD2	1.0
0.511995	5.90E-14	0	NDUFB7	1.0	0.579765	5.85E-22	1	EEF1B2	1.0
0.538728	7.73E-14	0	GNG2	1.0	0.534529	2.03E-18	1	AC044849.1	1.0
0.572811	1.01E-12	0	IFI27L2	1.0	0.594351	1.70E-17	1	CD74	1.0
0.508485	1.34E-11	0	SCP2	1.0	0.519781	2.14E-17	1	TCF7	1.0
0.525437	7.68E-11	0	S100A11	1.0	0.592534	8.13E-17	1	SOCS1	1.0

Table 26: The cluster biomarkers obtained from Bis-imputed data using Seurat.

avg_log2FC	p_val_adj	cluster	gene	resolution	avg_log2FC	p_val_adj	cluster	gene	resolution
1.354653	7.6E-106	0	FGFBP2	1.1	1.221708	1.04E-62	1	CD44	1.1
0.570187	1.90E-75	0	NKG7	1.1	0.540488	6.40E-61	1	RPL13A	1.1
0.775065	1.54E-60	0	CST7	1.1	1.277163	9.84E-61	1	COTL1	1.1
0.906457	2.24E-54	0	S100A4	1.1	0.523166	2.38E-60	1	RPL13	1.1
0.767522	1.24E-52	0	GZMB	1.1	0.866152	5.14E-55	1	IL7R	1.1
0.705374	2.05E-43	0	SPON2	1.1	0.717097	4.73E-52	1	TPT1	1.1
0.796744	1.10E-36	0	S100A6	1.1	0.660072	4.85E-52	1	RPS18	1.1
0.722798	1.61E-36	0	FCGR3A	1.1	0.597711	1.61E-50	1	RPS23	1.1
0.917781	9.06E-33	0	GZMH	1.1	0.599331	3.14E-50	1	RPS12	1.1
0.541735	5.85E-29	0	ARPC2	1.1	0.512979	7.04E-49	1	RPL32	1.1
0.72699	8.75E-29	0	CCL4	1.1	0.631703	2.11E-47	1	RPL10A	1.1
0.501577	1.06E-28	0	CFL1	1.1	0.874344	8.47E-39	1	CMC1	1.1
0.597918	2.44E-28	0	SH3BGRL3	1.1	0.565656	2.60E-38	1	RPLP1	1.1
0.559087	2.67E-27	0	ACTB	1.1	0.534718	3.90E-38	1	RPL37	1.1
1.019156	6.39E-25	0	LGALS1	1.1	0.580817	7.04E-38	1	RPSA	1.1
0.703493	3.96E-20	0	PRSS23	1.1	1.108965	1.16E-36	1	NFKBIA	1.1
0.572562	1.33E-18	0	CCL5	1.1	0.506292	1.43E-36	1	RPS8	1.1
0.609925	3.69E-17	0	EFHD2	1.1	1.092213	2.90E-36	1	DUSP2	1.1
0.802877	1.10E-16	0	TRBC1	1.1	0.540375	6.95E-35	1	RPS24	1.1
0.747993	1.74E-15	0	AKR1C3	1.1	1.143236	8.08E-33	1	FOS	1.1
0.534915	5.05E-14	0	IGFBP7	1.1	0.519756	3.97E-31	1	RPL8	1.1
0.657335	8.60E-14	0	TTC38	1.1	0.996075	6.38E-31	1	LTB	1.1
0.645552	1.15E-13	0	PPP1R18	1.1	0.506148	1.16E-29	1	RPS5	1.1
0.684893	6.56E-12	0	ASCL2	1.1	0.72488	1.30E-29	1	KLRC1	1.1
0.559017	7.18E-12	0	ADGRG1	1.1	0.970119	4.78E-27	1	DUSP1	1.1
0.666458	9.34E-12	0	FCRL6	1.1	0.786288	6.26E-27	1	MAFF	1.1
0.602	9.91E-12	0	CEBPB	1.1	0.590589	6.46E-27	1	RPLP0	1.1
0.597496	9.94E-12	0	C12orf75	1.1	0.583867	2.03E-26	1	RPL36A	1.1
0.521533	5.36E-11	0	ABHD17A	1.1	0.879179	2.77E-24	1	AREG	1.1
0.52772	9.87E-10	0	DBI	1.1	0.584911	2.42E-20	1	EEF1B2	1.1
0.588416	1.98E-09	0	GNG2	1.1	0.714437	1.98E-19	1	IFITM3	1.1
0.60543	4.14E-09	0	CX3CR1	1.1	0.725211	5.01E-18	1	CD2	1.1
0.834899	6.64E-09	0	PTGDS	1.1	0.528	6.83E-17	1	MCUB	1.1
0.529049	7.74E-09	0	DHRS7	1.1	0.502085	9.68E-17	1	TCF7	1.1
0.527756	5.41E-08	0	ALOX5AP	1.1	0.660176	1.44E-15	1	AC245014.3	1.1
0.554833	3.50E-06	0	IFI27L2	1.1	0.555885	1.52E-15	1	AC044849.1	1.1
0.556432	8.56E-06	0	HDDC2	1.1	0.618088	3.45E-15	1	SOCS1	1.1
0.519558	9.62E-06	0	S100A11	1.1	0.599261	6.77E-15	1	CD74	1.1
0.526749	0.001276	0	GIMAP7	1.1	0.579013	1.73E-14	1	ZFP36L2	1.1
0.518939	0.107488	0	MYOM2	1.1	0.546388	5.30E-14	1	IFRD1	1.1
2.226238	1.65E-258	1	GZMK	1.1	0.562747	8.60E-12	1	PIK3R1	1.1
1.473746	5.81E-186	1	XCL1	1.1	0.636474	4.53E-10	1	AC103591.3	1.1
0.832154	4.16E-136	1	SPTSSB	1.1	0.530139	2.16E-06	1	PPP1R15A	1.1
0.592512	2.16E-82	1	EEF1A1	1.1	0.615009	5.09E-05	1	IER2	1.1
0.688701	1.06E-78	1	CAPG	1.1	0.620183	0.00316	1	JUN	1.1
1.303107	3.76E-77	1	XCL2	1.1	1.521558	5.28E-36	2	MT-ND2	1.1
1.399999	9.76E-71	1	SELL	1.1	1.383629	1.98E-33	2	MT-CO2	1.1
0.648752	2.50E-69	1	RPS2	1.1	1.338364	3.31E-30	2	MT-CO1	1.1
0.555269	1.92E-64	1	RPS15A	1.1	1.449963	2.24E-28	2	MT-ATP6	1.1
1.450493	1.06E-15	2	PRF1	1.1	0.992478	5.76E-27	2	MALAT1	1.1
					1.199645	5.51E-07	2	SPON2	1.1

Table 27: The cluster biomarkers obtained from Bis-imputed data using Seurat.

avg_log2FC	p_val_adj	cluster	gene	resolution	avg_log2FC	p_val_adj	cluster	gene	resolution
1.395215	4.76E-107	0	FGFBP2	1.2	0.541372	1.61E-61	1	RPL13A	1.2
0.579312	1.72E-76	0	NKG7	1.2	1.225269	1.81E-61	1	CD44	1.2
0.772228	7.48E-60	0	CST7	1.2	0.525768	7.34E-61	1	RPL13	1.2
0.909276	9.46E-54	0	S100A4	1.2	1.267935	1.13E-59	1	COTL1	1.2
0.776215	5.42E-53	0	GZMB	1.2	0.875612	6.79E-57	1	IL7R	1.2
0.758387	1.21E-45	0	SPON2	1.2	0.662222	5.84E-53	1	RPS18	1.2
0.770471	6.11E-39	0	FCGR3A	1.2	0.719088	2.54E-52	1	TPT1	1.2
0.80714	1.79E-36	0	S100A6	1.2	0.600036	5.86E-51	1	RPS23	1.2
0.969258	1.76E-34	0	GZMH	1.2	0.600131	2.15E-50	1	RPS12	1.2
0.509876	8.20E-29	0	CFL1	1.2	0.515439	7.86E-49	1	RPL32	1.2
0.60647	8.31E-29	0	SH3BGRL3	1.2	0.628616	1.90E-46	1	RPL10A	1.2
0.548133	1.50E-28	0	ARPC2	1.2	0.56517	1.53E-38	1	RPLP1	1.2
0.570224	4.23E-28	0	ACTB	1.2	0.878303	1.68E-38	1	CMC1	1.2
0.688142	1.64E-27	0	CCL4	1.2	0.535496	3.96E-38	1	RPL37	1.2
1.036673	8.67E-26	0	LGALS1	1.2	1.115136	9.91E-38	1	NFKBIA	1.2
0.767213	4.53E-21	0	PRSS23	1.2	0.57884	2.95E-37	1	RPSA	1.2
0.647549	6.87E-19	0	EFHD2	1.2	0.504258	2.55E-36	1	RPS8	1.2
0.585119	1.09E-18	0	CCL5	1.2	1.094973	4.58E-36	1	DUSP2	1.2
0.63687	3.97E-17	0	IGFBP7	1.2	0.533124	2.88E-33	1	RPS24	1.2
0.800287	2.95E-16	0	TRBC1	1.2	1.150453	2.93E-33	1	FOS	1.2
0.747	2.94E-15	0	AKR1C3	1.2	0.526073	1.65E-32	1	RPL8	1.2
0.672117	6.32E-14	0	TTC38	1.2	1.02521	4.01E-30	1	LTB	1.2
0.634875	1.15E-12	0	PPP1R18	1.2	0.721748	3.19E-29	1	KLRC1	1.2
0.631471	1.83E-12	0	ADGRG1	1.2	0.972763	4.42E-28	1	DUSP1	1.2
0.687215	2.42E-12	0	FCRL6	1.2	0.595257	2.58E-27	1	RPLP0	1.2
0.611259	4.25E-12	0	C12orf75	1.2	0.581348	2.06E-26	1	RPL36A	1.2
0.588368	1.13E-11	0	CEBPB	1.2	0.782848	2.22E-26	1	MAFF	1.2
0.673808	2.58E-11	0	ASCL2	1.2	0.88323	3.86E-24	1	AREG	1.2
0.507837	2.59E-10	0	ABHD17A	1.2	0.59578	3.43E-21	1	EEF1B2	1.2
0.52686	1.55E-09	0	DBI	1.2	0.742021	1.34E-18	1	CD2	1.2
0.635403	3.50E-09	0	CX3CR1	1.2	0.700365	3.12E-18	1	IFITM3	1.2
0.600769	8.36E-09	0	GNG2	1.2	0.530767	4.32E-17	1	MCUB	1.2
0.527629	1.46E-08	0	DHRS7	1.2	0.503022	8.18E-17	1	TCF7	1.2
0.812454	1.90E-08	0	PTGDS	1.2	0.626237	7.88E-16	1	SOCS1	1.2
0.521355	1.22E-07	0	ALOX5AP	1.2	0.663045	8.08E-16	1	AC245014.3	1.2
0.516877	1.68E-07	0	TRBC2	1.2	0.562681	9.85E-16	1	AC044849.1	1.2
0.552496	4.74E-06	0	LAIR2	1.2	0.590453	2.42E-15	1	ZFP36L2	1.2
0.556857	1.07E-05	0	HDDC2	1.2	0.600525	6.73E-15	1	CD74	1.2
0.537827	1.14E-05	0	KIR2DL3	1.2	0.556572	2.48E-14	1	IFRD1	1.2
0.542282	1.25E-05	0	IFI27L2	1.2	0.557829	2.12E-11	1	PIK3R1	1.2
0.521657	0.002152	0	GIMAP7	1.2	0.644738	1.18E-10	1	AC103591.3	1.2
2.233094	1.7E-261	1	GZMK	1.2	0.530892	3.30E-06	1	PPP1R15A	1.2
1.476095	2.2E-185	1	XCL1	1.2	0.624682	2.30E-05	1	IER2	1.2
0.833963	1.9E-136	1	SPTSSB	1.2	0.626672	0.002333	1	JUN	1.2
0.594473	5.14E-82	1	EEF1A1	1.2	1.535016	7.79E-34	2	MT-ND2	1.2
0.699176	4.33E-81	1	CAPG	1.2	1.381054	1.65E-30	2	MT-CO2	1.2
1.299768	2.08E-76	1	XCL2	1.2	1.305999	8.33E-29	2	MT-CO1	1.2
0.65114	5.60E-70	1	RPS2	1.2	1.488882	4.99E-27	2	MT-ATP6	1.2
1.395369	3.90E-69	1	SELL	1.2	1.352609	5.02E-25	2	MT-CO3	1.2
0.551133	1.25E-63	1	RPS15A	1.2	1.269175	1.14E-24	2	MT-ND4	1.2
1.173277	4.83E-05	2	SPON2	1.2	1.421874	2.09E-12	2	PRF1	1.2
